# Supplementary material for: Rumicidins are a family of mammalian host-defense peptides plugging the 70S ribosome exit tunnel
Source: Nat Commun. 2024 Oct 16;15:8925. doi: 10.1038/s41467-024-53309-y (PMC11484942; doi:10.1038/s41467-024-53309-y)

Supplementary Information

Rumicidins are a family of mammalian host-defense peptides plugging the 70S ribosome exit tunnel

Pavel V. Panteleev[1,*], Eugene B. Pichkur[2,*], Roman N. Kruglikov[1], Alena Paleskava[2], Olga V. Shulenina[2], Ilia A. Bolosov[1], Ivan V. Bogdanov[1], Victoria N. Safronova[1], Sergey V. Balandin[1], Valeriya I. Marina[3], Tatiana I. Kombarova[4], Olga V. Korobova[4], Olga V. Shamova[5], Alexander G. Myasnikov[2], Alexander I. Borzilov[4], Ilya A. Osterman[3,6], Petr V. Sergiev[3,6], Alexey A. Bogdanov[1,3], Olga A. Dontsova[1,3,6], Andrey L. Konevega[2,**], Tatiana V. Ovchinnikova[1,3,7,**]

[1] M.M. Shemyakin & Yu.A. Ovchinnikov Institute of Bioorganic Chemistry, the Russian Academy of Sciences, Miklukho-Maklaya str., 16/10, 117997 Moscow, Russia; p.v.panteleev@gmail.com (P.V.P.); kruglikov1911@mail.ru (R.N.K); bolosov@ibch.ru (I.A.B.); contraton@mail.ru (I.V.B.), victoria.saf@ibch.ru (V.N.S.); arenicin@mail.ru (S.V.B.); ovch@ibch.ru (T.V.O.);
[2] Petersburg Nuclear Physics Institute named by B.P. Konstantinov of NRC "Kurchatov Institute", Gatchina, Russia; pichkur_eb@pnpi.nrcki.ru (E.B.P.); shulenina_ov@pnpi.nrcki.ru (O.V.Shu); polesskova_ev@pnpi.nrcki.ru (A.P.); samag@mail.ru (A.G.M.); konevega_al@pnpi.nrcki.ru (A.L.K.);
[3] Lomonosov Moscow State University, Moscow, Russia; ymmo@mail.ru (V.I.M.); osterman@yandex.ru (I.A.O); petya@belozersky.msu.ru (P.V.S.); alexaaa1935@gmail.com (A.A.B.); olga.a.dontsova@gmail.com (O.A.D.); ovch@ibch.ru (T.V.O.);
[4] State Research Center for Applied Microbiology & Biotechnology (SRCAMB), Obolensk, Russia; kombarova@obolensk.org (T.I.K.); korobova@obolensk.org (O.V.K.); borzilov@obolensk.org (A.I.B.);
[5] Institute of Experimental Medicine, Saint Petersburg, Russia; oshamova@yandex.ru (O.V.Sha);
[6] Center of Life Sciences, Skolkovo Institute of Science and Technology, Skolkovo, Russia; osterman@yandex.ru (I.A.O); olga.a.dontsova@gmail.com (O.A.D.);
[7] Department of Biotechnology, I.M. Sechenov First Moscow State Medical University (Sechenov University), Moscow, Russia; ovch@ibch.ru (T.V.O.).
* These authors contributed equally to this work.
** Corresponding authors: Tatiana V. Ovchinnikova, email: ovch@ibch.ru,
Andrey L. Konevega, email: konevega_al@pnpi.nrcki.ru

This file includes:

I. Supplementary Figures (1 to 17)

II. Supplementary Tables (1 to 8)

III. Supplementary Discussion

IV. Supplementary References

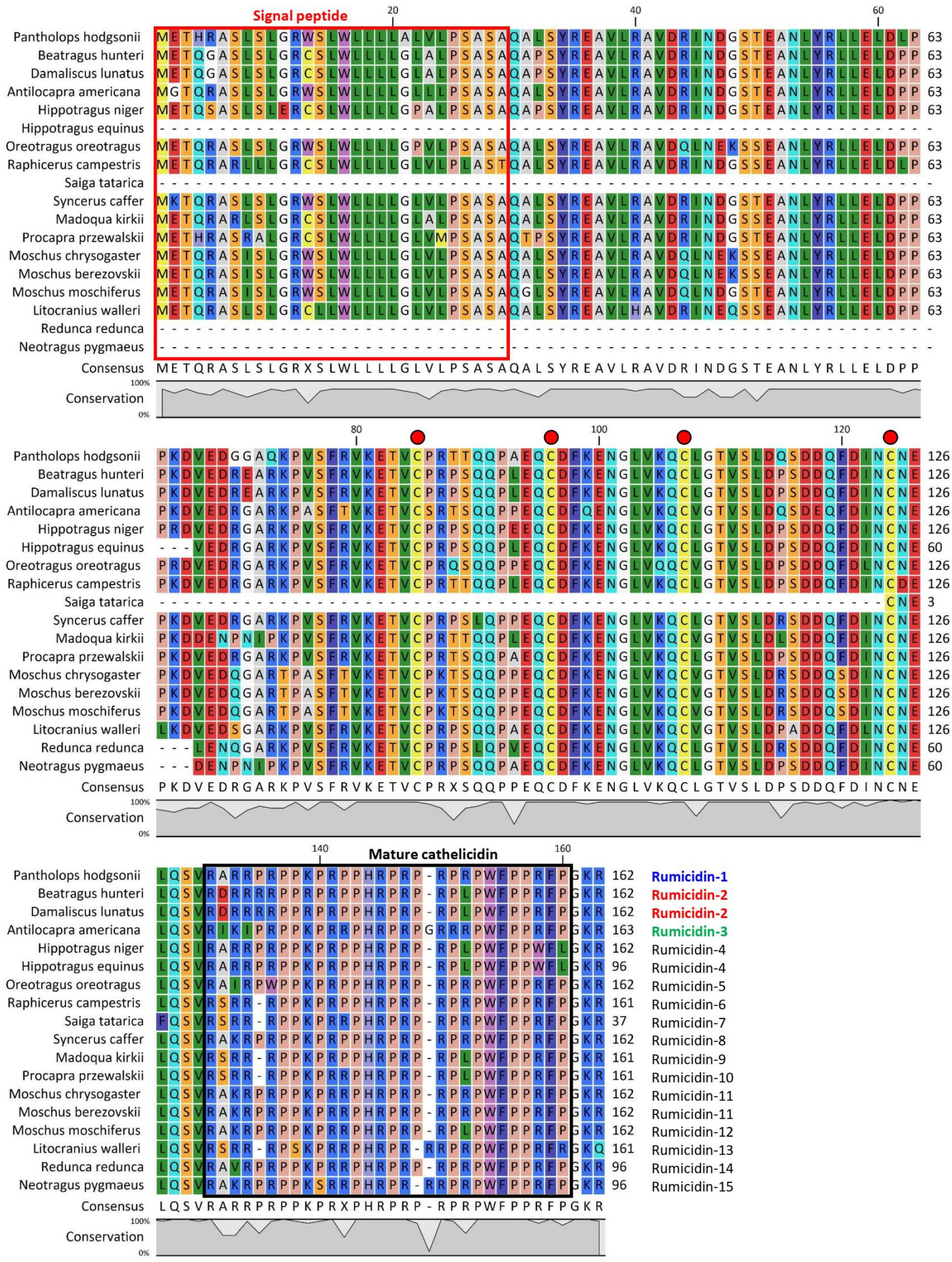

Supplementary Fig. 1. Multiple sequence alignment of preprorumicidins. Conservative cysteine residues are marked with red circles.

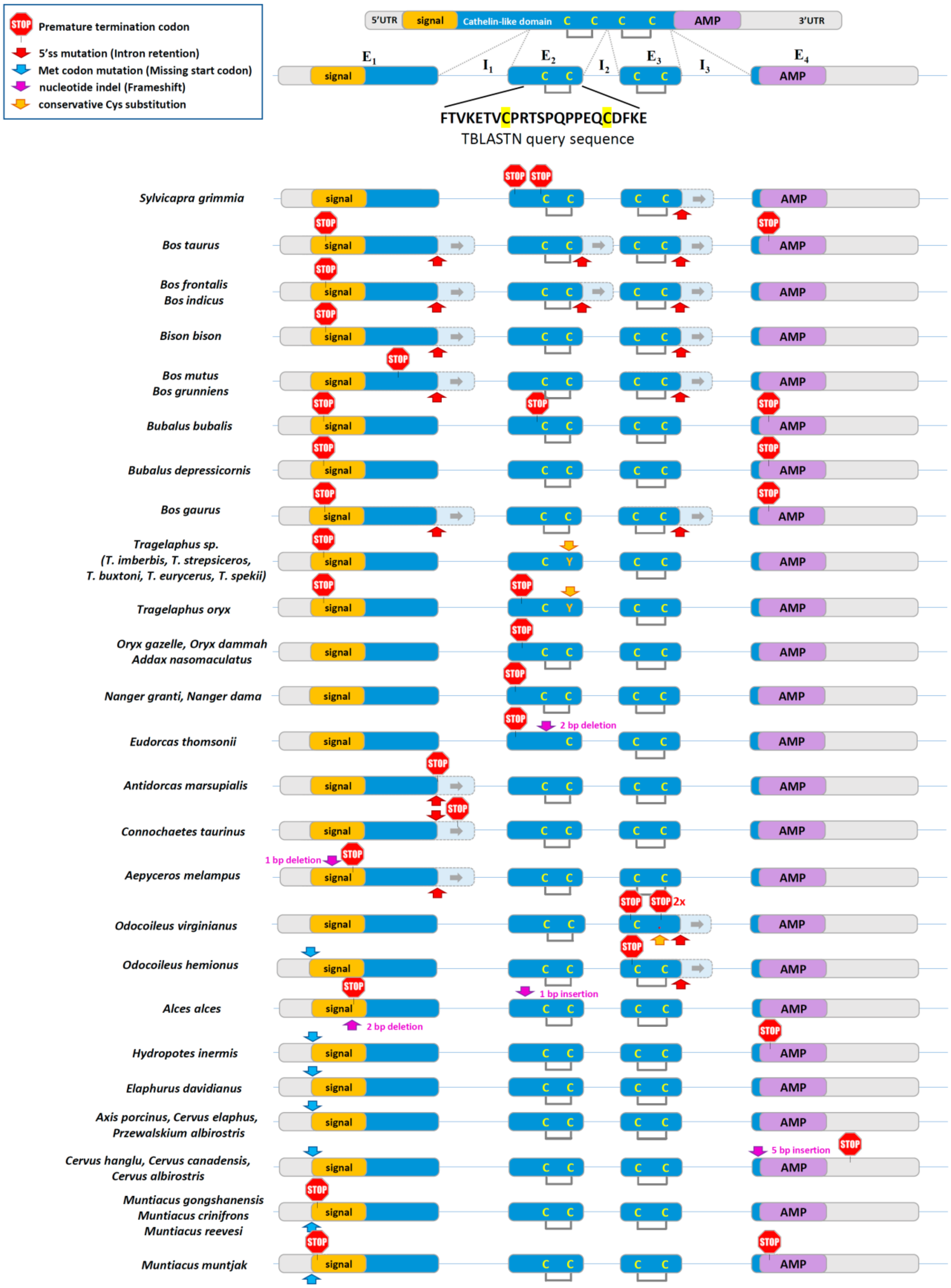

Supplementary Fig. 2. Pseudogenization map of *CATHL(3L2/8)* family.

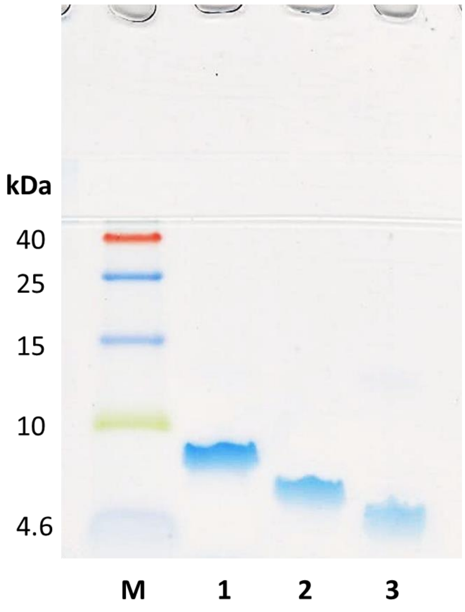

Supplementary Fig. 3. Tricine-SDS-PAGE in 16.5% gel containing 6 M urea. M – protein ladder (Spectra), 1 – recombinant rumicidin-1 (1 μg), 2 – recombinant rumicidin-1 analog [9-29] (1 μg), 3 – recombinant rumicidin-1 analog [15-29] (1 μg).

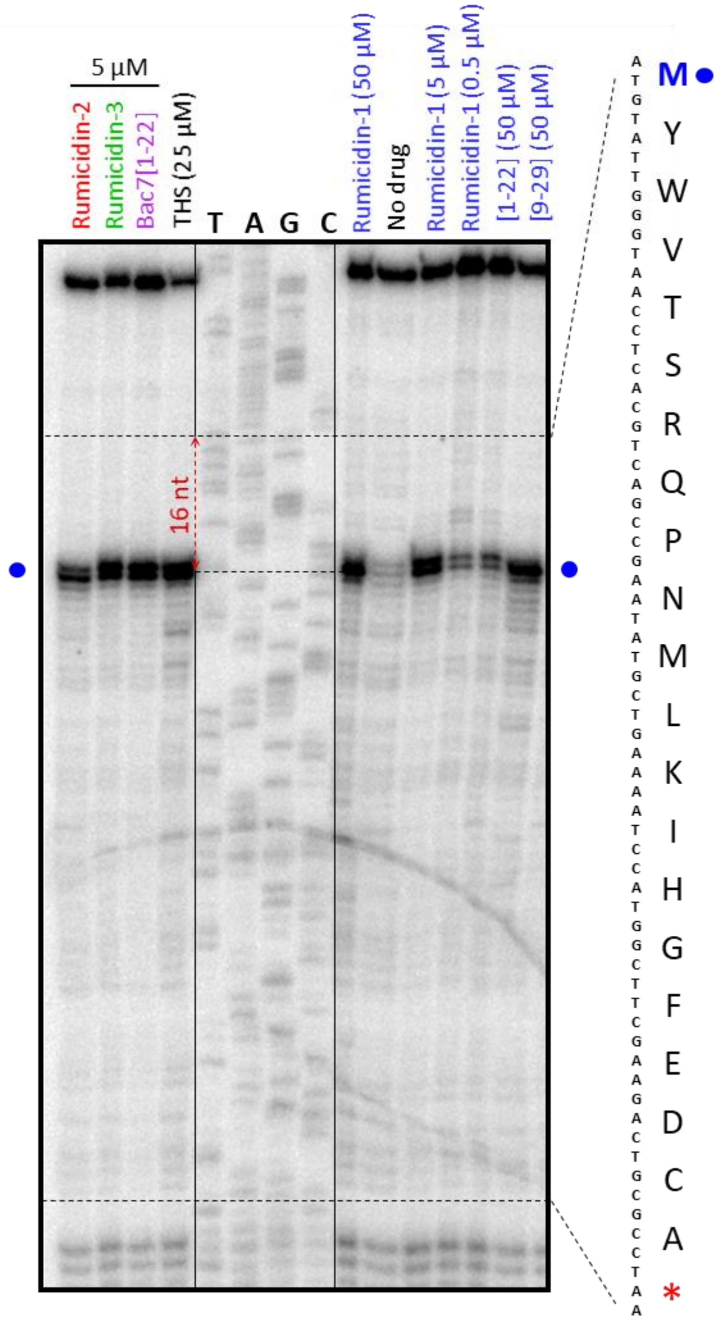

Supplementary Fig. 4. Ribosome stalling by rumicidins on RST1 mRNA, as revealed by reverse-transcription inhibition (toe-printing) assay in a cell-free translation system.

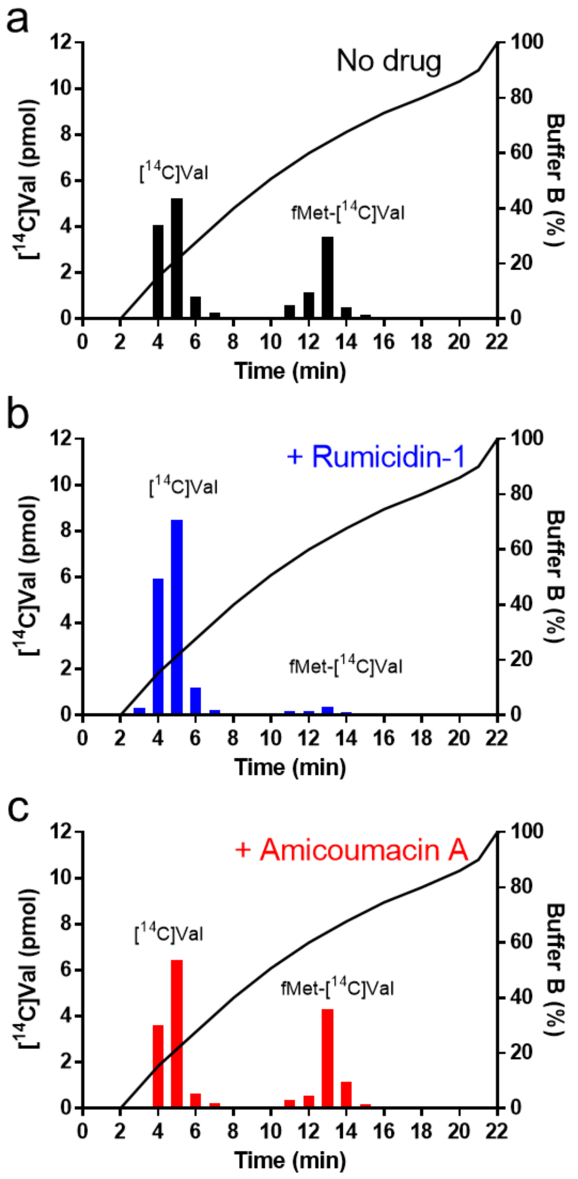

Supplementary Fig. 5. Rumicidin-1 inhibits dipeptide formation. Shown is the separation of [^{14}C]Val and fMet-[^{14}C]Val by reversed-phase HPLC upon interaction of 70S initiation complexes with ternary complexes EF-Tu·GTP·[^{14}C]Val-tRNA^{Val} in the absence of any drugs **(a)**, or in the presence of 1 μM rumicidin-1 **(b)**, or 30 μM amicoumacin A **(c)**.

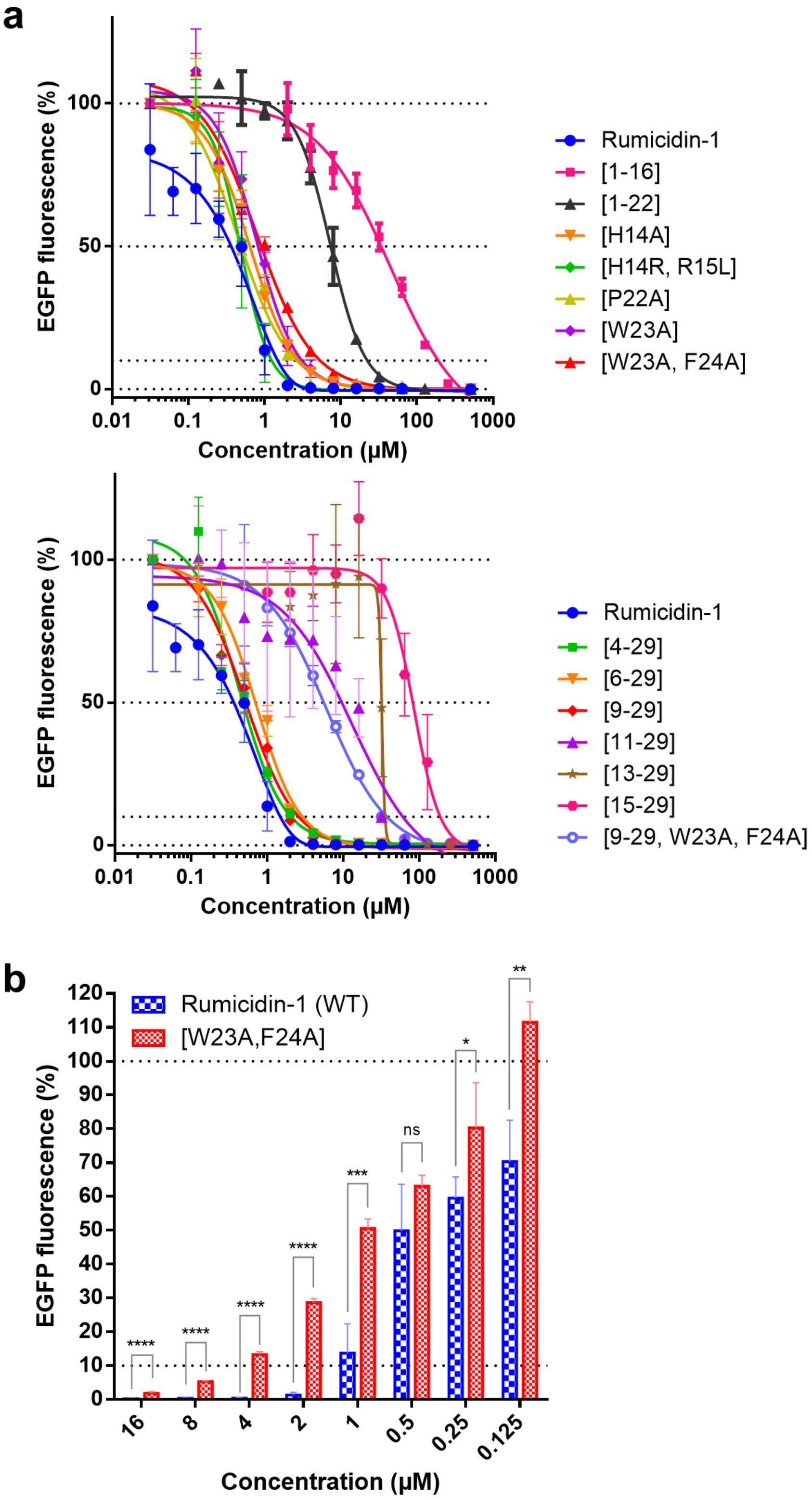

Supplementary Fig. 6. Effects of rumicidin-1 and its analogs at different concentrations on the fluorescence resulting from the *in vitro* coupled transcription/translation of EGFP with the use of *E. coli* BL21 (DE3) Star cell extract. Data are the mean ± SD of three independent experiments performed in triplicate. Student's t-test for each concentration of rumicidin-1 vs the analog [W23A, F24A] (panel "b"). Significance levels are: * $p < 0.05$, ** $p < 0.01$, *** $p < 0.001$, **** $p < 0.0001$, ns – not significant.

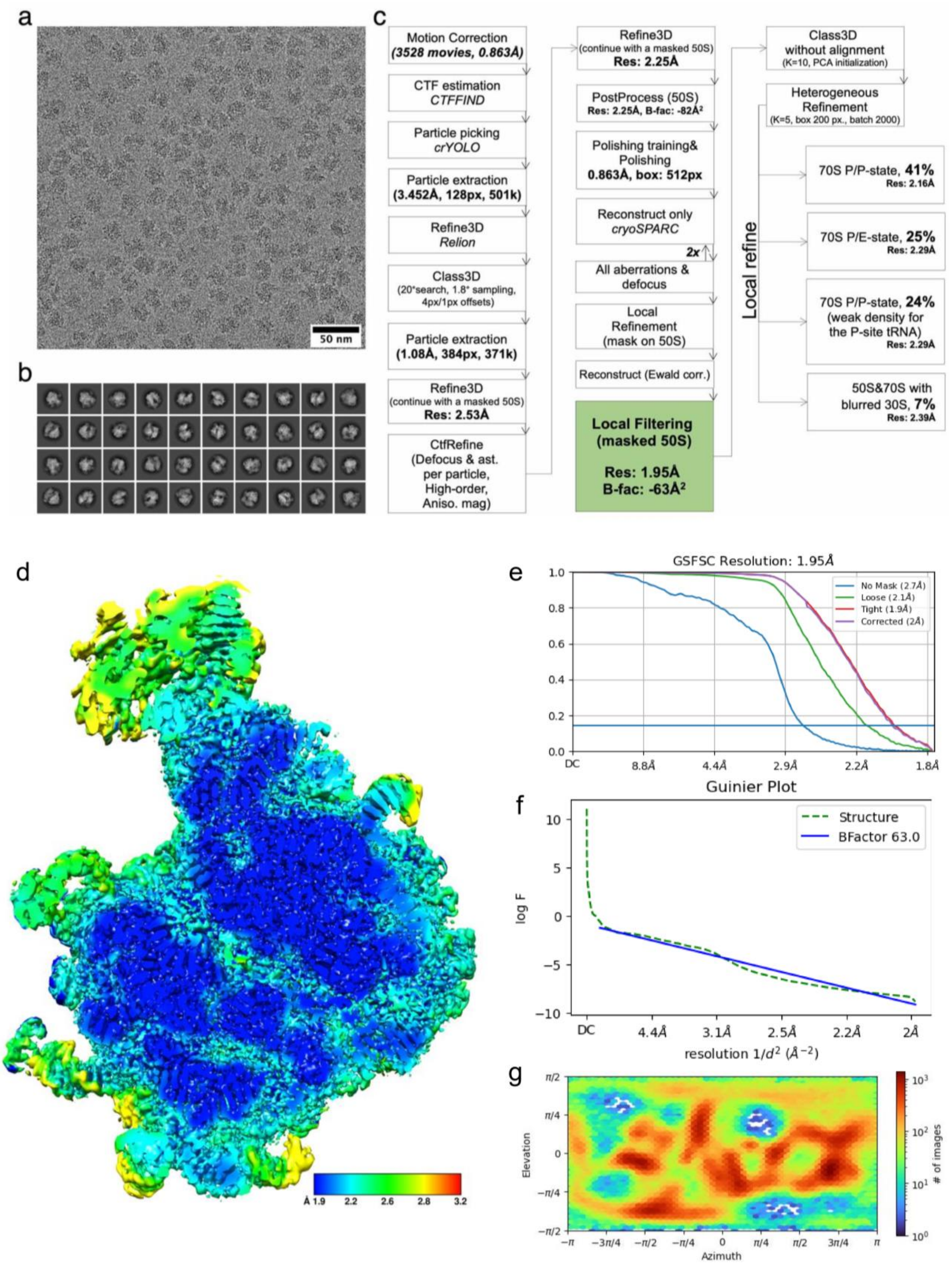

Supplementary Fig. 7. Cryo-EM data processing and resolution assessment of the cryo-EM structure of rumicidin-2 bound to the bacterial ribosome. **(a)** Representative cryo-EM image, scale bar is 50 nm. **(b)** 2D classes. **(c)** Data processing workflow. **(d)** Local resolution map of the 50S subunit after local refinement. **(e)** Fourier shell correlation (FSC=0.143) plot. **(f)** Guinier plot calculated in cryoSPARC. **(g)** Particles distribution map after local refinement.

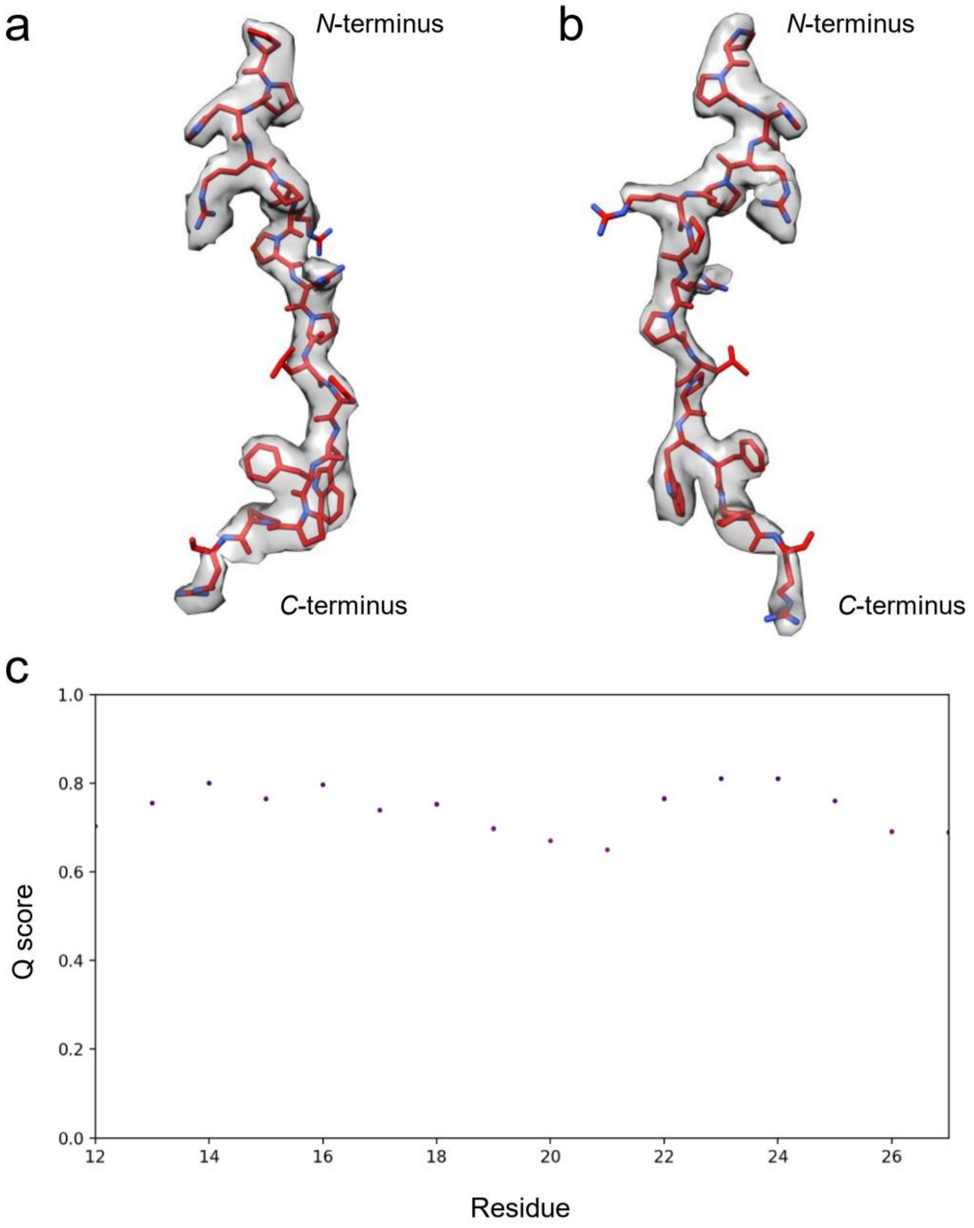

Supplementary Fig. 8. Additional ligand views. **(a-b)** Rotated views of the atomic model of rumicidin-2 superimposed on the corresponding unsharpened cryo-EM density map. **(c)** Q-score calculated for each modelled residue of rumicidin-2 at the same map level.

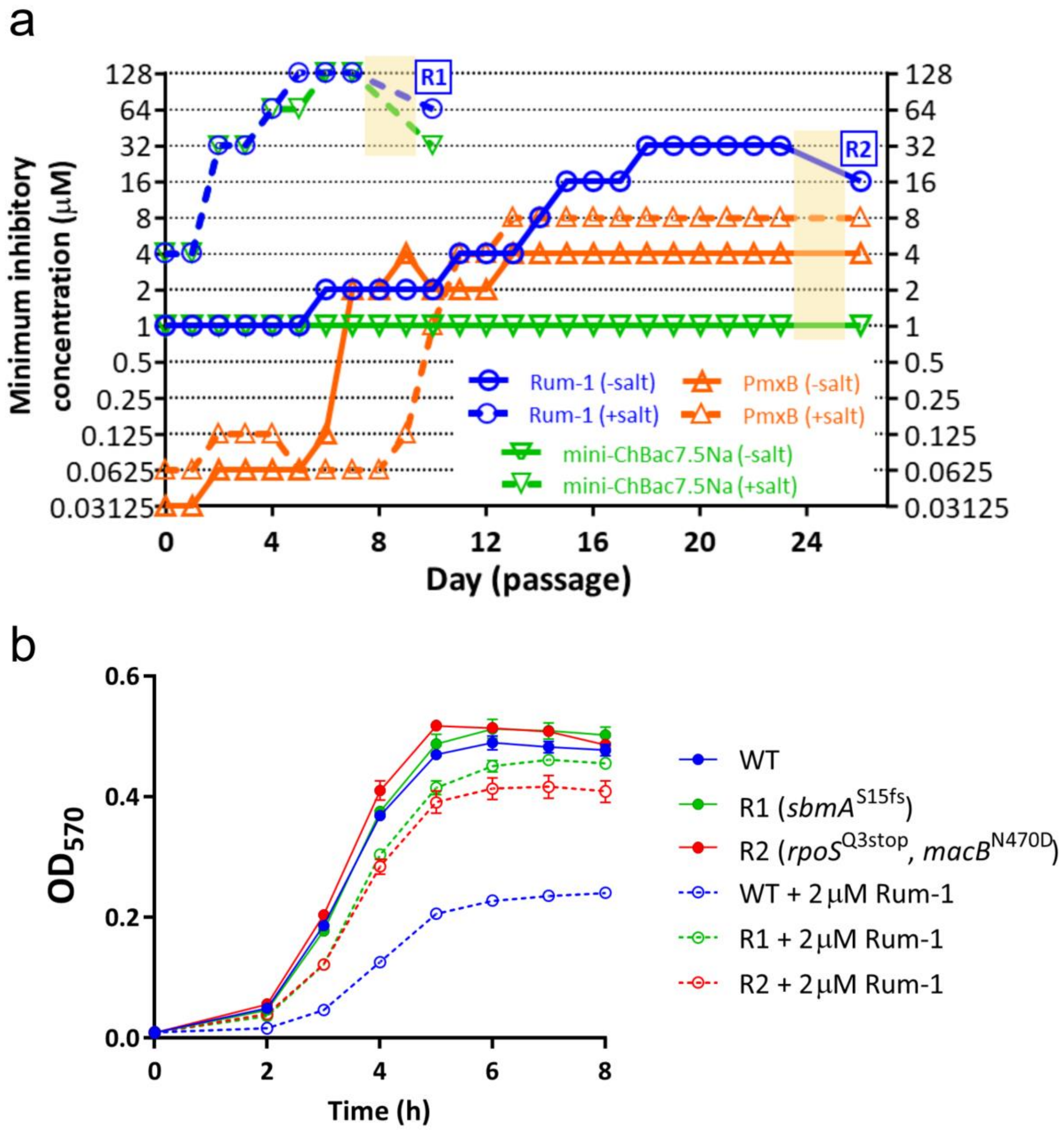

Supplementary Fig. 9. Bacterial resistance to rumicidin-1 **(a)** Serial passage resistance induction of PrAMPs and polymyxin B against the MDR *E. coli* 1057. Bacteria that grew at the highest concentration of AMPs after the final passage (on the 7th or 23rd day) were further passaged three times on drug-free agar plates before determining the final MIC value (yellow shading). No differences in MICs before and after 23 passages without antimicrobial agents were observed. The 128-fold increase in MIC values was registered while subjected to selection by polymyxin B (the control antibiotic) in both media (MHB ±0.9%NaCl). **(b)** Analysis of growth rates of wild-type *E. coli* 1057 (WT) and rumicidin-1-resistant strains (R1 and R2) in LB medium. Initial OD_{570} of bacterial cultures was 0.01. The experiment was performed in triplicate, with the plotted points representing the mean value ± SD.

Antimicrobial	Class	WGS-predicted phenotype	Genetic background
amikacin	aminoglycoside	No resistance	
tigecycline	tetracycline	No resistance	
tobramycin	aminoglycoside	No resistance	
cefepime	beta-lactam	Resistant	blaCTX-M-15 (blaCTX-M-15_AY044436)
chloramphenicol	amphenicol	No resistance	
piperacillin+tazobactam	beta-lactam	No resistance	
cefoxitin	beta-lactam	No resistance	
ampicillin	beta-lactam	Resistant	blaCTX-M-15 (blaCTX-M-15_AY044436)
ampicillin+clavulanic acid	beta-lactam	No resistance	
cefotaxime	beta-lactam	Resistant	blaCTX-M-15 (blaCTX-M-15_AY044436)
ciprofloxacin	quinolone	Resistant	gyrA (p.S83L)
colistin	polymyxin	No resistance	
sulfamethoxazole	folate pathway antagonist	Resistant	sul1 (sul1_U12338)
imipenem	beta-lactam	No resistance	
trimethoprim	folate pathway antagonist	No resistance	
nalidixic acid	quinolone	Resistant	gyrA (p.S83L), gyrA (p.D87N)
ertapenem	beta-lactam	No resistance	
tetracycline	tetracycline	No resistance	
fosfomycin	fosfomycin	No resistance	
ceftazidime	beta-lactam	Resistant	blaCTX-M-15 (blaCTX-M-15_AY044436)
temocillin	beta-lactam	No resistance	
gentamicin	aminoglycoside	No resistance	
meropenem	beta-lactam	No resistance	
azithromycin	macrolide	Resistant	mph(A) (mph(A)_D16251)

Supplementary Fig. 10. Identification of acquired antibiotic resistance genes in *E. coli* 1057 strain. The analysis was performed using ResFinder 4.1 (https://cge.food.dtu.dk/services/ResFinder/).

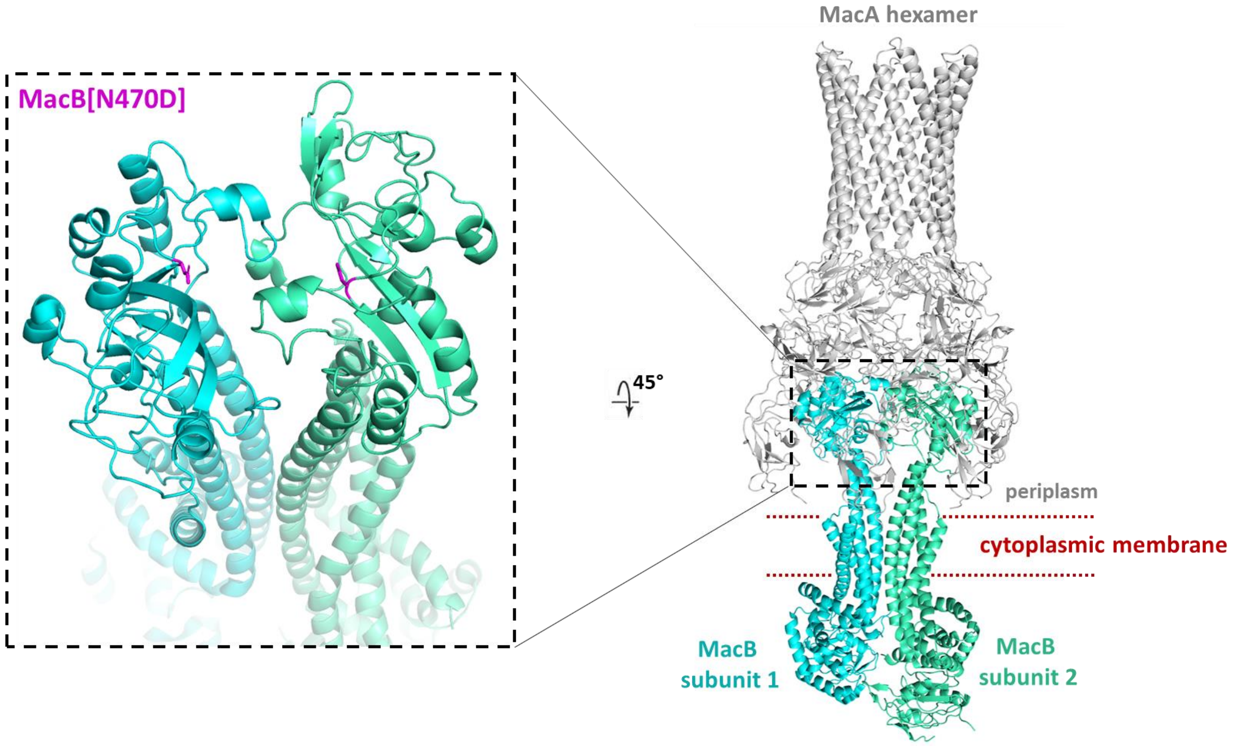

Supplementary Fig. 11. The localization of amino acid substitution MacB[N470D] found in rumicidin-1-resistant *E. coli* strain presented in the model of the MacAB-TolC pump [PDB 5NIL]. The model was visualized with PyMOL software.

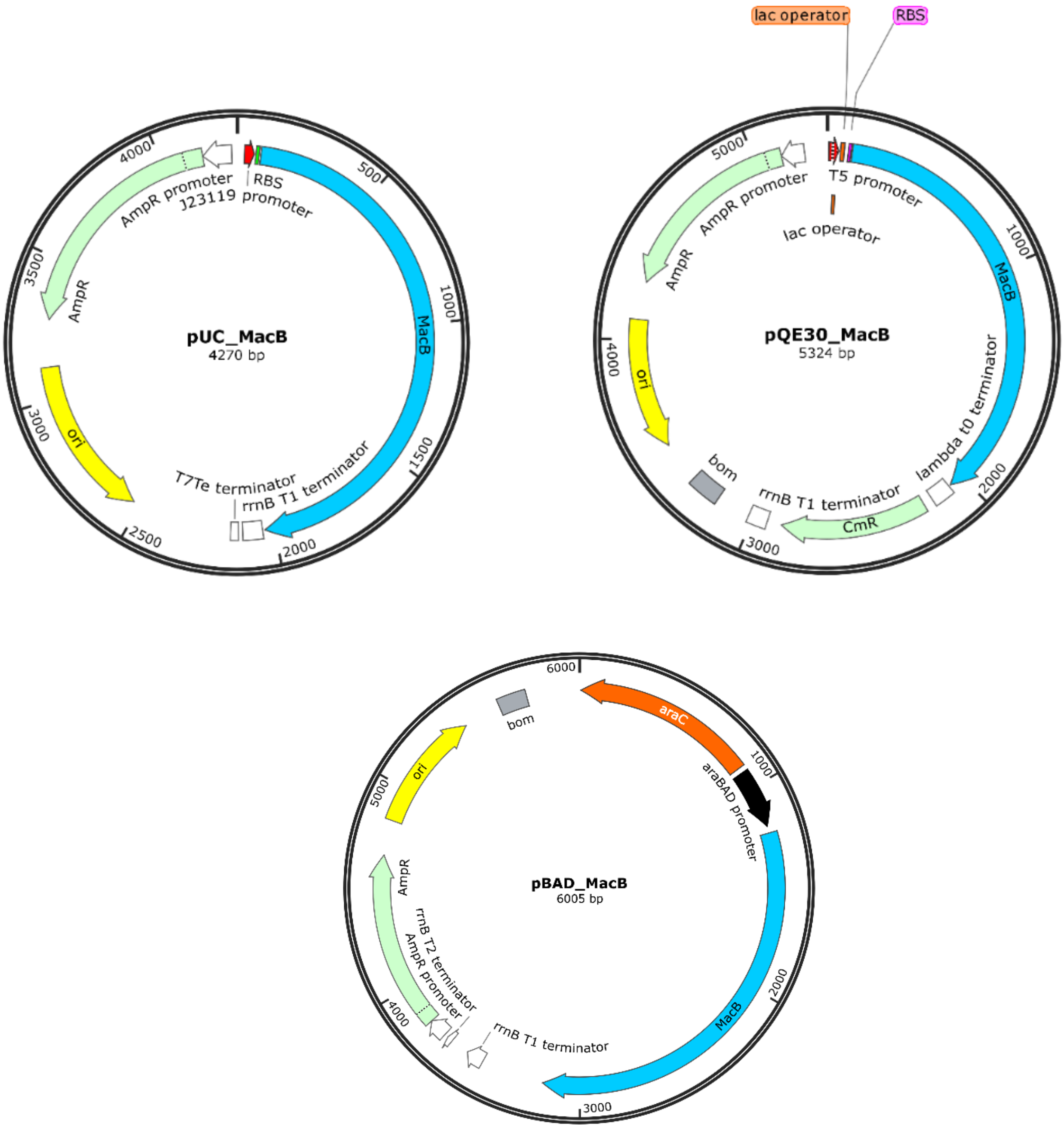

Supplementary Fig. 12. Maps of complementation plasmids. Maps were visualized with SnapGene software.

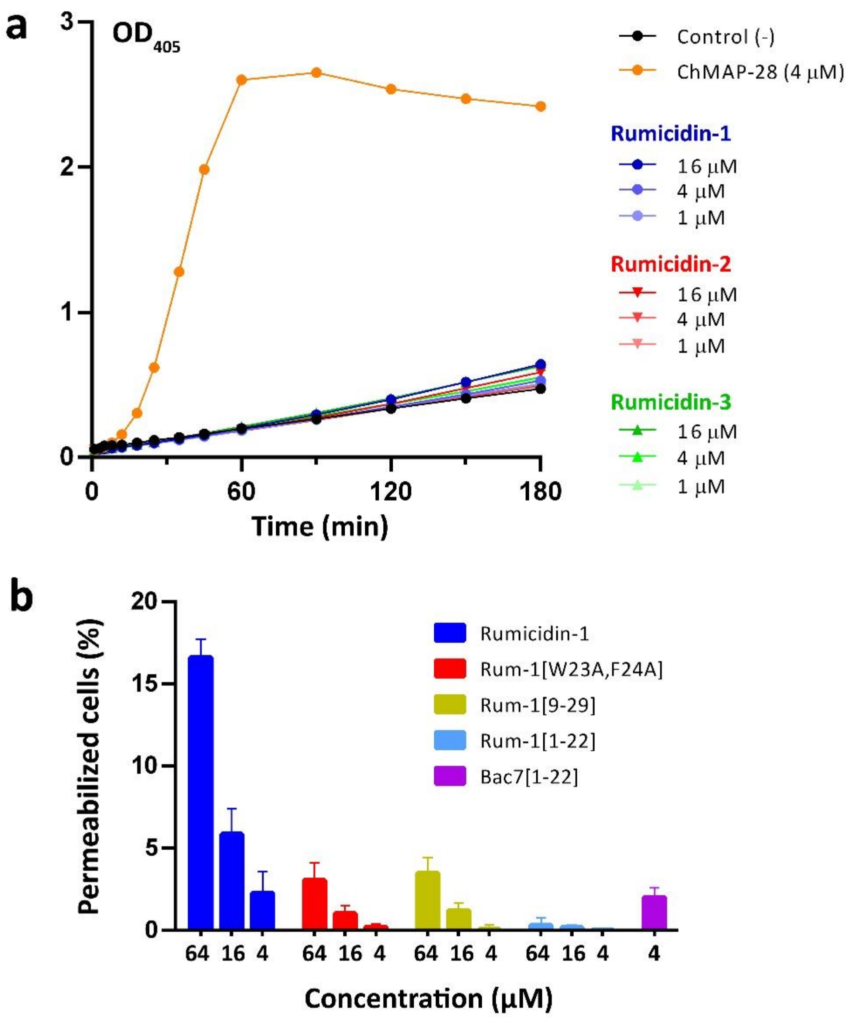

Supplementary Fig. 13. Cytoplasmic membrane permeability assay. **(a)** Kinetics of changes in of *E. coli* ML-35p cytoplasmic (inner) membrane permeability measured with the use of chromogenic marker – the product of ONPG (OD_{405}) hydrolysis. The effects of rumicidins on bacterial membranes were analyzed with the use of the *E. coli* ML-35p strain lacking the functional lactose permease (which is necessary for the uptake of ONPG) and constitutively expressing β-galactosidase that hydrolyze it to o-nitrophenol. ChMAP-28, known as a potent α-helical cytolytic cathelicidin peptide, was used as a positive control[1]. **(b)** The ONPG testing for permeability of the cytoplasmic membrane of *E. coli* ML-35p. The graph shows effects of rumicidin-1 and its analogs at different concentrations after 4 h exposure. Data are the mean ± SD of two independent experiments. The optical absorption of the solution after incubation with melittin for 4 h was taken as 100%. The absorbance of control wells without peptides was subtracted from the absorbance value of each well. Experiments were performed in 10 mM sodium phosphate buffer (NaPB, pH 7.4) with 0.9% NaCl. Source data are provided as a Source Data file.

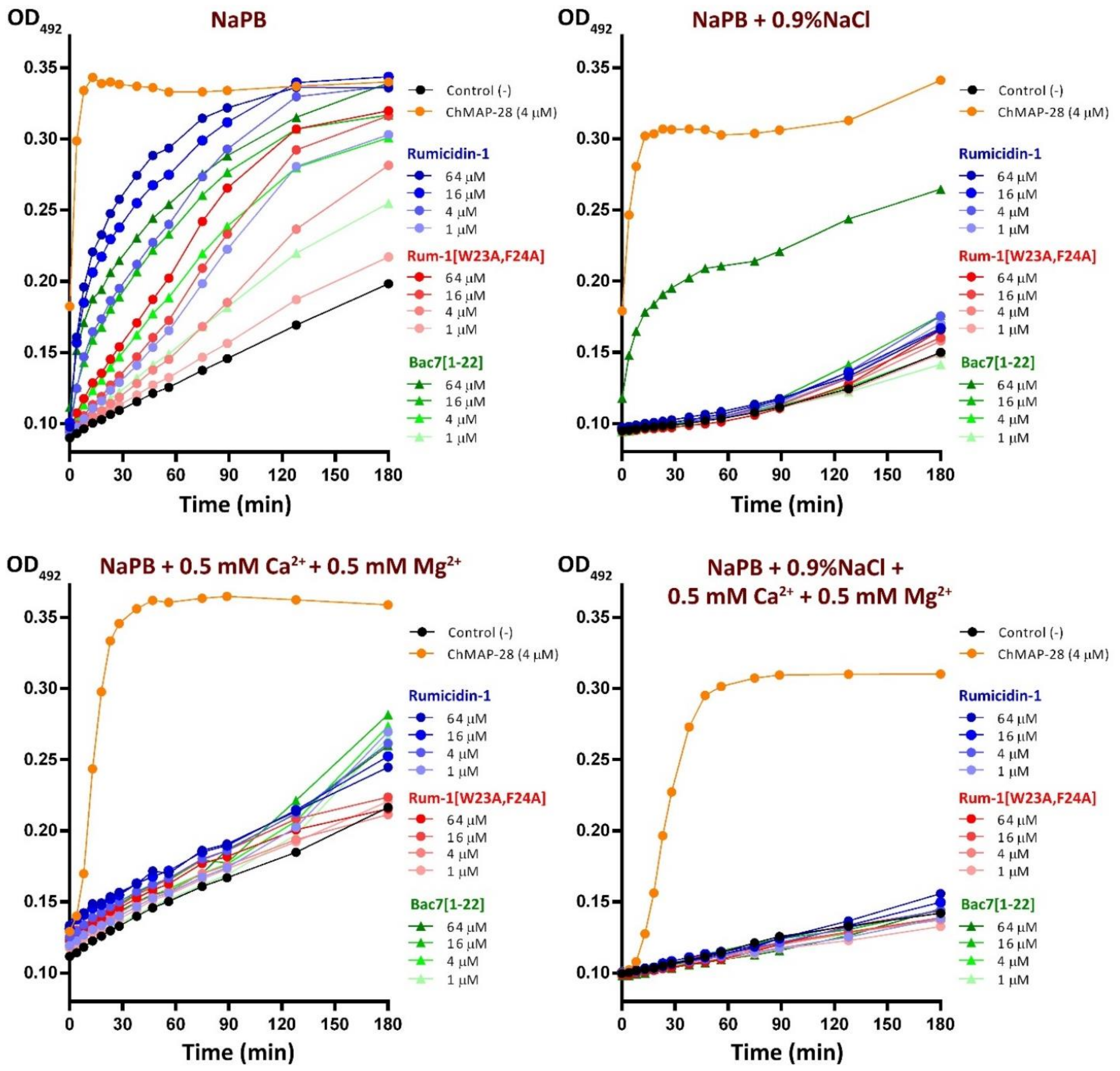

Supplementary Fig. 14. Kinetics of changes in bacterial outer membrane permeability measured with the use of chromogenic marker – the product of nitrocefin (OD_{492}) hydrolysis. The effects of rumicidin-1, its analog [W23A,F24A], and Bac7[1-22] on the outer membrane were analyzed with the use of the *E. coli* ML-35p strain which constitutively synthesizes β-lactamase in periplasmic space that can hydrolyze nitrocefin. ChMAP-28, known as a potent α-helical cytolytic cathelicidin peptide[1], was used as a positive control. Experiments were performed in 10 mM sodium phosphate buffer (NaPB, pH 7.4) with or without the addition of 0.9% NaCl and/or a mixture of divalent cations (0.5 mM $CaCl_2$ and 0.5 mM $MgSO_4$). Two independent experiments were performed, and the curve pattern was the same. Source data are provided as a Source Data file.

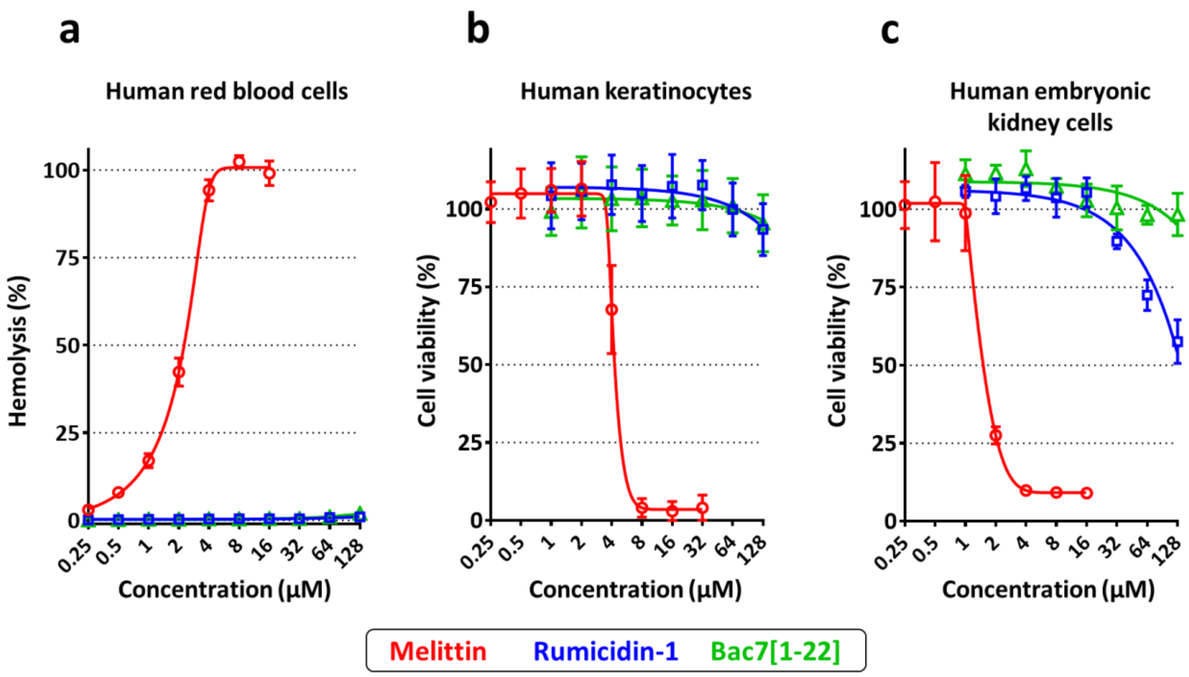

Supplementary Fig. 15. Biological activity of rumicidin-1 against mammalian cells. **(a)** Hemolytic activity (hemoglobin release assay) and cytotoxicity (MTT-assay) towards human adherent **(b)** HaCaT (keratinocytes) and **(c)** HEK293T (transformed embryonic kidney cells) cells of rumicidin-1, Bac7[1-22], and melittin. Melittin known as potent cytotoxic agent completely lysed any cells tested at concentrations of ≥4-8 µM. Data are the mean ± SD of two independent experiments performed in triplicate.

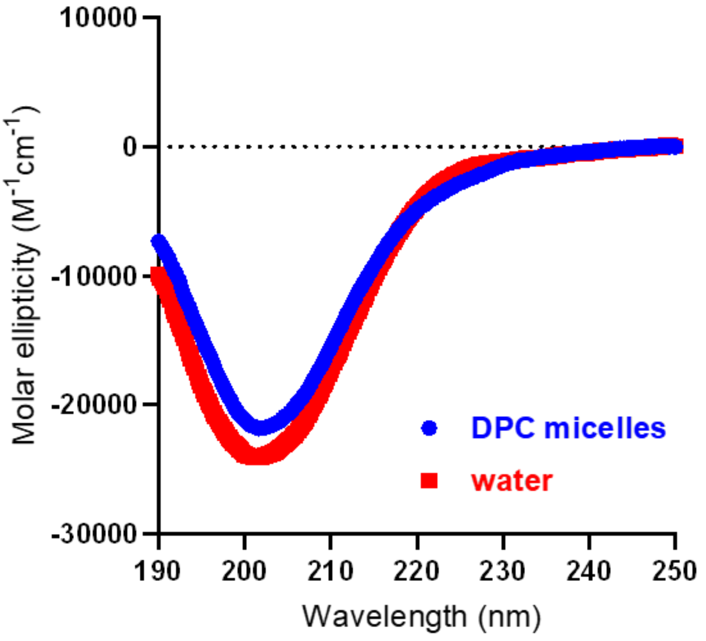

Supplementary Fig. 16. The circular dichroism (CD) spectra of rumicidin-1 in different environments measured at 25°C on a Jasco J-810 instrument (Jasco) in water solution and 30 mM dodecylphosphocholine (DPC) micelles (Avanti Polar Lipids) mimicking a neutral mammalian membrane. The final concentration of the peptide was of 300 µM. Four consecutive scans were performed and averaged, followed by subtraction of the blank spectrum of the solvent. Source data are provided as a Source Data file.

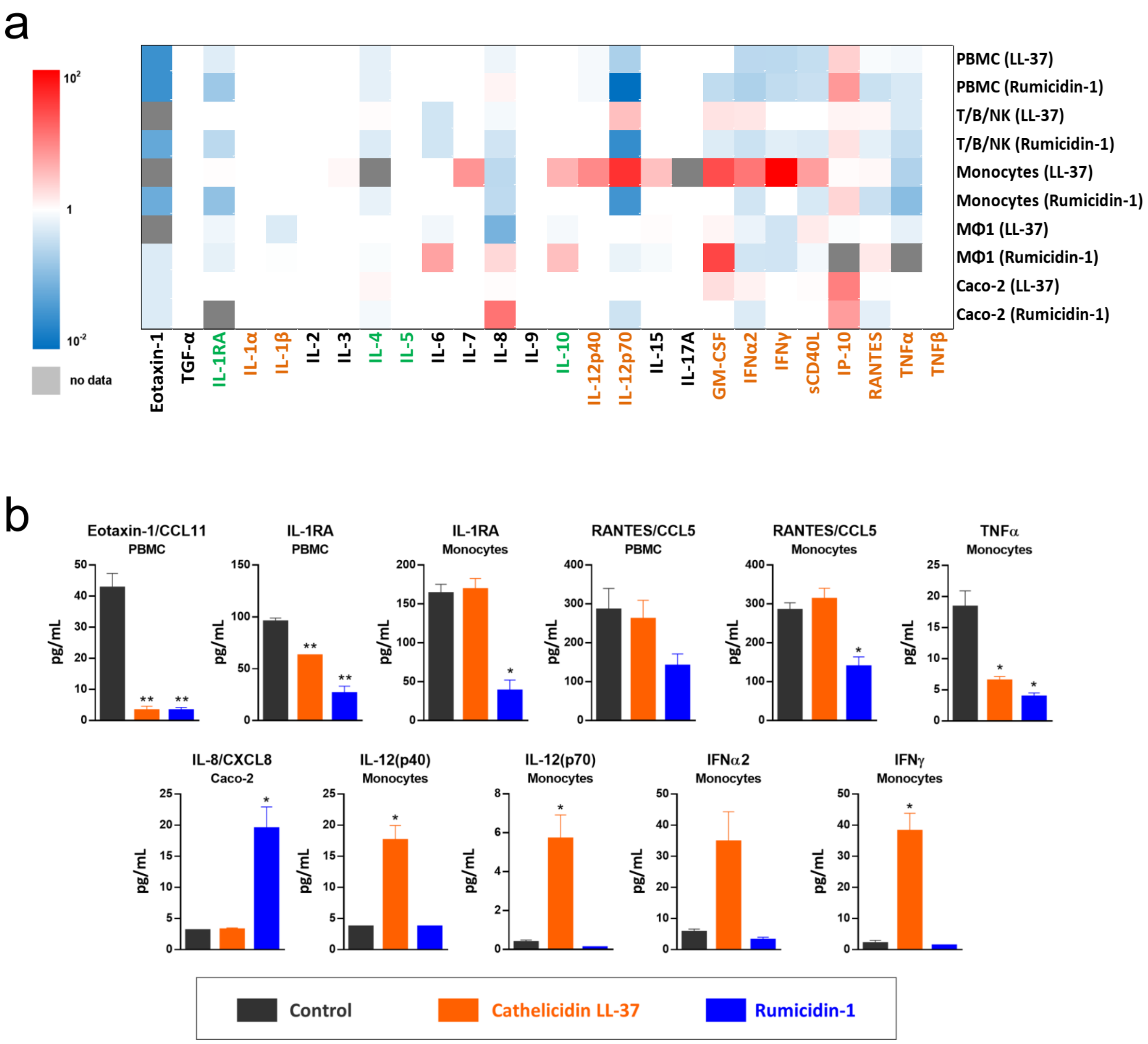

Supplementary Fig. 17. Immunomodulatory activities of cathelicidins. **(a)** Heat map represented profiles of cytokines/chemokines/growth factors production by different human cell lines in response to incubation with rumicidin-1 (2 μM) and human cathelicidin LL-37 (2 μM) related to the control without AMP treatment. The initial data on concentrations of cytokines/chemokines/growth factors obtained by a bead-based multiplex immunoassay are presented in Supplementary Table 8. Proinflammatory factors are marked with orange, anti-inflammatory factors are marked with green. **(b)** Absolute levels of cytokines and chemokines production by different cell cultures *in vitro*. Error bars represent standard deviation (±SD) between two biological replications. Significance levels are: * $p < 0.05$, ** $p < 0.01$.

Supplementary Table 1. Transcriptome assembly and identification of rumicidins

SRA run ID	Species	Identified AMP sequence
SRR5642347	*Damaliscus pygargus*	RDRRRRPPRPRPPHRPRPRPLPWFPPRFP GKR (Rumicidin-2)
SRR26378580	*Antilocapra americana*	RIKIPRPPKPRRPHRPRPGRRRPWFPPRFP GKR (Rumicidin-3)
SRR5642370	*Madoqua kirkii*	RSRRRPPKPRPPHRPRPRPLPWFPPRFP GKR (Rumicidin-9)
SRR10204413	*Moschus berezovskii*	RAKRPRPPKPRRPHRPRPRPRPWFPPRFP GKR (Rumicidin-11)

The raw data for all samples was obtained from NCBI Sequence Read Archive (SRA). The data was converted to fastq format with fasq-dump utility of sra-toolkit and then the quality of reads was assessed using fastqc. The raw reads for each sample were filtered to remove low quality reads and adapter sequences using Trimmomatic software (v.0.38) with following parameters: ILLUMINACLIP:2:30:10, LEADING:30, TRAILING:30, SLIDINGWINDOW:10:25. The trimmed reads were then filtered from ribosomal RNA by aligning them to the SILVA database (release 138.1) using bowtie2 (v.2.3.5.1). Unaligned reads were used to assemble transcriptome with Trinity pipeline (v.2.9.1) with a minimum contig length of 200 bp. Assembled transcriptomes were then directly translated and converted into protein blast databases. The target cathelicidin protein sequences were aligned against obtained databases with blastp, 100% query cover indicated presence of cathelicidin RNA in the transcriptome and thus possible expression *in vivo*.

Supplementary Table 2. Amino acid sequences and molecular masses of the peptides used in this study

Peptide	Origin	Sequence	Molecular mass, Da	
			Calculated $[M+H]^+$ value*	Measured value**
Bac7[1-22]	Rec	RRIRPRPPRLPRPRPRPLPFPR	2783.72	2783.89
PR-39[1-22]	Rec	RRRPRPPYLPRPRPPPFFPPRL	2765.61	2765.35
mini-ChBac7.5Nα	Rec	RRLRPRRPRLPRPRPRPRPRPR	2894.81	2894.05
Rumicidin-1	Rec	RARRPRPPKPRPPHRPRPRPRPWFPPRFP	3658.12	3658.01
Rum-1[1-16]	Rec	RARRPRPPKPRPPHRP	1971.18	1971.28
Rum-1[1-22]	Rec	RARRPRPPKPRPPHRPRPRPRP	2730.64	2730.58
Rum-1[4-29]	Rec	RPRPPKPRPPHRPRPRPRPWFPPRFP	3274.88	3274.70
Rum-1[6-29]	Rec	RPPKPRPPHRPRPRPRPWFPPRFP	3021.72	3021.51
Rum-1[9-29]	Rec	KPRPPHRPRPRPRPWFPPRFP	2671.52	2670.70
Rum-1[9-29, W23A, F24A]	Rec	KPRPPHRPRPRPRP**AA**PPRFP	2480.44	2480.24
Rum-1[11-29]	Rec	RPPHRPRPRPRPWFPPRFP	2446.37	2446.23
Rum-1[13-29]	Rec	PHRPRPRPRPWFPPRFP	2193.21	2193.12
Rum-1[15-29]	Rec	RPRPRPRPWFPPRFP	1959.10	1959.23
Rum-1[H14A]	Rec	RARRPRPPKPRPP**A**RPRPRPRPWFPPRFP	3592.09	3591.88
Rum-1[P22A]	Rec	RARRPRPPKPRPPHRPRPRPR**A**WFPPRFP	3632.10	3632.08
Rum-1[W23A]	Rec	RARRPRPPKPRPPHRPRPRPRP**A**FPPRFP	3543.07	3542.92
Rum-1[W23A, F24A]	Rec	RARRPRPPKPRPPHRPRPRPRP**AA**PPRFP	3467.04	3467.11
Rum-1[H14R, R15L]	Rec	RARRPRPPKPRPP**RL**PRPRPRPWFPPRFP	3634.14	3634.68
Rumicidin-2	Rec	RDRRRRPPRPRPPHRPRPRPLPWFPPRFP	3746.14	3746.57
Rumicidin-3	Rec	RIKIPRPPKPRRPHRPRPGRRRPWFPPRFP	3804.26	3804.81
Melittin	Synt	GIGAVLKVLTTGLPALISWIKRKRQQ	2846.74	2846.60
LL-37	Synt	LLGDFFRKSKEKIGKEFKRIVQRIKDFLRNLVPRTES	4491.58	4491.40
VicBac	Synt	RRIRRPRLPRPRVPRPRIPPRIPRPVLPPPRVPFPRFPR	4815.98	4815.70
ChMAP-28	Rec	GRFKRFRKKLKRLWHKVGPFVGPILHY	3364.00	3364.22
Thanatin	Rec	GSKKPVPIIYCNRRTGKCQRL	2415.32	2415.36
Tachyplesin-1	Rec	KWCFRVCYRGICYRRCR	2264.08	2263.73
Protegrin-1	Rec	RGGRLCYCRRRFCVCVGR	2156.07	2156.01

* according to Expasy Isotopident tool

** monoisotopic m/z were measured using MALDI-TOF MS (the measured m/z values match well the corresponding calculated molecular masses)

Supplementary Table 3. Cryo-EM data collection and model refinement statistics

Complexes	***E. coli*** **70S ribosome in complex with PrAMP rumicidin-2**
Cryo-EM data	
Microscope	FEI Titan Krios
Accelerating Voltage, kV	300
Detector	Falcon II
Spherical aberration, mm	<0.1
Magnification	75,000x
Defocus range, µm	-0.5 to -1.8
Number of frames	32
Total dose, $e^{-}/Å^{2}$	80
Micrographs	3528
Cryo-EM reconstruction	
Particles picked	501,000
Particles refined	371,856
Resolution achieved, Å	1.95 Å
Refinement (50S subunit)	
No. of Non-Hydrogen Atoms	
All atoms	90,773
Protein residues	3,129
Nucleotides	2,847
Ramachandran Plot	
Favored regions, %	97.10
Allowed regions, %	2.81
Outliers, %	0.1
Deviations from ideal values (RMSD)	
Bond, Å	0.008
Angle, degrees	1.422

Supplementary Table 4. Mutations identified in *E. coli* 1057 strains with stable induced resistance to PrAMPs

Strain	Selection conditions	Mutation	Gene	Type	Gene product
E. coli 1057 (control)	MHB* (23 passages)	R117C (**C**GC→**T**GC)	*thiP*	single nucleotide polymorphism	thiamin ABC transporter membrane subunit
Rum-1-res *E. coli* 1057 (R1)	MHB + 0.9%NaCl Rumicidin-1 (7 passages)	Δ2 bp after nucleotide 42 (CTC **XX**G GCC)	*sbmA*	frameshift	cytoplasmic membrane transporter
Rum-1-res *E. coli* 1057 (R2)	MHB Rumicidin-1 (23 passages)	N470D (**A**AC→**G**AC)	*macB*	single nucleotide polymorphism	ABC transporter (part of tripartite efflux pump)
		Q3stop (**C**AG→**T**AG)	*rpoS*	in-frame stop codon	RNA polymerase sigma factor σ^S

*MHB - Mueller-Hinton broth

Supplementary Table 5. Antibacterial activities of rumicidins against the *Mycobacterium smegmatis* mc(2)155

**Test medium**	**Minimum inhibitory concentration (MIC, µM) ***			
	Rumicidin-1	Rumicidin-2	Rumicidin-3	PR-39[1-22]
LB	0.25	1	4	8
LB + 10% FBS	0.5	n.d.	n.d.	16
Muller-Hinton broth + 0.9% NaCl	0.25	1	4	16
Middlebrook 7H9	0.125	n.d.	n.d.	n.d.

* - MICs are expressed as the median values determined based on at least three independent experiments performed in triplicate

Supplementary Table 6. Absolute levels of 26 cytokines, chemokines and growth factors assessed by multiplex xMAP technology.

Analyte	Eotaxin	TGF-a	GM-CSF	IFNa2	IFNg	IL-10	IL-12p40	IL-12p70	IL-15	sCD40L	IL-17A	IL-1RA	IL-1a
Sample	pg/ml	pg/ml	pg/ml	pg/ml	pg/ml	pg/ml	pg/ml	pg/ml	pg/ml	pg/ml	pg/ml	pg/ml	pg/ml
PBMC_control	42,69	<2.64↓	0,66	9,14	3,2	<1.47↓	4,16	0,88	<2.08↓	6,18	<0.94↓	95,52	<1.13↓
PBMC_LL37	3,21	<2.64↓	0,66	3,72	<1.31↓	<1.47↓	<3.69↓	0,29	<2.08↓	2,84	<0.94↓	62,83	<1.13↓
PBMC_Rumicidin-1	3,21	<2.64↓	<0.29↓	3,11	1,42	<1.47↓	<3.69↓	<0.03↓	<2.08↓	3,03	<0.94↓	26,39	<1.13↓
T/B/NK_control	n.d.	<2.64↓	0,45	4,99	1,93	<1.47↓	<3.69↓	0,44	<2.08↓	3,22	<0.94↓	6,49	<1.13↓
T/B/NK_LL37	n.d.	<2.64↓	0,66	7,02	1,93	<1.47↓	<3.69↓	1,03	<2.08↓	3,22	<0.94↓	6,49	<1.13↓
T/B/NK_Rumicidin-1	<2.04↓	<2.64↓	<0.29↓	2,52	<1.31↓	<1.47↓	<3.69↓	<0.03↓	<2.08↓	<2.03↓	<0.94↓	<2.66↓	<1.13↓
Monocytes_control	22,37	<2.64↓	<0.29↓	5,66	<1.31↓	<1.47↓	<3.69↓	0,37	<2.08↓	5,73	<0.94↓	163,77	<1.13↓
Monocytes_LL37	n.d.	<2.64↓	3,08	34,74	38,2	4,1	17,59	5,7	4,8	20,8	n.d.	168,96	<1.13↓
Monocytes_Rumicidin-1	3,21	<2.64↓	<0.29↓	3,11	<1.31↓	<1.47↓	<3.69↓	<0.03↓	<2.08↓	3,41	<0.94↓	38,43	<1.13↓
MΦ1_control	3,21	<2.64↓	1,15	5,66	2,44	6,97	<3.69↓	0,58	2,32	2,47	<0.94↓	25,21	<1.13↓
MΦ1_LL37	n.d.	<2.64↓	1,32	4,35	<1.31↓	6,13	<3.69↓	0,58	2,42	3,22	<0.94↓	20,52	<1.13↓
MΦ1_Rumicidin-1	<2.04↓	<2.64↓	13,47	3,11	<1.31↓	16,39	<3.69↓	0,44	<2.08↓	2,12	<0.94↓	18,22	<1.13↓
Caco-2_control	3,21	<2.64↓	<0.29↓	3,11	<1.31↓	<1.47↓	<3.69↓	0,29	<2.08↓	<2.03↓	<0.94↓	<2.66↓	<1.13↓
Caco-2_LL37	<2.04↓	<2.64↓	0,45	3,72	<1.31↓	<1.47↓	<3.69↓	0,29	<2.08↓	2,12	<0.94↓	<2.66↓	<1.13↓
Caco-2_Rumicidin-1	<2.04↓	<2.64↓	<0.29↓	1,97	<1.31↓	<1.47↓	<3.69↓	0,15	<2.08↓	<2.03↓	<0.94↓	n.d.	<1.13↓

Analyte	IL-9	IL-1b	IL-2	IL-3	IL-4	IL-5	IL-6	IL-7	IL-8	IP-10	RANTE	TNFa	TNFb
Sample	pg/ml	pg/ml	pg/ml	pg/ml	pg/ml	pg/ml	pg/ml	pg/ml	pg/ml	pg/ml	pg/ml	pg/ml	pg/ml
PBMC_control	<3.08↓	<2.88↓	<2.73↓	<3.02↓	54,23	<2.74↓	<2.42↓	<2.06↓	1975	1188	286,63	18,47	<2.85↓
PBMC_LL37	<3.08↓	<2.88↓	<2.73↓	<3.02↓	38,61	<2.74↓	<2.42↓	<2.06↓	1591	2236	262,49	15,91	<2.85↓
PBMC_Rumicidin-1	<3.08↓	<2.88↓	<2.73↓	<3.02↓	38,61	<2.74↓	<2.42↓	<2.06↓	2286	4640	141,82	11,12	<2.85↓
T/B/NK_control	<3.08↓	<2.88↓	<2.73↓	<3.02↓	48,94	<2.74↓	4,46	<2.06↓	472,69	103,33	782,15	8,68	<2.85↓
T/B/NK_LL37	<3.08↓	<2.88↓	<2.73↓	<3.02↓	51,58	<2.74↓	<2.42↓	<2.06↓	411,17	120,56	889,61	5,23	<2.85↓
T/B/NK_Rumicidin-1	<3.08↓	<2.88↓	<2.73↓	<3.02↓	31,17	<2.74↓	<2.42↓	<2.06↓	255,17	152,89	550,82	3,94	<2.85↓
Monocytes_control	<3.08↓	<2.88↓	<2.73↓	<3.02↓	37,35	<2.74↓	<2.42↓	<2.06↓	1813	845,2	284,28	18,32	<2.85↓
Monocytes_LL37	<3.08↓	<2.88↓	<2.73↓	3,38	n.d.	<2.74↓	<2.42↓	8,54	755,68	897,33	312,51	6,47	<2.85↓
Monocytes_Rumicidin-1	<3.08↓	<2.88↓	<2.73↓	<3.02↓	27,57	<2.74↓	<2.42↓	<2.06↓	770,49	1475	139,39	3,88	<2.85↓
MΦ1_control	<3.08↓	17,03	<2.73↓	<3.02↓	38,61	<2.74↓	2,87	<2.06↓	7174	299,89	710,7	12,94	<2.85↓
MΦ1_LL37	<3.08↓	10,63	<2.73↓	<3.02↓	38,61	<2.74↓	<2.42↓	<2.06↓	1188	282,01	715,62	9,49	<2.85↓
MΦ1_Rumicidin-1	<3.08↓	16,77	<2.73↓	<3.02↓	36,1	<2.74↓	9,84	<2.06↓	12093	n.d.	957,66	n.d.	<2.85↓
Caco-2_control	<3.08↓	<2.88↓	<2.73↓	<3.02↓	38,61	<2.74↓	<2.42↓	<2.06↓	<3.05↓	20,87	1053	<2.85↓	<2.85↓
Caco-2_LL37	<3.08↓	<2.88↓	<2.73↓	<3.02↓	43,73	<2.74↓	<2.42↓	<2.06↓	3,19	114,78	1050	<2.85↓	<2.85↓
Caco-2_Rumicidin-1	<3.08↓	<2.88↓	<2.73↓	<3.02↓	33,62	<2.74↓	<2.42↓	<2.06↓	19,49	78,32	737,59	<2.85↓	<2.85↓

Statistical analysis of colored values is presented on **Supplementary Fig. 17**

Supplementary Table 7. Analysis of cross-resistance of Rum-1-resistant *E. coli* 1057 (R2) strain to AMPs and conventional antibiotics

<table>
<tr><th colspan="3" rowspan="2">Antibacterial agent</th><th colspan="2">Minimum inhibitory concentration (µM)*</th></tr>
<tr><th>WT
E. coli 1057</th><th>Rum1-res
E. coli 1057 (R2)</th></tr>
<tr><td rowspan="14">Antimicrobial peptides</td><td rowspan="7">proline-rich</td><td>Rumicidin-1</td><td>4</td><td>>64</td></tr>
<tr><td>Rumicidin-2</td><td>8</td><td>>64</td></tr>
<tr><td>Rumicidin-3</td><td>0.5</td><td>4</td></tr>
<tr><td>Bac7[1-22]</td><td>2</td><td>32</td></tr>
<tr><td>mini-ChBac7.5Nα</td><td>4</td><td>>32</td></tr>
<tr><td>PR-39[1-22]</td><td>2</td><td>32</td></tr>
<tr><td>VicBac</td><td>1</td><td>4</td></tr>
<tr><td rowspan="3">α-helical</td><td>LL-37</td><td>2</td><td>2</td></tr>
<tr><td>ChMAP-28</td><td>0.062</td><td>0.062</td></tr>
<tr><td>Melittin</td><td>8</td><td>16</td></tr>
<tr><td rowspan="3">β-hairpin</td><td>Thanatin</td><td>2</td><td>>32</td></tr>
<tr><td>Tachyplesin-1</td><td>0.062</td><td>0.125</td></tr>
<tr><td>Protegrin-1</td><td>0.25</td><td>0.25</td></tr>
<tr><td colspan="2">Polymyxin B</td><td>0.062</td><td>4</td></tr>
<tr><td rowspan="12">Conventional antibiotics</td><td colspan="2">Ampicillin</td><td>>128</td><td>>128</td></tr>
<tr><td colspan="2">Ceftriaxone</td><td>128</td><td>128</td></tr>
<tr><td colspan="2">Ciprofloxacin</td><td>>128</td><td>>128</td></tr>
<tr><td colspan="2">Rifampicin</td><td>32</td><td>32</td></tr>
<tr><td colspan="2">Chloramphenicol</td><td>16</td><td>16</td></tr>
<tr><td colspan="2">Erythromycin</td><td>1024</td><td>1024</td></tr>
<tr><td colspan="2">Clindamycin</td><td>128</td><td>256</td></tr>
<tr><td colspan="2">Tetracycline</td><td>4</td><td>4</td></tr>
<tr><td colspan="2">Streptomycin</td><td>4</td><td>4</td></tr>
<tr><td colspan="2">Kanamycin</td><td>8</td><td>8</td></tr>
<tr><td colspan="2">Gentamycin</td><td>2</td><td>2</td></tr>
<tr><td colspan="2">Spectinomycin</td><td>64</td><td>64</td></tr>
</table>

* The experiment was performed in the Mueller-Hinton broth supplemented with 0.9% NaCl at 37°C

Supplementary Table 8. Oligonucleotide primers used in this study

Name	Sequence 5'→3'	Description
Bac-f	GCAGATCTATGCGCCGTATTCGTCCACGTCCACCTCGTTTGCCTCGCCCGCGT	Synthesis of fragment encoding **Bac7[1-22]** followed by insertion into pET expression plasmid
Bac-r	GCGAATTCTTAACGTGGAAAAGGTAATGGACGAGGACGCGGGCGAGGCAAACGAG	
PR-f	GCAGATCTATGCGTCGCCGTCCACGCCCACCTTATCTGCCGCGTCCACGTCCA	Synthesis of fragment encoding **PR-39[1-22]** followed by insertion into pET expression plasmid
PR-r	GCGAATTCTTAAAGACGTGGAGGAAAAAACGGCGGTGGACGTGGACGCGGCAGAT	
Rum1-f1	GCAGATCTATGCGTGCTCGTCGCCCACGTCCTCCGAAACCGCGTCCTCCGCAT	Synthesis of fragment encoding **Rum-1[1-22]** followed by insertion into pET expression plasmid
Rum1-r1	GCGAATTCTTACGGACGTGGGCGCGGACGAGGACGATGCGGAGGACGCGGTTTCG	
Rum1-f2	TGGTTTCCACCTCGTTTTCCTTAAGAATTCTCTCGAGCACCA	Synthesis of plasmid encoding **Rum-1** by one-step LIC from plasmid encoding Rum-1[1-22]
Rum1-r2	AGGAAAACGAGGTGGAAACCACGGACGTGGGCGCGGACG	
Rum1-f3	CATCGTCCTTAAGAATTCTCTCGAGCA	Synthesis of plasmid encoding **Rum-1[1-16]** by one-step LIC from plasmid encoding Rum-1[1-22]
Rum1-r3	GAATTCTTAAGGACGATGCGGAGGACG	
Rum1-f4	GGATCTATGAAACCGCGTCCTCCGCAT	Synthesis of plasmid encoding **Rum-1[9-29]** by one-step LIC from plasmid encoding Rum-1
Rum1-r4	ACGCGGTTTCATAGATCCGGCCAGGTTAGC	
Rum1-f5	CGTCCTCCGGCACGTCCTCGTCCGCG	Synthesis of plasmid encoding **Rum-1[H14A]** by one-step LIC from plasmid encoding Rum-1
Rum1-r5	ACGAGGACGTGCCGGAGGACGCGGTTTC	
Rum1-f6	CCACGTCCGGCATTTCCACCTCGTTTTCC	Synthesis of plasmid encoding **Rum-1[W23A]** by one-step LIC from plasmid encoding Rum-1
Rum1-r6	AGGTGGAAATGCCGGACGTGGGCGCG	
Rum1-f7	CGCCCACGTGCATGGTTTCCACCTCGTTT	Synthesis of plasmid encoding **Rum-1[P22A]** by one-step LIC from plasmid encoding Rum-1
Rum1-r7	TGGAAACCATGCACGTGGGCGCGGACGA	
Rum1-f8	ACGTCCGGCAGCTCCACCTCGTTTTCCTTA	Synthesis of plasmid encoding **Rum-1[W23A, F24A]** or **Rum-1[9-29, W23A, F24A]** by one-step LIC from plasmid encoding Rum-1 or Rum-1[9-29], respectively
Rum1-r8	GAGGTGGAGCTGCCGGACGTGGGCGCGGA	
Rum1-f9	TCCTCCGCGTCTTCCTCGTCCGCGCCCACGT	Synthesis of plasmid encoding **Rum-1[H14R, R15L]** by one-step LIC from plasmid encoding Rum-1
Rum1-r9	GACGAGGAAGACGCGGAGGACGCGGTTTCGGA	
Rum1-r10	CATAGATCCGGCCAGGTTAGCGTCGA	Universal primer for the synthesis of plasmid encoding **Rum-1[4-29]**, **Rum-1[6-29]**, **Rum-1[11-29]**, **Rum-1[13-29]**, or **Rum-1[15-29]** by one-step LIC from plasmid encoding Rum-1
Rum1-f10	AACCTGGCCGGATCTATGCGCCCACGTCCTCCGAAA	Synthesis of plasmid encoding **Rum-1[4-29]** by one-step LIC from plasmid encoding Rum-1
Rum1-f11	AACCTGGCCGGATCTATGCGTCCTCCGAAACCGCGT	Synthesis of plasmid encoding **Rum-1[6-29]** by one-step LIC from plasmid encoding Rum-1
Rum1-f12	AACCTGGCCGGATCTATGCGTCCTCCGCATCGTCCT	Synthesis of plasmid encoding **Rum-1[11-29]** by one-step LIC from plasmid encoding Rum-1
Rum1-f13	AACCTGGCCGGATCTATGCCGCATCGTCCTCGTCCG	Synthesis of plasmid encoding **Rum-1[13-29]** by one-step LIC from plasmid encoding Rum-1
Rum1-f14	AACCTGGCCGGATCTATGCGTCCTCGTCCGCGCCCA	Synthesis of plasmid encoding **Rum-1[15-29]** by one-step LIC from plasmid encoding Rum-1
Rum2-f1	GCAGATCTATGCGTGATCGTCGCCGTCGTCCTCCGCGCCCGCGTCCTCCGCAT	Synthesis of fragment encoding Rum-2[1-22] followed by insertion into pET expression plasmid
Rum2-r1	GCGAATTCTTACGGCAGTGGGCGCGGACGAGGACGATGCGGAGGACGCGGGCGCG	
Rum2-f2	TGGTTTCCACCTCGTTTTCCTTAAGAATTCTCTCGAGCACCA	Synthesis of plasmid encoding **Rum-2** by one-step LIC from plasmid encoding Rum-2[1-22]
Rum2-r2	AGGAAAACGAGGTGGAAACCACGGCAGTGGGCGCGGACG	
Rum3-f1	GCAGATCTATGCGTATTAAAATCCCACGTCCTCCGAAACCGCGTCGCCCGCATCGT	Synthesis of fragment encoding Rum-3[1-23] followed by insertion into pET expression plasmid
Rum3-r1	GCGAATTCTTACGGACGACGGCGACCCGGACGAGGACGATGCGGGCGACGCGGTT	
Rum3-f2	TGGTTTCCACCTCGTTTTCCTTAAGAATTCTCTCGAGCACCA	Synthesis of plasmid encoding **Rum-3** by one-step LIC from plasmid encoding Rum-3[1-23]
Rum3-r2	AGGAAAACGAGGTGGAAACCACGGACGACGGCGACCCGGA	
SbmA-f	GTCGAAACAATTCTTATGGTCAG	Amplification of *sbmA* gene of *E. coli* (TA-cloning followed by sequencing)
SbmA-r	GCGAAGATAGAGGATTGACGCG	
RpoS-f	TGAATGTTCCGTCAAGGGAT	Amplification of *rpoS* gene fragment of *E. coli* (TA-cloning and validating of genome sequencing data)

RpoS-r	CCAGACCACGATTGCCATA	
MacB-f1	AGAGTCATTGGTGTGGCGGAAGA	Amplification of *macB* gene fragment of *E. coli* (TA-cloning and validating of genome sequencing data)
MacB-r1	GTGTACATCCTAAAGGCAAAATGC	
MacB-f2	AGAAATACTAGATGACGCCTTTGCTCGAATTAAAGG	Amplification of *macB* gene of *E. coli* to insert into pUC57 plasmid by one-step LIC
MacB-r2	ATCCCAGGTTACTCCCGTGCCAGAGCATCT	
MacB-f3	AGAAATTAACTATGACGCCTTTGCTCGAATTAAAG	Amplification of *macB* gene of *E. coli* to insert into pQE30 plasmid by one-step LIC
MacB-r3	AAGCTCAGTTACTCACGTGCCAGAGCATCT	
MacB-f4	GCCCATGGTGACGCCTTTGCTCGAATTAAAGG	Amplification of *macB* gene of *E. coli* to insert into pBAD plasmid by NcoI/EcoRI (underlined)
MacB-r4	CGGAATTCTTACTCCCGTGCCAGAGCATCT	
pUC-f	TGGCACGGGAGTAACCTGGGATCAAATAAAACGAA	Amplification of pUC57 plasmid for one-step LIC with *macB*
pUC-r	CAAAGGCGTCATCTAGTATTTCTCCTCTTTCTCG	
pQE-f	TGGCACGTGAGTAACTGAGCTTGGACTCCTGT	Amplification of pQE30 plasmid for one-step LIC with *macB*
pQE-r	CAAAGGCGTCATAGTTAATTTCTCCTCTTTAATGAA	
Erm-f	ACTAATACGACTCACTATAGGGAGTTTTATAAGGAGGAAAAAATATGGGCATTTTT AGTATTTTTGTAATCAGCACAGTTCATTATCAA	Assembly of ErmCL template for Toe-printing analysis
Erm-r	GGTTATAATGAATTTTGCTTATTAACGATAGAATTCTATCACTTTTTTTATTATTA TTATTTTTTGTTTGGTTGATAATGAACTGTGCT	

Supplementary Discussion

Identification of genes encoding rumicidins.

Among the found sequences, the major part belonged to pseudogenes (ψ), and only a quarter (18 of 65) of the species had presumably intact genes (**Supplementary Fig. 1**) which displayed all the characteristics of functional cathelicidin genes, including (i) a gene size of about 2 kb, (ii) a conserved four exons/three introns arrangement with intact splicing sites, (iii) TATA box located immediately upstream from the transcription start site, (iv) a polyadenylation signal located about 100 bp away from the stop codon. For all the intact and virtually active genes mutations affecting gene expression, in particular, cis-regulatory mutations cannot be excluded. Analysis of *ψCATHL(3L2/8)* pseudogenes revealed different variants of gene inactivation, including nonsense mutations (premature termination codon, indel) as well start codon (Met) mutations (**Supplementary Fig. 2**). In most cases, these aberrant mRNAs are likely degraded by the nonsense-mediated decay (NMD) pathway[2]. We also found that nonsense mutations often accompany the downstream mutations in the 5' splice site which caused the intron retention. As NMD occurs, if a premature termination codon is located ≥55 nucleotides upstream of an exon-exon junction, it can probably increase the efficiency of the mRNA degradation[2]. The detailed analysis of presumably intact *CATHL(3L2/8)* genes and *ψCATHL(3L2/8)* pseudogenes is presented in the file "**Supplementary Data**". Such a widespread pseudogenization becomes possible likely due to a co-expression of different PrAMPs with overlapping functions. Thus, the majority of analyzed species have Bac7- and Bac5-like genes, both inhibiting bacterial translation[3], while Cervidae, lacking both Bac7-like and intact *CATHL(3L2/8)* genes, have the genes encoding Bac5 orthologs and other structural families of PrAMPs.

Bacterial resistance to rumicidin-1

To cross the outer and inner membrane some peptide bacteriocins and short PrAMPs use the OmpF porin and SbmA transporter, respectively[4,5]. Our results suggest that rumicidin-1 is transported inside sensitive *E. coli* cells via OmpF and SbmA. The corresponding *E. coli* strains lacking *ompF* or *sbmA* genes demonstrated a ≥4-fold increase in the MIC value (**Fig. 5a**). However, other ways to reach the cytoplasm shall not be excluded. It might be supposed that an additional *C*-terminal hydrophobic extra piece is likely needed to damage and/or cross the cytoplasmic membrane and effectively inhibit SbmA-deficient strains as was shown for Bac7[1-35] and other mammalian Bac7-like peptides[5–7]. By contrast, the *E. coli* SQ110 Δ*lptD* strain with compromised outer membrane was 4-fold more sensitive to rumicidin-1 as compared to the parent *E. coli* SQ110 strain. Knockout of the gene encoding MdtM, a transporter system located in the inner bacterial membrane of *E. coli*, did not result in increase of MIC of rumicidin-1 contrary to insect oncocins[8], thus suggesting a key role of SbmA in rumicidin transport across the membrane.

We also showed that MacAB-TolC pump and its mutant variant (**Supplementary Fig. 11**) played a role in the development of resistance to rumicidins and other PrAMPs (**Supplementary Table 6**). Unlike specific transporters that have well-structured binding sites, multidrug resistance (MDR) efflux pumps like MacAB-TolC contain so-called "vestibules", large cavities that can bind many different compounds[9] and, therefore, can be structurally adapted to facilitate selectivity against these cationic AMPs. A set of single MacA mutations were observed in the sequenced *E. coli* strains with the induced resistance to the porcine proline-rich cathelicidin PR-39[10]. An important role of MacAB and TolC in bacterial adaptation to cationic AMPs was also proved by an extremely low rate of transposon integration in corresponding genes in the presence of arenicin-3[11]. Previous studies have also shown that MacAB homologs can contribute to increasing the resistance to membrane-targeting antimicrobial peptides in other Enterobacteria[12]. Notably, in *E. coli*, the TolC-dependent secretion of amphiphilic peptide enterotoxin STII is facilitated by MacAB but not by other TolC-interacting partners[13].

Immunomodulatory activity of rumicidin-1 in comparison with LL-37

Rumicidin-1 was shown to display both pro- and anti-inflammatory action similar to human cathelicidin LL-37 (**Supplementary Fig. 17**). It was shown that an anti-inflammatory response of both cathelicidins was mediated by inhibition of production of the major pro-inflammatory cytokine TNFα by monocytes. At the same time, both cathelicidins also showed a pro-inflammatory action on peripheral blood mononuclear cells (PBMC) by inhibiting the anti-inflammatory soluble interleukin-1 receptor antagonist (IL-1RA) production. In monocytes, such inhibition of IL-1RA was observed only in case of rumicidin-1. It is known that TNF-α and IL-1 are usually produced together as a prompt response to bacterial infections. Expectedly, the most of significant effects on human cells were specifically triggered by LL-37 (**Supplementary Fig. 17**). However, we also found a number of pro-inflammatory effects unique to rumicidin-1. In particular, only rumicidin-1 was shown to be able to inhibit production of RANTES, also known as the C-C motif chemokine 5 (CCL5), by PBMC and monocytes. RANTES/CCL5 is chemotactic for T cells, eosinophils, and basophils and plays an important role in recruiting leukocytes into inflammatory sites[14]. We also showed that rumicidin-1, but not LL-37, was able to induce production of the chemotactic IL-8/CXCL8 from below the detectable minimum of <3.05 to 19.49 pg/mL (p=0.021) by the epithelial Caco-2 cells. Production of the pro-inflammatory eosinophil chemotactic protein-1 (Eotaxin-1), which selectively recruits eosinophils to the site of pathogen invasion and is a key regulator of intestinal inflammation, by PBMC was significantly decreased by rumicidin-1 that is similar to effect of the Bac7-like PrAMP VicBac from alpaca [5].

Supplementary References

1. Panteleev, P. V. *et al.* Combined Antibacterial Effects of Goat Cathelicidins With Different Mechanisms of Action. *Front. Microbiol.* **9**, 2983 (2018).
2. Kurosaki, T., Popp, M. W. & Maquat, L. E. Quality and quantity control of gene expression by nonsense-mediated mRNA decay. *Nat. Rev. Mol. Cell Biol.* **20**, 406–420 (2019).
3. Mardirossian, M., Sola, R., Degasperi, M. & Scocchi, M. Search for Shorter Portions of the Proline-Rich Antimicrobial Peptide Fragment Bac5(1–25) That Retain Antimicrobial Activity by Blocking Protein Synthesis. *ChemMedChem* **14**, 343–348 (2019).
4. Metelev, M. *et al.* Klebsazolicin inhibits 70S ribosome by obstructing the peptide exit tunnel. *Nat. Chem. Biol.* **13**, 1129–1136 (2017).
5. Panteleev, P. V. *et al.* A Novel Proline-Rich Cathelicidin from the Alpaca Vicugna pacos with Potency to Combat Antibiotic-Resistant Bacteria: Mechanism of Action and the Functional Role of the C-Terminal Region. *Membranes* **12**, 515 (2022).
6. Sola, R. *et al.* Characterization of Cetacean Proline-Rich Antimicrobial Peptides Displaying Activity against ESKAPE Pathogens. *Int. J. Mol. Sci.* **21**, 7367 (2020).
7. Mardirossian, M. *et al.* The Dolphin Proline-Rich Antimicrobial Peptide Tur1A Inhibits Protein Synthesis by Targeting the Bacterial Ribosome. *Cell Chem. Biol.* **25**, 530-539.e7 (2018).
8. Krizsan, A., Knappe, D. & Hoffmann, R. Influence of the *yjiL-mdtM* Gene Cluster on the Antibacterial Activity of Proline-Rich Antimicrobial Peptides Overcoming Escherichia coli Resistance Induced by the Missing SbmA Transporter System. *Antimicrob. Agents Chemother.* **59**, 5992–5998 (2015).
9. Alav, I. *et al.* Structure, Assembly, and Function of Tripartite Efflux and Type 1 Secretion Systems in Gram-Negative Bacteria. *Chem. Rev.* **121**, 5479–5596 (2021).
10. Spohn, R. *et al.* Integrated evolutionary analysis reveals antimicrobial peptides with limited resistance. *Nat. Commun.* **10**, 4538 (2019).
11. Elliott, A. G. *et al.* An amphipathic peptide with antibiotic activity against multidrug-resistant Gram-negative bacteria. *Nat. Commun.* **11**, 3184 (2020).
12. Honeycutt, J. D. *et al.* Genetic variation in the MacAB-TolC efflux pump influences pathogenesis of invasive Salmonella isolates from Africa. *PLOS Pathog.* **16**, e1008763 (2020).
13. Yamanaka, H., Kobayashi, H., Takahashi, E. & Okamoto, K. MacAB Is Involved in the Secretion of *Escherichia coli* Heat-Stable Enterotoxin II. *J. Bacteriol.* **190**, 7693–7698 (2008).
14. Bishara, N. The Use of Biomarkers for Detection of Early- and Late-Onset Neonatal Sepsis. in *Hematology, Immunology and Infectious Disease: Neonatology Questions and Controversies* 303–315 (Elsevier, 2012). doi:10.1016/B978-1-4377-2662-6.00018-3.

Uncropped SDS-PAGE gel (Supplementary Fig. 3)

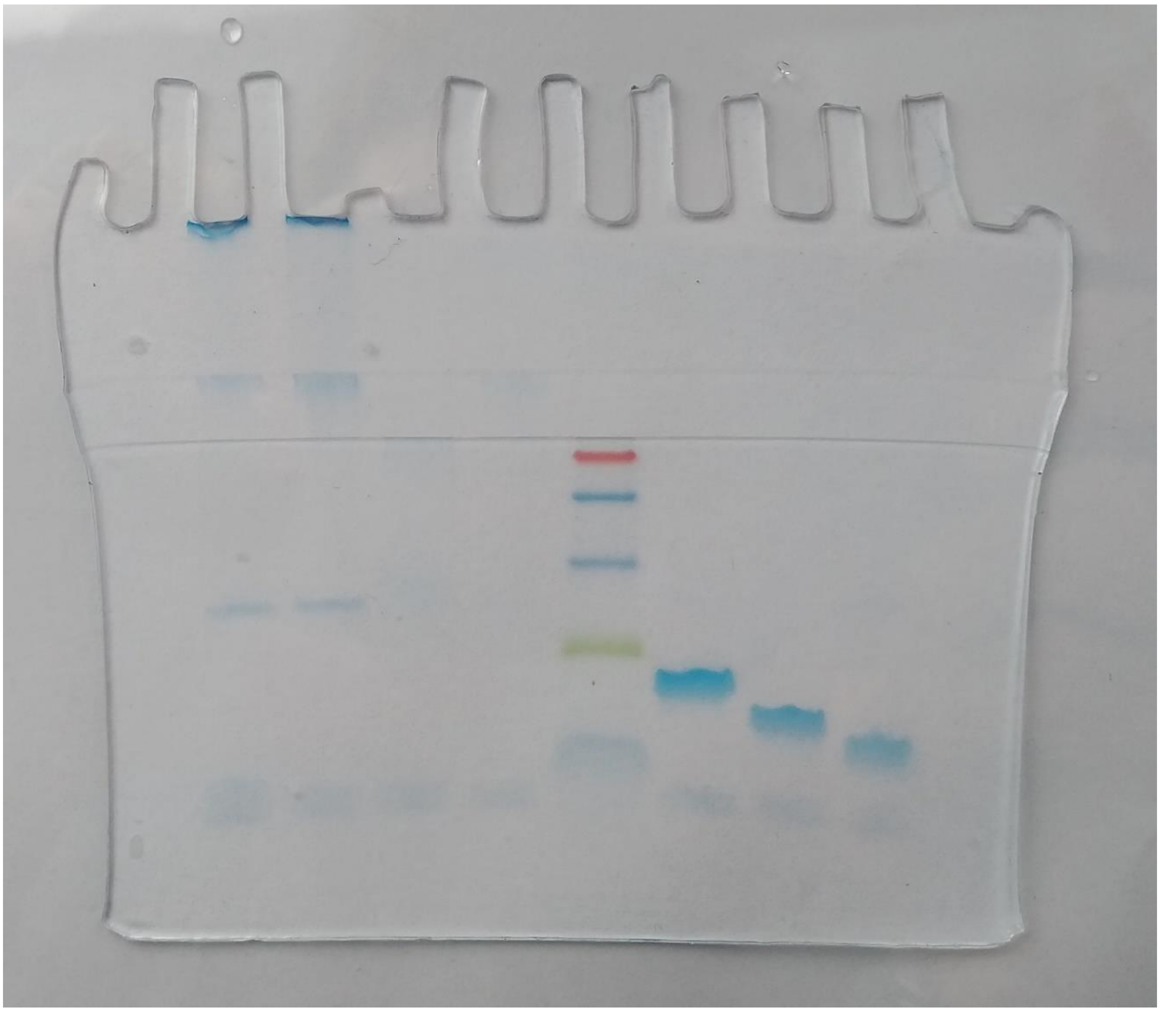

Uncropped autoradiograph of toeprinting gel (Supplementary Fig. 4)

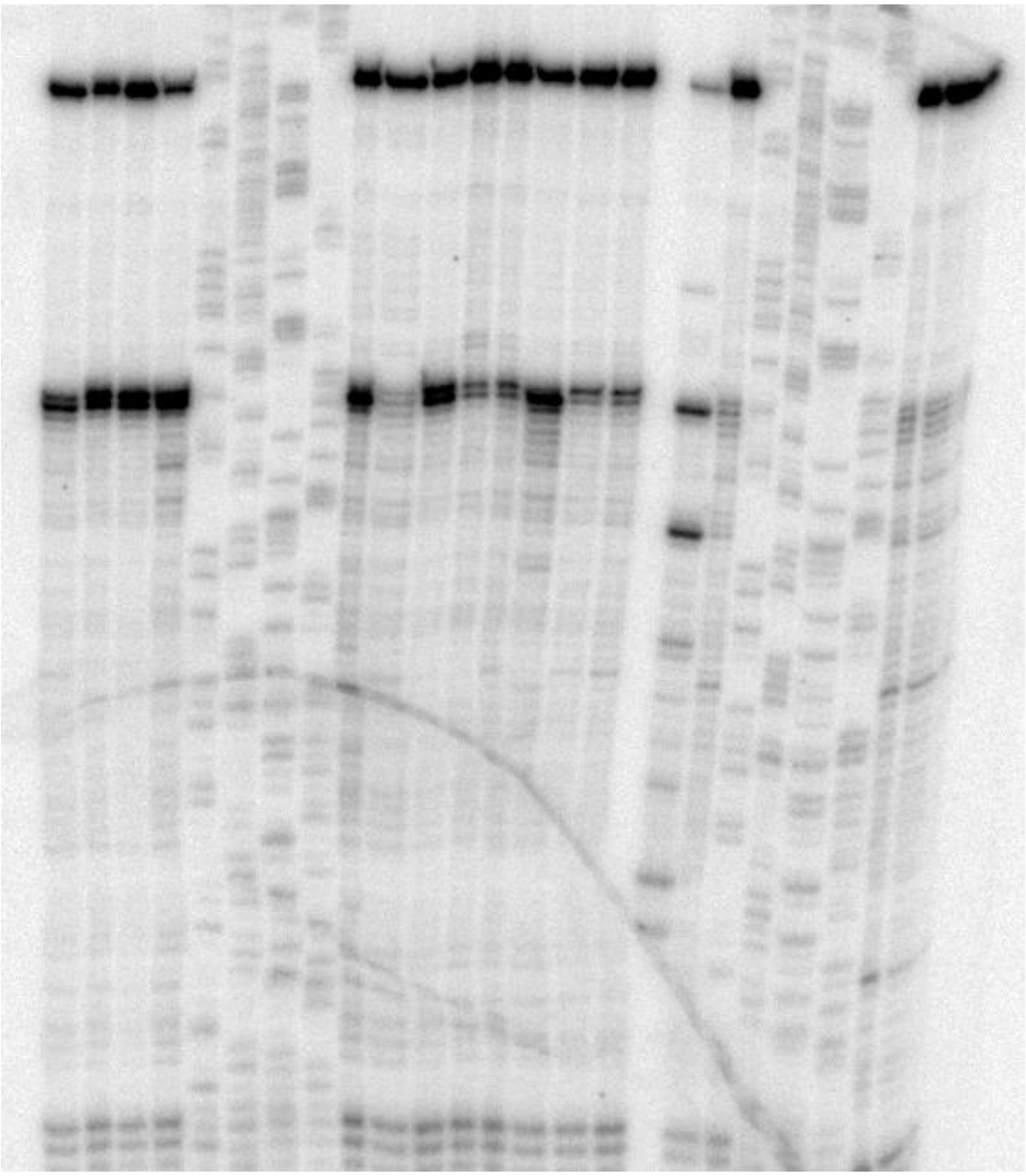

Supplement: Supplementary file 1 — Supplementary Information [file 41467_2024_53309_MOESM1_ESM.pdf]
