# Supplementary material for: Rumicidins are a family of mammalian host-defense peptides plugging the 70S ribosome exit tunnel
Source: Nat Commun. 2024 Oct 16;15:8925. doi: 10.1038/s41467-024-53309-y (PMC11484942; doi:10.1038/s41467-024-53309-y)
Supplement: Supplementary file 2 — Description of Additional Supplementary Files [file 41467_2024_53309_MOESM2_ESM.pdf]

Description of Additional Supplementary Files

File name: Supplementary Data 1

Description: Analysis of the presumably intact CATHL(3L2/8) genes and the pseudogenes ψCATHL(3L2/8) found in Cetartiodactyla. Description: exons are highlighted in green; introns are highlighted in light grey; possible intron retention is highlighted in yellow; nonsense mutations (premature termination codon, indel), splice site mutations and start codon mutations are highlighted in purple. * - TATA-box and polyadenylation signal are highlighted with grey color.
