# Supplementary material for: Rumicidins are a family of mammalian host-defense peptides plugging the 70S ribosome exit tunnel
Source: Nat Commun. 2024 Oct 16;15:8925. doi: 10.1038/s41467-024-53309-y (PMC11484942; doi:10.1038/s41467-024-53309-y)
Supplement: Supplementary file 3 — Supplementary Data 1 [file 41467_2024_53309_MOESM3_ESM.pdf]

Supplementary Data

Analysis of the presumably intact *CATHL(3L2/8)* genes and the pseudogenes ψ*CATHL(3L2/8)* found in Cetartiodactyla

Description: exons are highlighted in green; introns are highlighted in light grey; possible intron retention is highlighted in yellow; nonsense mutations (premature termination codon, indel), splice site mutations and start codon mutations are highlighted in purple.

Litocranius walleri (2 gene copies)

GenBank: SJYG01000096.1

METQKASLSLGRCLLWLLLLGLVLPSASAQALSYREAVLHAVDRINEQSSEANLYRLLELDPPLKDVEDSGARKPVSFRVKETVCPRPSQQPAEQCDFKENGLVKQCVGTVSLDPADDQFDLNCNELQSVR
SRRRPSKPRRPHRPRRRPRPWFPPRFRGKQ

atggagacccagaaggccagcctctcgctgggacggtgtttgctgtggctcctgctgctgggactagtgctgccctcggccagcgcccaggccctcagctacagggaggctgttctacatgctgtggaccg
catcaatgagcagtcctcagaagctaatctctaccgcctcctggagcttgacccgcctctcaaggacgtgagttgggggatagggtggggaggggccgtctcttgcttgccttggccgcattctcactcc
tttcactctggctggtcctcctgttaggaaggctcttttccctttaggcgggctcccacctcttccaggaaaccttcccagacctgggtcatctcccagcaccaggcttcctgcctcagcatctctgctgt
gggatcaggcgccctgcacacctggctcaggctcccgggacttctgggagctccagggatggaggggtcacaggctctgtgaggtgacttccctgctaatgccctctgcatctcggtgtctccctgccag
gagggcctctgtcagcctggaggctccagtgacaagggctctccctgcaggcggccctgacctccctgggcccctctgaggggaggcgctgccagcagcgctgctgtgagggccgctcctgctctctgtgt
gcccgtgaggccgggcacgggctctgggccctcccctgcgctcccagcaccaagcccagggccggacacacaggggggctcgagaggctgccatctgggttggggggcagggagacagatcagagaagggaac
atgagccgagcccagtctccccactttgatcgttgaccaggtggaggactcgggagctcgaaagcctgtgagcttcagggtgaaggagactgtgtgccccaggccgagccagcagcccgcggagcagtgtg
acttcaaggagaatggggtgagcctgggggcgaggagtgagggctgggatcagtgcttctgagtgcaagctgaacagggagcttcagggacgatttccagcccctggggggtgaggtgggctgagcctgga
aggttatggcctgggggttccagtttgaccttgagctccatttgcagctggtgaaacagtgtgtggggacagtcagcctggacccggccgatgaccagtttgacctgaactgtaatgaggtgagtggcccc
ttctgtgttgggcagatgctaaaaagggtgggttgagaagcatcctttggaccaatgacctgctgctgctccgtctagggcagagagaaggccctcctgcctgggcccaccctccccagtccccaggcctc
cagccctggccctgcatcccttacagaagtgtctgtctactggggtccccacccaggaactgacagtaaggcagattctcagccccgctgagacctcctgaatcagactttggggtgggacccgggcattt
gtattttcacaagacctccagcggattctgacagtgttgaaattgtgacaccctgactcgagtcatggtcttgaggccatgctccagtccatctttgcccacatgggcttgtgacccttggaagccccttg
tcatctctgggatcagtttccccatatgttgtaggtttaggaattcaaccacatgccccaaagatcactgacagaggatgacctggggccaaagttcctttggtggctcagtttggggggttgttcaggtgg
ggagagagtggtcttctcttgacccttgtccagtcccacaagtaatctcttccattgtggttcacagcttcagagtgtcaggtctagacgacggccttccaaacctcggcgtcctcacaggccacgccgaa
ggccacggccatggttcccaccaaggttccgcggaaaacagtga

GenBank: SJYG01000114.1

METQRASLSLGRCLLWLLLLGLVLPSASAQALSYREAVLHAVDRINEQSSEANLYRLLELDPPLKDVEDSGARKPVSFRVKETVCPRPSQQPAEQCDFKENGLVKQCVGTVSLDPADDQFDLNCNELQSVR
SRRRPSKPRRPHRPRRRPRPWFPPRFRGKQ

ccaggctgagcataaaggcagatcctgcagtctgggaggaggcaaactggggaccatggagacccagaggccagcctctcgctgggacggtgtttgctgtggctcctgctgctgggactagtgctgccctcggccagcgcccag
gccctcagctacagggaggctgttctacatgctgtggaccgcatcaatgagcagtcctcagaagctaatctctaccgcctcctggagcttgacccgcctctcaaggacgtgagttgggggatagggtggggaggggccgtctct
tgcttgccttggccgcattctcactcctttcactctggctggtcctcctgttaggaaggctcttttccctttaggcgggctcccacctcttccaggaaaccttcccagacctgggtcatctcccagcaccaggcttcctgcctca
gcatctctgctgtgggatcaggcgccctgcacacctggctcaggctcccgggacttctgggagctccagggatggaggggtcacaggctctgtgaggtgacttccctgctaatgccctctgcatctcggtgtctccctgccagg
agggcctctgtcagcctggaggctccagtgacaagggctctccctgcaggcggccctgacctccctgggcccctctgaggggaggcgctgccagcagcgctgctgtgagggccgctcctgctctctgtgtgcccgtgaggccggg
cacgggctctgggccctcccctgcgctcccagcaccaagcccagggccggacacacaggggggctcgagaggctgccatctgggttggggggcagggagacagatcagagaagggaacatgagccgagcccagtctccccactttga
tcgttgaccaggtggaggactcgggagctcgaaagcctgtgagcttcagggtgaaggagactgtgtgccccaggccgagccagcagcccgcggagcagtgtgacttcaaggagaatggggtgagcctgggggcgaggagtgaggg
ctgggatcagtgcttctgagtgcaagctgaacagggagcttcagggacgatttccagcccctggggggtgaggtgggctgagcctggaaggttatggcctgggggttccagtttgaccttgagctccatttgcagctggtgaaac
agtgtgtggggacagtcagcctggacccggccgatgaccagtttgacctgaactgtaatgaggtgagtggccccttctgtgttgggcagatgctaaaaagggtgggttgagaagcatcctttggaccaatgacctgctgctgctc
cgtctagggcagagagaaggccctcctgcctgggcccaccctccccagtccccaggcctccagccctggccctgcatcccttacagaagtgtctgtctactggggtccccacccaggaactgacagtaaggcagattctcagccc
cgctgagacctcctgaatcagactttggggtgggacccgggcatttgtattttcacaagacctccagcggattctgacagtgttgaaattgtgacaccctgactcgagtcatggtcttgaggccatgctccagtccatctttgcc
cacatgggcttgtgacccttggaagccccttgtcatctctgggatcagtttccccatatgttgtaggtttaggaattcaaccacatgccccaaagatcactgacagaggatgacctggggccaaagttcctttggtggctcagtt
tggggggttgttcaggtggggagagagtggtcttctcttgacccttgtccagtcccacaagtaatctcttccattgtggttcacagcttcagagtgtcaggtctagacgacggccttccaaacctcggcgtcctcacaggccacgc
cgaaggccacggccatggttcccaccaaggttccgcggaaaacagtgaaggactggctgtcacacctattaatggcttttggtgaattccgagcccgagggaagccttaaagatatgatttgttctggctcagagttctggacgg
taaaaaataaattc

* - TATA-box and polyadenylation signal are highlighted with grey color

Hippotragus niger niger

METQSASLSLERCSLWLLLLGPALPSASAQAPSYREAVLRAVDRINDGSTEANLYRLLELDPPPRDVEDRGARKPVSFRVKETVCPRPSQQPEEQCDFKENGLVKQCLGTVSLDPSDDQFDINCNELQSIR
ARRPRPPKPRPPHRPRPRPLPWFPPWFLGKR

GenBank: VHQK01014889.1

aggctgagcataaaaggagggtcgctcgggctgggaggaggcagactggggaccatggagacccagagcgccagcctctccctggaacggtgttcgctgtggctactgctgctgggaccagcactgccctc
agccagcgcccaggcccccagctacagggaggctgtgcttcgtgctgtggatcgcatcaatgatgggtccacagaagctaatctctaccgcctcctggagcttgaccctcctcccagggacgtgagttggg
gagagtgctgagccatcttcccccagccttggccacactgtcgccccttcgctcaggctggtcctcctgtcaggaaggcactttctctctaggtaggttaaccttccgagacctgggtcatctcccagcac
caggcttcctgtcttagcatctctgctgtgggatcaggcaccctgcacacctggctccggctccctggacttctgggagctccagggatggaggggtcacaggctctgtgaggtgacttccctgctaatgt
cccccctgctccttggtgtctccctgccaggaggcctctgtcagcctggaaggtccagtgacgagggctctccctgctggcggccctgacctccctgggcccctctgaggggaggcgctgccagcagcgct
gctgtgagggccgcttctgctctctgtgtgcccgtgaggccgggcacgggctccgtgccctcccctgtgctcctagcaccaagcccagggccggacacacaggggctggagaggctgccgtctgtgtggg
gggcagggagacagatgagagaaggaaacgtgagcccgagcccagtctccccactttgatcgttgaccaggtggaggaccggggagctcgaaagcctgtgagcttcagggtgaaggagactgtgtgcccca
ggccaagccagcagcccgaggagcagtgtgacttcaaggagaatggggtgagcctggggcagggagtgagggctgggatcaatgcttctcagtgctagctgaacagggaacttcagggacgatttccagc
ccctggcgggtgaggtgggctgagcctgggaggttatggcccagggtttccagtttgacctggaaactctccttccagttggtgaaacagtgtttggggacagtcagcctggacccatccgatgaccagtt
tgacataaactgtaatgaggtgagtggccccttctgtgttggacatatgctaacaaggtgggttgagaaacatcctttggaccaatgacccgctgctccatgtagggcagagaaaaggccctcctacccgg
gcccaccctccccagtccccaggcctccagccctggctctgcatccctaagagaagtggctgtctaacagggtccccacccgggaactgacagtaaggcagattctcagccccactgagacctcctgaatc
agactttggggtgggacccaggcatttgtattttctcaaggcctccaggggattctgacagtgctgaagttgtgacaccctgactcgagtcatggtcttgaggccatgctccagtccatctttgcccagat
gggcttgtgacccttggaagccccttgtcatctctgggatcagtttccccatatgttgtgggtttaggaattcaaccacatgccccaaagatcaccaccagaagatgatctggagccaaagttcctttggt
ggctcagtttgggagttgttcaggtggggagagagtggtcttctcttgactcttgcccaatcccacaagtaatctcttccattgtggttcacagcttcagagtatcagggctagacgacctcggcctccca
aacctcggcctcctcacaggccaaggccaaggccactgccatggttcccgccatggttcctgggaaaacggtgaaggactggctgtcacacctattaatggcttttggtgaattccgagactgagggaagc
attttaaagatatgatatgttctggctcagacttctggacggtgaaaaataaattcttatgaaaac

* - TATA-box and polyadenylation signal are highlighted with grey color

Hippotragus equinus

VEDRGARKPVSFRVKETVCPRPSQQPLEQCDFKENGLVKQCLGTVSLDPSDDQFDINCNELQSVRARRPRPPKPRPPHRPRPRPLPWFPPWFLGKR

GenBank: JAEFBM010009657.1

contig_start_gaaacatgagcccgagcccagtctccccactttgatcgttgaccaggtggaggaccggggagctcgaaagcctgtgagcttcagggtgaaggagactgtgtgccccaggccaagccag
cagcccctggagcagtgtgacttcaaggagaatggggtgagcctgggggcagggagtgagggctgggatcaatgcttctcagtgctagctgaacagggaacttcagggacgatttccagcccctggcgggt
gaggtgggctgagcctgggaggttatggcccagggtttccagtttgacctggaaactctccttccagttggtgaaacagtgtttggggacagtcagcctggacccatccgatgaccagtttgacataaact
gtaatgaggtgagtggccccttctgtgttgggcatatgctaacaaggtgggttgagaaacatcctttggaccaatgacccgctgctccatgtagggcagagaaaaggccctcctacccaggcccaccctcc
ccagtccccaggcctccagccctggctctgcatccctaagagaagtggctgtctaacagggtccccacccgggaactgacagtaaggcagattctcagccccactgagacctcctgaatcagactttgggg
tgggacccaggcatttgtattttctcaaggcctccaggggattctgacagtgctgaagttgtgacaccctgactcgagtcatggtcttgaggccatgctccagtccatctttgcccagatgggcttgtgac
ccttggaagccccttgtcatctctgggatcagtttccccatatgttgtgggtttaggaattcaaccacatgccccaaagatcaccgccagaagatgatctggagccaaagttcctttggtggctcagtttg
ggagttgttcaggtggggagagagtggtcttctcttgactcttgcccaatcccacaagtaatctcttccattgtggttcacagcttcagagtgtcagggctagacgacctcggcctcccaaacctcggcct
cctcacaggccaaggccaaggccactgccatggttcccgccatggttcctgggaaaacggtga

Pantholops hodgsonii

METHRASLSLGRWSLWLLLLALVLPSASAQALSYREAVLRAVDRINDGSTEANLYRLLELDLPPKDVEDRGARKPVSFRVKETVCPRTTQQPAECDFKENGLVKQCLGTVSLDQSDDQFDINCNELQSVR
ARRPRPPKPRPPHRPRPRPRPWFPPRFPGKR

GenBank: JBBYXG010000023.1

atggagacccacagggccagcctctccctgggacgctggtcactgtggctactgctgctggcactagtgctgccctcggccagcgcccaggccctcagctacagggaggctgtgcttcgtgctgtggatcg
catcaatgatgggtccacagaagctaatctctaccgcctcctggagcttgacctgcctcccaaggatgtgagttggggagggtgctgagcaatcttcccccagccttggccacactgtcaccccctcgctc
aggctggtcctcctgtcaggaaggcacttttccctctaggtgggctcccacctcttccaggaatccttcccagacctggggcccctcccagcaccgcgcttcctgccttagcatctctgctgtgggatcag
gcgccctgcacacctggctccctggacttctgggagctccagggatggaggggtcacaggctctgtgaggtgacttccctgctaatgtccccctgctcctcgctgtctccctgccaggagggcctctgtc
agcctggaggcgctccctgcaggtggccctgacctccctgggcccctctaaggggaggccctgccagcagcactgctgtgaggctgctcctgctctctgtgtgcccgtgaggccgggcacgggctctgtg
ccctcccctgtgctcccagcaccaagcccagggcctgacacacaggggctggagaggctgccgtctgggtgggggcagggagacagatcagagaaggaaacatgagcccaagcccagtctccccacttt
gatcattgaccaggtggaggaccggggagctcgaaagcctgtgagcttcagggtgaaggagactgtgtgccccaggacgacccagcagcccgcggagcagtgtgacttcaaggagaatggggtgagcctgg
gggcagggagtgagggctgggatcaatgcttctcagtgctagccgaacagggagcttcagggacgatttccagcccctgggggtgaggtgggctgagcctgggagggtatggcccagggtttccagtttg
acctggaaactccccttccagctggtgaaacagtgtttggggacagtcagcctggaccagtccgatgaccagtttgacataaactgtaatgaggtgagtggccccttctgtgttgggcatatgctaacaag
gtgggttgagaaacatcctttggaccaatgacccgctgctccgtgtagggcagagaaaggcccccctacccaggcccaccctccccagtccccaggcctccagccctggctctgcatcctttagagaagt
ggctgtctaatggggtccccacccgggaactgacagtaaggcagattctcagccccactgagacctccttagtcagactttggggtgggacccaagcatttgtattttctcaaggcctccagtgctgaagt
tgtgacaccctgactcgagtcatggtcttgaggccatgctccagtccatctttgcccagatgggcttgtgacccttggaagccccttgtcatctctgggatcagtttccccatatgttgtgggtttaggaa
ttcaaccacatgccgcaaagatcaccgccagaggatgatctggggccaaagttcctttggtggctcagtttggggggttgttcaggtggggagagagtgatcttctcttgacccttgctcaatcccacaagt
aatctcttccattgtggttcacagcttcagagtgtcagggctagacgacctcggcctcccaaacctcggcctcctcacaggccaaggccaaggccacggccatggttcccgccaaggttcccgggaaaacg
gtga

Beatragus hunteri

METQGASLSLGRCSLWLLLLGLALPSASAQAPSYREAVLRAVDRINDGSTEANLYRLLELDPPPKDVEDREARKPVSFRVKETVCPRPSQQPLEQCDFKENGLVKQCLGTVSLDPSDDQFDINCNELQSVR
DRRRRPPRPRPPHRPRPRPLPWFPPRFPGKR

GenBank: PVKQ01021889.1

atggagacccaggggccagcctctctctggggcgctgttcactgtggctcctgctgctgggactagcgctgccctcggccagcgcccaggcccccagctacagggaggccgtgcttcgcgctgtggatcg
catcaatgatgggtccacagaagctaatctctaccgcctcctggagcttgacccgcctcccaaggacgtgagttggggagggtgctgaatcatcttcccccagccttggccacactgtcgcccccttcggtc
aggctggtcctcctgtcaggaaggcacttttcctctaggtgggttaaccttcccagacctggggcccctcccagcaccaggcttcctgccttagcatctctgctgtgggatcaggcaccctgcacacctgg
ctccagctccctggacttctgggagctctagagatggagggatcacaggctctgtgaggtgacttccctgctaatgtcccccctgctcctcggtgtctccctgccaggagggcctctgccagcctggaggct
ccggtgacgagggctctccctacaggcggccctgacctccctgggcccttctgaggggaggcgctgccagcagcgctgctgtgagggccgctcctgctctctgtgtgcccgtgaggccgggcacgggctct
gtgccctcccctgtgctcccagcaccaagcccagggctggacacacagcgggctggagaggctgctgtctgggtgggggcagggagacagatcagagaaggaaacatgagcccaagcccagtctccccat
tttgatcgttgaccaggtggaggaccgggaagctcgaaagcctgtgagcttcagggtgaaggagactgtgtgccccaggccgagccagcagcccctggagcagtgtgacttcaaggagaatggggtgagcc
tgggggcagggagtgagggctgggatcaatgcttctcagtgctagctgaacagggaacttcagggacgatttccagcccctggggggtgaggtgggctgagcctgggaggttatggcccagggtttccagt
ttgacctggaaactccccttccagttggtgaaacagtgtttggggacagtcagcctggacccgtccgatgaccagtttgacataaactgtaatgaggtgagtggccccttctgtattgggcatatgctaac
aaggtgggttgagaaacatcctttggaccaatgacccgctgctccgtggagggcagagaaaaggccctcctacctgggcccactctccccagtccccaggcctccagccctggctctgcaccccctaagaga
agtggctgtctaatggggggccccacccgggaactgacagtaaggcagattctcagccccactgagacctcctgaatcagactttggggtgggacccaggcatttgtattttctcaaggcctccaggggctt
ctgacagtgctgaagttgtgacaccctgactcgagtcatggtctggaggccatgctctagtccatctttgcccagatgggctgtgacccttggaagccccttgtcatctctgggatcagtttccccatatg
ttgtgggtttaggaattcaaccacatgccccaaagatcaccgccagaggatgatctggagccaaagttcctttggtggctcagtttggggggttgttcaggtggggagagagtggtcttctcttgacccttg
cccaatcccacaagtaatctcttccattgtggttcacagcttcagagtgtcagggatagacgacgtcggcctcccagacctcggcctcctcacaggccaaggccaaggccactgccatggttcccaccaag
gttcccgggaaaacggtga

Damaliscus lunatus

METQGASLSLGRCSLWLLLLGLALPSASAQAPSYREAVLRAVDRINDGSTEANLYRLLELDPPPKDVEDREARKPVSFRVKETVCPRPSQQPLEQCDFKENGLVKQCLGTVSLDPSDDQFDINCNELQSVR
DRRRRPPRPRPPHRPRPRPLPWFPPRFPGKR

GenBank: SJXP01002955.1

atggagacccaggggccagcctctcgctggggcgctgttcactgtggctcctgctgctgggactagcgctgccctcggccagcgcccaggcccccagctacagggaggccgtgcttcgcgctgtggatcg
catcaatgatgggtccacagaagctaatctctaccgcctcctggagcttgacccgcctcccaaggacgtgagttggggagggtgctgaatcatcttcccccagccttggccacactgtcgccccttcggtc
aggctggtcctcctgtcaggaaggcacttttcctctaggtgggttaaccttcccagacctggggcccctcccagcaccaggcttcctgccttagcatctctgctgtgggatcaggcaccctgcacacctgg
ctccagctccctggacttctgggagctctagagatggagggatcacaggctctgtgaggtgacttccctgctaatgtcccccctgctcctcggtgtctccctgccaggagggcctctgccagcctggaggct
tcggtgacgagggctctccctgcaggcggccctgacctccctcggcccctctgagggaggcgctgccagcagcgctgctgtgagggccgctcctgctctctgtgtgcccgtgaggccgggcacgggctct
gtgccctcccctgtgctcccagcaccaagcccagggccggacacacagcgggctggagaggctgctgtctgggtgggggcagggagacagatcagagaaggaaacatgagcccaagcccagtctccccat
tttgatcgttgaccaggtggaggaccgggaagctcgaaagcctgtgagcttcagggtgaaggagactgtgtgccccaggccgagccagcagcccctggagcagtgtgacttcaaggagaatggggtgagcc
tggggcagggagtgagggctgggatcaatgcttctcagtgctagctgaagggtgaggtgggctgagcctgggaggttatggcccagggtttccagtttgacctggaaactccccttccagttggtgaaac
agtgtttggggacagtcagcctggacccgtccgatgaccagtttgacataaactgtaatgaggtgagtggccccttctgtattgggcatatgctaacacggtgggttgagaaacatcctttggaccaatga
cccgctgctccgtgtagggcagagaaaggccctcctacctgggcccactctccccagtccccaggcctccagccctggctctgcaccccctaagagaagtggctgtctaatggggtccccacccgggaact
gacagtaaggcagattctcagccccactgagacctcctgaatcagactttggggtgggacccaggcatttgtatttctcaaggcctccaggggcttctgacagtgctgaagttgtgacaccctgactcga
gtcatggtctggaggccatgctctagtccatctttgcccagatgggctgtgacccttggaagccccttgtcatctctgggatcagtttccccatatgttgtgggtttaggaattcaaccacatgccccaaa
gatcaccgccagaggatgatctggagccaaagttcctttggtggctcagtttggggttgttcaggtggggagagagtggtcttctcttgacccttgcccaatcccacaagtaatctcttccattgtggtt
cacagcttcagagtgtcagggatagacgacgtcggcctcccagacctcggcctcctcacaggccaaggccaaggccactgccatggttcccaccaaggttcccgggaaaacggtga

Moschus moschiferus

METQRASISLGRWSLWLLLLGLVLPSASAQGLSYREAVLRAVDQLNDGSTEANLYRLLELDPPPKDVEDQGARTPASFTVKETVCPKTSQQPPEQCDFKENGLVKQCVGTVSLDRSDDQSDINCNELQSVR
AKRPRPPKPRRPHRPRPRPLPWFPPRFPGKR

GenBank: PVHU021072425.1

atggagacccagagggccagtatttccctggggcggtggtcactgtggctactgttgctgggactagtgctgccctcggccagcgcccagggcctcagctacagggaggccgtgcttcgtgctgtggatca
gctcaatgacgggtccacagaagctaatctctaccgcctcctggagctagacccacctcccaaggacgtgagttggggaggggctgggccatctccccccaactttggccacactgtctctcccttcgct
ctggctgtatctccagtcaggaaggcacttttccctctccgtgggctcttacctcttccaggaaaccttcccagacctgggtccctcccagcaccaggcttcctgccttagcatctctgctgtgggaacag
gcgccctgcacacctggctcaggctccctggccttctgggagctccagggatggaggggtcacaggctctgtgaggtgacttccctgctaaagtcccctctgcacctcggtgtctccctaccagggaagcc
tctgtcagtctggaggttccagtgacagggctctccctgcaggcggccctgacccccctcagcccctccgagggaggcgctgccatcattgctggtgtgaggtccgctcctgctctctgtgtgcccgtga
ggctggggacggctctgtcccctcccctgcgctcccagcaccaagcccagggccggacacacaaggggctggagaggctgctgtctgggttggggcacggaaacagatcagagaaggaaagaagacccca
ggcccagtctccccactttatctttgaccaggtggaagaccagggagctcgaacacctgcaagcttcacggtgaaggaaactgtgtgccccaagacgagccagcagcccccagagcaatgtgacttcaag
gagaatggggtaagcctgtgggctgagactgagggctgggaataaaagcttctcagtgcaagctgaacagggaactttggggaagatttccagcccctgagggcgaggtgggctgagcctaggaggttat
ggcccggagtttccagtttgacctggaaattccccttccagctggtgaagcagtgtgtggggacagtcagcctggaccggtccgatgaccaatctgacataaactgtaatgaggtgagtggccccttctgt
gttgggcatatgctaacagggtgggttgaggaacatcctttggaccaatgacccgctgctccatctagggcagagaaaaggccctcctacccaggcccaccttcctcaatccccaggtctccagccctggc
tctacatcctttagagaagtggctgtctaacggggtccccacccaggaactgacagtaaggcaggttctcagccccactgagacctcctgaatcagactttggggtgtggcccaggcatttgtattttcac
aaggtcttcaggggatgctgacagtgttgaagttgtgacaccctgactcaagtcacggtcttgaggccatgctccagtccatctttgcctggatgggcttgtgactcttggaagccccttgtcatctctgg
gatcagtttcctcatatgttgtgggtttagggattcaaccacatgctccaaagatcactgccagaggatgagctggggccaaagttcctttggtggcccagtttgggggttgttcaggtggcgagagagtg
gtcttctcttgatccttgcccagtcccacaagtgattgcttccattgtggttcacagctccagagtgtcagggctaaacgacctcggcctcccaaacctcggcgtcctcacaggccaaggccaaggccact
gccatggttcccgccaaggttccctggaaaacggtga

Moschus berezovskii

METQRASISLGRWSLWLLLLGLVLPSASAQALSYREAVLRAVDQLNEKSSEANLYRLLELDPPPKDVEDQGARTPASFTVKETVCPKTSQQPPEQCDFKENGLVKQCVGTVSLDRSDDQSDINCNELQSVR
AKRPRPPKPRRPHRPRPRPRPWFPPRFPGKR

GenBank: SGQJ01039619.1

atggagacccagagggccagtatttccctggggcggtggtcactgtggctactgttgctgggattagtgctgccctcggccagcgcccaggccctcagctacagggaggccgtgcttcgtgctgtggatca
gctcaatgagaagtcctcagaagctaatctctaccgcctcctggagctagacccacctcccaaggacgtgagttggggaggggctgggccatctccccccaactttggccacactgtccctccctttgct
ctggctgtatctccagtcaggaaggcacttttccctctccgtgggctctcacctcttccaggaaaccttcccagacctgggtcccctcccagcaccaggcttcctgccttagcatctctgctgtgggaaca
gacgccctgcacacctggctcaggctccctggccttctgggagctccagggatggaggggtcactggctctgtgaggtgacttccctgctaaagtcccctctgcacctcggtgtctccctaccagggaagc
ctctgtcagtctggaggttccagtgacagggctctccctgcaggcggccctgacccccctcagcccctccgagggaggcgctgccatcattgctggtgtgaggtccgctcctgctctctgtgtgcctgtg
aggccagggacgggctctgtcccctcccctgcgctcccagcaccaagcccagggccggacacacaaggggctggagaggctgctgtctgggtgggggcacggaaacagatcagagaaggaaagaagacccc
aggcccagtctccccacttttatctttgaccaggtggaagaccagggagctcgaacacctgcaagcttcacggtgaaggaaactgtgtgccccaagacgagccagcagcccccagagcaatgtgacttcaa
ggagaatggggtaagcctgtgggctgagactgagggctgggaataaaagcttctcagtgcaagctgaacagggaactttggggaagatttccagcccctgaggggcgaggtgggctgagcctaggaggtta
tggcccggagtttccagtttgacctggaaattccccttccagctggtgaagcagtgtgtggggacagtcagcctggaccggtccgatgaccaatctgacataaactgtaatgaggtgagtggccccttctg
tgttgggcatatgctaacagggtgggttgaggaacatcctttggaccaatgacccgctgctccatctagggcagagaaaaggccctcctacccaggcccaccttcctcaatccccaggtctccagccctgg
ctctacagcctttagagaagtggctgtctaacggggtccccacccaggaactgacagtaaggcaggttctcagccccactgagacctcctgaatcagactttggggtgtggcccaggcatttgtattttca
caaggtcttcaggggattctgacagtgttgaagttgtgacaccctgactcaagtcacggtcttgaggccatgctccagtccatctttgcctggatgggcttgtgactcttggaagccccttgtcatctctg
ggatcagtttccccatatgttgtgggtttagggattcaaccacatgctccaaagatcactgccagaggatgagctggggccaaagttcctttggtggcccagtttggggggttcaggtggcgagagagtggt
cttctcttgatccttgcccagtcccacaagtgattgcttccattgtggttcacagctccagagtgtcagggctaaacgacctcggcctcccaaacctcggcgtcctcacaggccaaggccaaggcctcggc
catggttcccgccaaggttccctggaaaacggtga

Moschus chrysogaster

METQRASISLGRWSLWLLLLGLVLPSASAQALSYREAVLRAVDQLNEKSSEANLYRLLELDPPPKDVEDQGARTPASFTVKETVCPKTSQQPPEQCDFKENGLVKQCVGTVSLDRSDDQSDINCNELQSVR
AKRPRPPKPRRPHRPRPRPRPWFPPRFPGKR

GenBank: SPDY01043557.1

atggagacccagagggccagtatttccctggggcggtggtcactgtggctactgttgctgggattagtgctgccctcggccagcgcccaggccctcagctacagggaggccgtgcttcgtgctgtggatca
gctcaatgagaagtcctcagaagctaatctctaccgcctcctggagctagacccacctcccaaggacgtgagttggggaggggctgggccatctccccccaactttggccacactgtccctccctttgct
ctggctgtatctccagtcaggaaggcacttttccctctccgtgggctctcacctcttccaggaaaccttcccagacctgggtcccctcccagcaccaggcttcctgccttagcatctctgctgtgggaaca
gacgccctgcacacctggctcaggctccctggccttctgggagctccagggatggaggggtcactggctctgtgaggtgacttccctgctaaagtcccctctgcacctcggtgtctccctaccagggaagc
ctctgtcagtctggaggttccagtgacagggctctccctgcaggcggccctgacccccctcagcccctccgagggggaggcgctgccatcagcactggtgtgaggtccgctcctgctctctgtgtgcccgtg
aggccagggacgggctctgtcccctcccctgcgctcccagcaccaagcccagggccggacacacaaggggctggagaggctgctgtctgggtgggggcacggaaacagatcagagaaggaaagaagacccc
aggcccagtctccccactttatctttgaccaggtggaagaccagggagctcgaacacctgcaagcttcacggtgaaggaaactgtgtgccccaagacgagccagcagcccccagagcaatgtgacttcaa
ggagaatggggtaagcctgtgggctgagactgagggctgggaataaaagcttctcagtgcaagctgaacagggaactttggggaagatttccagcccctgagggcgaggtgggctgagcctaggaggtta
tggcccggagtttccagtttgacctggaaattccccttccagctggtgaagcagtgtgtggggacagtcagcctggaccggtccgatgaccaatctgacataaactgtaatgaggtgagtggccccttctg
tgttgggcatatgctaacagggtgggttgaggaacatcctttggaccaatgacccgctgctccatctagggcagagaaaaggccctcctacccaggcccaccttcctcaatccccaggtctccagccctgg
ctctacatcctttagagaagtggctgtctaacggggtccccacccaggaactgacagtaaggcaggttctcagccccactgagacctcctgaatcagactttggggtgtggcccaggcatttgtattttca
caaggtcttcaggggattctgacagtgttgaagttgtgacaccctgactcaagtcacggtcttgaggccatgctccagtccatctttgcctggatgggcttgtgactcttggaagccccttgtcatctctg
ggatcagtttccccatatgttgtgggtttagggattcaaccacatgctccaaagatcactgccagaggatgagctggggccaaagttcctttggtggcccagtttggggggttcaggtggcgagagagtggt
cttctcttgatccttgcccagtcccacaagtgattgcttccattgtggttcacagctccagagtgtcagggctaaacgacctcggcctcccaaacctcggcgtcctcacaggccaaggccaaggcctcggc
catggttcccgccaaggttccctggaaaacggtga

Antilocapra americana

MGTQRASLSLGRWSLWLLLLGLLLPSASAQALSYREAVLRAVDRINDGSTEANLYRLLELDPPPKDVEDRGARKPASFTVKETVCSRTSQQPPEQCDFQENGLVKQCVGTVSLDQSDEQFDINCNELQSVR
IKIPRPPKPRRPHRPRPGRRRPWFPPRFPGKR

GenBank: PVKT010018561.1

atggggacccagagggccagcctctccctgggacggtggtcactgtggctactgctgctcggactactgctgccctcggccagcgcccaggccctcagctacagggaggccgtgcttcgcgctgtggatcg
catcaatgatgggtccacagaagctaatctctaccgcctcctggagctagacccgcctcccaaggacgtgagttgaggaggggcttggccatctcccccaagccttggccacactgtccctcccttcact
caggctgtacctcctgtcaggaaggctcttctccctctaggtgggctcccacctcttccaggaaatcttcccacacctgggaccctcccagcaccaggcttcctgccttagcatctctgctgtgggaacat
gtaccctgcacacctggctcaggctccctggacttctgggagctccagggatagagagggtcattggttctatgaggtgacttccctgctaatgtcccctctgtaccttggtgtctccctaccagggagga
ctctgtcagcctggaggttcctgtgacaagggctctccctgcaggcggccccgacctccctcagcccctctgaggggaggagctgccatcagcgctggtgtgaggtctgctcctgcgggctctgtcccct
cccctgtgctcctgggaccaagcccagggccagacacacagcgggctggagaggctgccgtctgggtgagggcagcgagacagatcagagaaggaaacatgagcctgagcccagtctccccattttgatc
tttgaccaggtggaggaccggggagctcgaaagcctgcaagcttcacggtgaaggagaccgtgtgctccaggacgagccagcagcctccggagcagtgtgacttccaggagaatggggtgagccgaggggg
ttgagactgatggctgggataaatgcttctcagtgcaagctgaacagggaatttcggggaagatttccagcccctggagggtgagatggctgagcttgggaggttatggcccaggggttccagtttgacc
cggaaactccccttccagctggtgaaacagtgtgtggggacagtcagcctggaccagtccgatgagcaatttgacataaactgtaatgaggtgagtggtcccttctgtttgtggagatggtgagttgtgga
acatcctttggaccaatgacccactgccccatccagggcagagaaaagaccctcctacctgggcccacctccccaatctccaggtgtccagctctggttctgcatcctttagagaatggctgtctaatggg
gtccccaccagggaactgacaggcagattctcagccccactgaaacctcctgaatcagactttgaggtggggcccaggcatttgtattttcacaaggcctccaggggattctgacagtactcaaattgtgc
cacattgattcaagtcatggtcttgaggccatgctccagtccatctttgcccggatgggcttgtgacacttggaagccccttgtcatctctgggatcagtttccccatatgttgtgggtctaggattcaa
caacatgctccaaagatcactgccagaggatgatctggggccaaagttcctttggtggcccagtttggggattgttcaggtcaggagagagtgatcttctcttgacccttgcccagccccacaagtaatct
gtttcattgtggttcacagcttcagagtgtcaggattaaatacctcggcctcctaagcctcgacgtcctcacaggccaaggccagggcgacgacggccatggttcccgccaaggttccccggaaaacggt
ga

Syncerus caffer

MKTQRASLSLGRWSLWLLLLGLVLPSASAQALSYREAVLRAVDRINDGSTEANLYRLLELDPPPKDVEDRGARKPVSFRVKETVCPRPSLQPPEQCDFKENGLVKQCLGTVSLDRSDDQFDINCNELQSVR
AKRPRPPKPRPPHRPRPRPRPWFPPRFPGKR

GenBank: SJXX01000493.1

atgaagacccagagggccagcctctccctagggcgctggtcgctgtggctactgctgctgggactggtgctgccctcggccagtgcccaggctctcagctacagggaggccgtgcttcgtgctgtggatcg
catcaatgatgggtccacagaagctaatctctaccgcctcctggagctagacccgcctcccaaggatgtgagttggagaggggctgagccatctcctcccagctttggccacactgtcgccgcttcactaa
ggctggttctcttgtcaggaaggcacttttccctctaggtgggctcccatctcttccaggaaaccttcctagacctggggccctcccagcaccaggcttgctgccttagcatctctgctgtgggaacaggc
aacctgcacacctggctcaggctccctggacttctgggagctccagggatggaggggtcacaggctctgtgaggtgacttccctgctaatgtcccctctgcacctcggtgtctccctaccagggaggcctc
tgtcagcctggaggctccagtgacaagggctctccctgcaggcggccctgacctccctcagcccctgtgagggaggcgctgccatcagcgctgctgtgagggccgctcctgctctctgtgtgccggtgag
gccgggcatgggctctgtcccctcccctgtgctcccggcaccaagcccagggccggacacacagtgggctggagaggctgcggtccgggtggagggcaaggagacagatcagagaaggaaacatgagcctg
agtccagtctccgcactttgatccttgaccaggtggaggaccggggagctcgaaagcctgtgagcttcagggtgaaggagactgtgtgccccaggccgagcctgcagcccccggagcagtgtgacttcaag
gagaatggggtgagcctggggactgagactgagggctgggatcaatgcttctcagcgtgagctgaacagggacttcagggaatgtttccagcccctggcaggcgaggtgggctgagcctgggagattatgc
ccggggtttccagtttgaccttgagctccccttccagctggtgaaacagtgtttggggacagtcagcctggaccggtccgatgaccagtttgacataaactgtaatgaggtgagtggccccttctgtgttg
ggcatatgctaacagggtgggttgagaaacatcctttggaccaatgacccgctgctccatctagggcagagaaaaggccctcctatctgggcccaccctccccaatccccaggtctccagccctggctcta
catcctttagagaagtggctgctaatggggtccacacccaggaactcacagtaaggcagattctcagccccactgagacctcctgaatcagactttggggtggggccccggcatttgcattttcacaaggc
ctccagcggattctgacggggctgaaattgtgacaccctgacttgagtcatggtcttgaggccatgctccagtccatctttgcccggatgggcttgtgacccttggaagcccttgtcatctctgggatcag
tttccccatatgttgtgggtttagggattcaactacatgttccaaagatcactgccagaggattatctggggccaaagttcctttggtggctcagttcgggggttgttcatgtggaagagagtggtcttc
tcttgacccttgcccagtcccacaagtaatctcttccattgtggttcacagcttcagagtgtcagggctaagcgacctcggcctcccaaacctcggcctcctcacaggccaaggccaaggccacggccatg
gttcccgccaaggttccccggaaaacggtga

Raphicerus campestris (2 gene copies)

METQRARLLLGRCSLWLLLLGLVLPLASTQALSYREAVLRAVDRINDGSSEANLYRLLELDLPPKDVEDRGARKPVSFRVKETVCPRTTQQPLEQCDFKENGLVKQCLGTVSLDPSDDQFDINCDELQSVR
SRRRPPKPRPPHRPRPRPRPWFPPRFPGKR

GenBank: SJYW01011678.1

atggagacccagagggccaggctcttgctgggacggtgttcgctgtggctcctgctgctgggactagtgctgcccttggccagcacccaggccctcagctacagagaggccgtgcttcgtgctgtggatcg
catcaatgatgggtcttcagaagctaatctctaccgcctcctggagcttgacctgcctcccaaggacgtgagttggggaggggctgagccatctccgcccagccttggccacactgtcgccccttcgctc
aggctggtcctcctgtcaggaaggcactttcccgctaggcaggctcccacctcttccaggaaccttcccagacctgggcccctcccagcaccaggcttcctgccttagcatctctgctgtgggaacagg
cgccctgcatacttggctcaggctcccgggacttctgggagctccagggacagaggggtcacaggctctgtgaggtgacttccctgctaatgccccctgcacctcggtgtctccatgccaggagggcctt
tgtcagcctggaggctccagtgaccagggctctccctgcaggcggccctgacggccctgggccactctgaggggaggcgctgccagcagcgctgctgtgagggccgctcctgctctctgtgtgcccgtgag
gccgggcgcgggctctgtgccctcccctgtgctcccagcaccaagccaagggctggacacacaggggggctggagaggctgccatccgggtgggggcagggagacagatcggagaaggaaacacgagcccg
agcccagtctccccactttgatcgttgaccaggttgaggaccggggagctcgaaagcctgtgagcttcagggtgaaggagactgtgtgccccaggacgacccagcagcccctggagcaatgtgacttcaag
gagaatggggtgagcctggggcggggactgagggctgggatcagtgcttctcagtgccagctgaacagggaacttcagggacgatttctagcccctagagggtgaggtgggctgagcctgggaggttatg
gccctgggtttccagattgacctggaaactacccttgcagctggtgaaacagtgtttggggacagtcagcctggacccgtccgatgaccagtttgacataaactgtgatgaggtgagtggccccttctgtg
ttgggcatatgctaaaaaggtgggttgagaaacatcctttggaccaatgacccgctgctctgtctagggcagagagaaggccctcttacctgggcccaccctccccagtccccaggtctccagccctggc
cttgcatcccttacagaagtgtctgtctaatggggtccccactcaggaactgacagtaaggcagattctaagccccactgagacctcccgaatcagactttggggtgggacccaggcatttgtattttcac
aagacctccagtggattctgacagtgctgaagttgtgacaccctgactcgagtcatggtcttgaggccatgctccagtccatctttgcccacatgggcttgtgacccttggaagccccttgtcatctctgg
gatcagtttccccatatgttgtgggtttaggaattcaaccacatgccccaaagatcactgccagaggatgatctggggccaaagttcctttggtggctcagtttggggttgttcaggtggggagagagtg
gtcttctcttgacccttgcccaatcccacaagtaatctcttccactgtggttcacagcttcagagtgtcaggtctagacgacggcctcccaaacctcggcctcctcacaggccacggccaaggccacggcc
atggttcccgccaaggttccccggaaaacggtgaaggactggctgtcacacctattaatggcttttggtgaattccgagcctgagggaagcatttaaagatatgatttgttctggctcagacttctggac
ggtgaaa**aataa**attcttgtgaaaacaacttcctccaggcttcagtttcaattatttcccttttccccatttactgagactgagtccttaactctgagaggcctactctgtgtgtgtgtgt**/gap531bp/**
ctccctgcaggcggccctgacggccctgggccactctgaggggaggcgctgccagcagcgctgctgtgagggccgctcctgctctctgtgtgcccgtgaggccgggcgcgggctctgtgccctcccctgtg
ctcccagcaccaagccaagggctggacacacaggggggctggagaggctgccatccgggtgggggcagggagacagatcagagaaggaaacacgagcccgagcccagtctccccactttgatcgttgacca
ggttgaggaccggggagctcgaaagcctgtgagcttcagggtgaaggagactgtgtgccccaggacgacccagcagcccctggagcaatgtgacttcaaggagaatggggtgagcctggggcggggactg
agggctgggatcagtgcttctcagtgccagctgaacagggaacttcagggacgatttctagcccctagagggtgaggtgggctgagcctgggaggttatggccctgggtttccagattgacctggaaacta
cccttgcagctggtgaaacagtgtttggggacagtcagcctggacccgtccgatgaccagtttgacataaactgtgatgaggtgagtggccccttctgtgttgggcatatgctaaaaaggtgggttgaga
aacatcctttggaccaatgacccgctgctctgtctagggcagagagaaggccctcctacctgggcccaccctccccagtccccaggtctccagccctggccttgcatcccttacagaagtgtctgtctaat
ggggtccccactcaggaactgacagtaaggcagattctaagccccactgagacctcccgaatcagactttggggtgggacccaggcatttgtattttcacaagacctccagtggattctgacagtgctgaa
gttgtgacaccctgactcgagtcatggtcttgaggccatgctccagtccatctttgcccacatgggcttgtgacccttggaagccccttgtcatctctgggatcagtttccccatatgttgtgggtttagg
aattcaaccacatgccccaaagatcactgccagaggatgatctggggccaaagttcctttggtggctcagtttggggttgttcaggtggggagagagtggtcttctcttgacccttgcccaatcccacaa
gtaatctcttccactgtggttcacagcttcagagtgtcaggtctagacgacggcctcccaaacctcggcctcctcacaggccacggccaaggccacggccatggttcccgccaaggttccccggaaaacgg
tga

Procapra przewalskii

METHRASRALGRCSLWLLLLGLVMPSASAQTPSYREAVLRAVDRINDGSTEANLYRLLELDPPPKDVEDRGARKPVSFRVKETVCPRTSQQPAEQCDFKENGLVKQCLGTVSLDPSDDQFDINCNELQSVR
SRRRPPKPRRPHRPRPRPLPWFPPRFPGKR

GenBank: SJYF01000158.1

atggagacccacagggccagccgcgctctgggacggtgttcgctgtggctcctgctgctgggactagtgatgccctcggccagtgctcagacccccagctacagagaggccgtgcttcgtgctgtggatcg
cattaatgatggatccacagaagctaatctctaccgcctcctggagctagacccgcctcccaaggacgtaagttggggaggggctgagccatctccctccagccttggccacactgtcaccccttcgctc
aggctggtcctcctgtcaggaaggcacttttccctctaggcgggctcccacctcttccaggagaccttcccagacctgggtcatctcccagcaccaggcttcctgccttagcatctctgctgtgggatcag
gcgccctgcacacctggctcaggc/gap65bp/ccttgcacctcggtgtctccctgccaggagggcctctgtcagcctggaggctccagtgacaagggctctccctgcaggcggccctgacctccctcggc
ccttctgaggggaggcgctgccagcagcgctgctgtgagggccgctcctgctctctgtgtgctcgtgaggccgggcacgggctctgtgccctcccctgtgctcccagcaccaagcccagggccagacacac
aggggggctggagaggctgccgtccgggtgggggcagggagacagatcagagaagggaacatgagccgagctcagtctccccactttgatcgttgaccaggtggaggaccggggcgctcgaaagcctgtga
gcttcagagtgaaggagactgtgtgccccaggacgagccagcagcccgcggagcaatgtgacttcaaggagaatggggtgagtctggcggtgcagagtgagggctgggatcagtgcttctcagtgcaaact
caacagggaacttcagggacgatttccagccactggggggtgaggtgggctgagcctgggaggttatggccctgggtttccagtttgacctagaaactacccttccagctggtgaaacagtgcttggggac
agtcagcctggacccgtccgatgaccagtttgacataaactgtaatgaggtgagtggccccttctgtgttgggcatatgctaaaaaaggtgggttgagaaacatcctttggaccaatgacccgctgcccgg
tctagggcagagagaaggccctcctacctgggcccaccctccccagtccccaggtctccagccctggctctgcatcccttaaagaagtgtctgtctaatggggtccccacccaggaactgacagtaaggca
gattctcagccccactgagacctcctgaatcagactttggggtgggacccaggcatttgtattttcacaagacctccaggggactctgacagtgctgaagttgtgacaccctgactcgagtcatggtcttg
aggccatgctccagtccatctttgcccacatgggcttgtgacccttggaagccccttgtcatctctgggatcagtttccccatatgttgtgggtttaggaattcaaccacgtgccccaaagatcactgcca
gaggatgacctggggccaaagttcctttggtggctcagttaggggggttgttcaggtggggagagagtggtcttctcttgacccttgcccaatcccacaagtaatctcttccattgtggttcacagcttcag
agtgtcaggtctagacgacggcctcccaaacctcggcgtcctcacaggccacgcccaaggccactgccatggttcccgccaaggttccccggaaaacggtga

Madoqua kirkii

METQRARLSLGRCSLWLLLLGLALPSASAQALSYREAVLRAVDRINDGSSEANLYRLLELDPPPKDDENPNIPKPVSFRVKETVCPRTTQQPLEQCDFKENGLVKQCVGTVSLDLSDDQFDINCNELQSVR
SRRRPPKPRPPHRPRPRPLPWFPPRFPGKR

GenBank: SJYC01115865.1

atggagacccagagggccaggctctccctgggacggtgttcgctgtggctcctgctgctgggactagcgctgccctcggccagcgcccaggccctcagctacagggaggccgtgcttcgtgctgtggatcg
catcaatgatgggtcctcagaagctaatctctaccgcctcctggagcttgacccgcctcccaaggacgtgagttggggagggagctgagccatctccggccagccttggccacactgtcaccgcttcgctc
aggctggtcctcctgtcaggaaggcacttttcctctaggcgggctctcacctcttccagaaaccttcccagtcctgggtcatctcccagcaccaggcttcctgccttagcatctctgctgtgggaacagg
caccctgcacacctggctcaggctcccgggacttctgggagctccagggatggaggggtcacaggctctgtgaggtgacttccctgctaatgcccctgcacctctgtgtcccccctgccaggagggcctctg
tcagcctggaggctccggtgacaagggctctccctgcaggtggccctgacctccctgggcccctctgagggggaggcgctgccagcagcgctgctgtgagggcctctcctgctctctgtgtgcccgtgaggc
cgggcacaggctctgtgccctcccctgggctccctgaaccaagcccagggccggacacacaggggggctggagaggctgccgtccaggtgggggcagggagacagatcagagaaggaaacgtgagctgagc
ccagtctccccatttttattcctggatcaggatgagaatccaaacatcccgaagcctgtgagcttcagagtgaaggagactgtgtgccccaggacgacccagcagcccctggagcaatgtgacttcaaggag
aatggggtgagcctggggggtggggactgagggctgggatcagtgtttctcagtgcaagctgaacagggaacttctgggaggatttccagcccctgggtgtgaggtgggctgagcctgggaggttatggcc
ctgggtttccagtttgacctggaaactacctttccagctggtgaaacagtgtgtggggacagtcagcctggacctgtccgatgaccagtttgacataaactgtaatgaggtgagtggccccttctgtgttg
ggcatatgctaaaaaaagttgggttgagaaacatcctttggaccaatgacccgctgctctgtctagggcagagagaaggccctcctacgtgggcccaccctccccagtccccaggtctccagccctggctc
tgcatcccttacagaagtgtctgtctaatggggtccccacccaggaactgacagtaaggcaggtgctcagccccactgagacctgaatcagactttggggtgggacccaggcatttgtattttcacaagac
ctccagcggattctgacagtgctgaagttgtgacactctgacttgagtcatggtcttgaggccatgctccagtccatctttgcccacatgggcttgtgacccttggaagccccttgtcatctgggatcagt
ttccccatatgttgtgggtttaggaattcaaccacatgccccaaagatcactgccagaggatgatctggggccaaagttcctttggtggctcagtttgggggttgttcaggtggggagagagtggtcttct
cttgatccttgcccaatcccacaagtaatctcttccattgtggttcacagcttcagagtgtcaggtctagacgacggcctcccaaacctcggcctcctcacaggccacggccaaggccactgccatggttc
ccgccaaggttccctggaaaacggtga

Oreotragus oreotragus

METQRASLSLGRWSLWLLLLGPVLPSASAQALSYREAVLRAVDQLNEKSSEANLYRLLELDPPPRDVEDRGARKPVSFRVKETVCPRQSQQPPEQCDFKENGLVQQCVGTVSLDPSDDQFDLNCNELQSVR
AIRPWPPKPRPPHRPRPRPRPWFPPRFPGKR

GenBank: SJYX01024341.1

atggagacccagagggccagcctctcgctggggcggtggtcactgtggctcctgctgctgggaccagtgctgccctcggccagcgcccaggccctcagctacagggaggccgtgcttcgtgctgtggatca
gctcaatgagaaatcctcagaagctaatctctaccgcctcctggagctagacccacctcccagggacgtgagttggggaggggactagagatgggcccatctcccgcctgccttggccacactgtcgcccc
ttcgctcaggctggtcctcctgtcaggaaggcacttttccctctaggcggctcccacctcttccaggagaccttcccaggcctgggcccctcccggcaccaggcttcctgccttagcgtctctgctctggg
ctcaggcgccctgcacacctggctcaggctgccggggctcctgggagctccaggggtggaggggtcacaggctctgtgaggtgacctccctgcggatgtcccccctgcacctcggtgtctccctgccagggg
ggcctctgtcagcctggaggctccagtgacaggggctctccctgcaggcggccctgaccgccctgggcccctctgagggaggcgctgccagcagcgctgcagtgagggccgcccctgctctctgtgtgcc
ctgaggccgggcccgggctccgtgccctcgcctgtgctcccagcaccaagcccagggccggacacacaggggggctggagaggctgccgtccgggtgggggcagggagacgggtcagagaagggaacatga
gccgagcccagtctccccactttgattgttgaccaggtggaggaccggggagctcgaaagcctgtgagcttccgggtgaaggagaccgtgtgccccaggcagagccagcagcccccggagcagtgtgactt
caaggagaacggggtgagcctgagggccaggagtgagagctgggatcaatgcttctcagcacgagttgaacaaggaacttcgggaaggtttccagcatgtaggggggtgaggtgagcctgagaggttatggc
ccggggggttccagattgaccttgagctccccttgcagctggtgcaacagtgtgtggggacagtcagcctggacccgtccgatgaccagtttgacctaaactgtaatgaggtgagtggccctttctggtttt
ggaggattctaggggggtagagtgtggagcatgctttggatcgatgacccgctgccccatccagggtagagaaaggccctcctacctgcacccctccctcccccaggtcccaggtctccagccttggctctg
catcccttagagcagtggttctctaatgcggtccccacctgggaacagacatgagacagattctcatgtctgagagctcctgaatcagactctggggtggggtccagctatttgtattttcacaaggcctg
caggggattctgactggctgaagttgagaggaattgactcagtaatgatctctaatccctgctctaatctggctttgctaggatggacttgtgacccttggaagccctatgttgtctgtggatctgtttc
tccctaggtgtctgtatatagggattcaatcatatgcttcaaagataacagccagagggtgaacagggtctaatacccgtggtgtcccagttagagggctgttcaggtgtgaagtgagggatcttgtcttg
acccttgcacaatcccacaacaaatctgtttttttcatggtttacagcttcagagtgtcaggagacttcgtccccggcgcccacgtttgccaaggccaaggccaaggccattgccatttccacggccagggc
caaggccaattccaaggccattgccatttccacggccagggccaaggccaattccaaggccattgccatttccagggccacagccaaggccaattccaaggcctttgtga

Redunca redunca

LENQGARKPVSFRVKETVCPRPSLQPVEQCDFKENGLVKQCLGTVSLDRSDDQFDINCNELQSVRAVRPRPPKPRRPHRPRPRPRPWFPPRFPGKR

GenBank: SJYQ01093930.1

contig_start_gggcctctgtcagcctggaggctccagggacaagggctctccctgcaggcggccctgacccccctcggcccctctgagcggaggcgctgccagcagcgctgctgtgaggccgctcct
gctctctgtgtgcccgcgaggccgggcatgggctctgtgccctcccctgtgctcccagcaccaagcccagggctgggcacacaaggggctggagaggctgccgtctgggtgggaggcagggagacaggtca
gagaaggaaacatgagcccgagccctgtctccccactttgatcattgaccagttggagaaccagggagctcgaaagcctgtgagcttcagggtgaaggagactgtgtgccccaggccgagcctgcagccgg
tggagcagtgtgacttcaaggagaatggggtgagcctggggtggggagtgagggctgggatcaatgcttctcagtgttagctgaacagggaacttcagggatgatttccagcccctggggatgaggtcg
gctgagcctgggaggttatggcccagggttccagtttgacccggaaactccccttccagctggtaaaacagtgtttggggacagtcagcctggaccggtctgatgaccagtttgac**ataaa**ctgcaatgag
gtgagtggccccttctgtgttgggcgtatgctaacaaggtgggttgagaaacatcctttggaccaatgacccgctgctctgtctggggcagagaaaaggtcctcctacccgggcccaccctccccagtccc
caggtctccagccttggctctgcgtcctttagagaattggctgtctaatggggtccccacccaggaagtgacagtaaggcagattctcagccccactgagaccttctgaatcagactttgggtgggacct
aggcatttgtattttcataaggcctccaggggattctgacagtgctgaagttgtgacaccctgactcgagtcatggtcttgaggccatgctccagcccatttttgcccagatgggcttgtgacccttggaa
gtcctttgtcatctctgggatcagtttccccataggttgtgggtttaggaattcaaccacatgccccaaagatcaccgccagaggatgatctggggccaaagttcctttggtggctcagtctggggttgt
tcaggtggggagagagtagtcttctcttgacccttgcctagtcccacgagtaatctcttccattgtggttcacagcttcagagtgtcagggctgtacgacctcggcctcccaaacctcggcgtcctcacag
gccaaggccaaggccacggccgtggttcccgccaaggttccccggaaaacggtga

Neotragus pygmaeus

DENPNIPKPVSFRVKETVCPRPSQQPAEQCDFKENGLVKQCLGTVSLDPSDDQFDINCNELQSVRAKRPRPPKSRRPHRPRRRPRPWFPPRFPGKR

GenBank: SJYV01040733.1

aacctctacctacattgttcaaaattcagtgtcccagaacttggcataataaatatagttgtttgattccttgttatgatatgaggcaaatagttattatcttcattttatagatgaagaatcatgcttcc
atatggctgcaaaattggccaaatagcagaagaactgagattcaagcccaaactgtgggactccaagacaccgtgaaaatcagacaggtagaaaagcaatgcgtgaactggaaagtgacagtctctgtctt
attctgtaacccttgttgccagtgaacactcccaggacaacccaggctaatgcacagttcctggaacacattttcattcaatggctttctccttaactgaagaaagtatgtgtgagagttggactgtgaag
aaggctgagcgccgaagaattgatgcttttgaactgtggtgttggnctgtcagcctgcaggctccggtgacaagggctcccccctgcaggcggccctggccgccctcagcccctctgagggaggcgctgcc
ggcagcgctgctgtgagggccgctgctgctctctgtgtgcccgtgaggccgggcacgggctctgtgccctcccctgtgctcccagcaccaagcccagggccagacacacagggggctggagaggctgccgt
ccgggtgggggcagggagacagatcagagaaggaaacgtgacccgagcccagtctccccattttattcctggatcaggatgagaacccaaacatcccaaagcctgtgagcttcagggtgaaggagaccgt
gtgccccaggccgagccagcagcccgcggagcagtgtgacttcaaggagaatggggtgagcctgggggcagggattgagggctggatcaatgcttctcagcacaagctgaacagggaacttcagggacaat
ttccagcccctgggggtgaggtgggctgagcatgggaggttatggcccagggtttccagtttgacctggaaactccccttccagctggtgaaacagtgtttggggacagtcagcctggacccgtccgatg
accagtttgacataaactgtaatgaggtgagtggccccttctgtgttggcatatgctaaaaaggtgagttgagaaacatcctttggaccaatgacccgctgctccgtctagagcagagaaaaggccctcct
acccgggcccaccctttccagtccccaggtctccagccctggctctgtatcctttagagaagtggctgtctaatggggtccccacccaggaactgacagtaaggcagattctcggccccactgagacctcc
tgaatcagactttggggtgggacccaggcatttgtattttcacaaggcctccaggggattctgacagtgctgaagttgtgacaccctgactcgagtcatggtcttgaggccatgctccagtccatctttgc
ccagatgggcttgtgacccttggaagccccttgtcatctctgggatcagtttccccatatgttgtgggtttaggaattcaaccacatgccccaaagatcaccgccagaggatgatctggggccaaagttcc
tttggtggctcagtttgggagttgttcaggtggggagagagtggtcttctcttgacccttgcccaatcccacaataatctcttccattgtggttcgcagcttcagagtgtcagggctaaacgacctcggcc
tcccaaatctcggcgtcctcacaggccaaggcgaaggccacggccatggttcccgccaaggttccccggaaaacggtga

Saiga tatarica

CNEQSVRSRRRPPKPRRPHRPRPRPRPWFPPRFPGKR

GenBank: PVIK010110818.1

contig_start_tgtaatgaggtgagtagccccttctgtgttgggcagatgctaaaagggtgggttgagaagcatcctttggaccaatgacccgctgctctgtctagggcagagagaaggccctcctac
ctgggcccaccttccccagtccccaggtctccagccctggctctgcatcccttacagaagtgtctgtctaatggggtccccacccaggaactgacagtaaggcagattcccagccccgctgagacctcctg
aatcagactttgggtgggacccaggcatttgtattttcacaagaactccaggggactctgacagtgctgaagttgtgacaccctgactcgagttgtggtcttgaggccatgctccagtccatctttgccc
acatgggcttgtgacccttggaagccccttgtcatctctgggatcagtttccccatatgttgtgggtttaggaattcaaccacacgccccaaagatcactgccagaggatgacctgggccatagttcctt
tggtggctcagtttgggggttgttcaggtggggagagagtggtcttctcttgacccttgcccagtcccacaagtaatctcttccattgtggttcacagtttcagagtgtcaggtctagaagacggcctccc
aaacctcggcgtcctcacaggccgcgcccaaggccacggccatggttcccaccaaggttccccggaaaacggtga

Connochaetes taurinus

METQRASLSLGRCSLWLLLLGLALPSASAQALSYREAVLRAVDRINDGSTEANLYRLLELDPPPKDVEDRGARKPVSFRVKETVCPRPSQQPLEQCDFKEDGLVKQCLGTVSLDPSDDQFDINCNELQSVR
ARRPRPPKPRPPHRPRPRPLPWFPPRFPGKR

GenBank: SJXO01002629.1

atggagacccagagggccagcctctccctggggcgctgttcgctgtggctactgctgctgggactagcgctgccctcggccagcgcccaggccctcagctacagggaggccgtgcttcgcgctgtggatcg
catcaatgatgggtccacagaagctaatctctaccgcctcctggagcttgacccgcctcccaaggacatgagttggggagggtgctgaatcatcttcccccagccttggccacactgtcgccccttcggtc
aggctggtcctcctgtcaggaaggcacttttcctctaggtgggttaaccttcccagacctggggcccctcccagcaccaggcttcctgccttagcatctctgctgtggggtcaggcgccctgcacacctgg
ctccagctccctggacttctgggagctccagggatggagggatcacaggctctgtgaggtgacttccctgctaatgtcccccctgctcctcggtgtctccctgccaggagggcctctctcagcctggaggct
ccagtgacgagggctctccctgcaggcggccctgacctccctgggcccctctgaggggaggcgctgccagcagcgctgctgtgagggccgctcctgctctctgtgtgcccgtgaggccaggcacgggctcc
gtgccctcccctgtgctcccagcaccaagcccagggccggacacacagcgggctggagaggctgctgtctgggtgggggcagggagacagatcagagaaggaaacatgagccgagcccagtctccccatt
ttgatcgttgaccaggtggaggaccggggagctcgaaagcctgtgagcttcagggtgaaggagactgtgtgccccaggccgagccagcagcccctggagcagtgtgacttcaaggaggatggggtgagcct
gggggcagggagtgagggctggatcaatgcttctcagtgctagctgaacagggaacttcagggacgatttccagcccctgggggtgaggtgggctgagcctgggagattatggcccagggtttccaatt
tgacctggaaactccccttccagttggtgaaacagtgtttggggacagtcagcctggacccgtccgatgaccagtttgacataaactgtaatgaggtgagtggccccttctgtattgggcatatgctaaca
aggtgggttgagaaacatcctttggaccaatgacccactgctccgtgtagggcagagaaaggccctcctacccgggcccactctccccagtccccaggcctccagccctggctctgcatccctaagagaa
gtggctgtctaatggggtccccacccgggaactgacagtaaggcagattctcagccccactgagacctcctgaatcagactttggggtgggacccaggcatttgtattttctcaaggcctccaggggcttc
tgacagtgctgaagttgtgacaccctgactcgagtcatggtcttgaggccatgctccagtccatctttgcccagatgggctgtgacccttggaagccccttgtcatctctgggatcagtttccccatatgt
tgtgggtttaggaattcaaccacatgccccaaagatcaccgccagaggatgatctggagccaaagttcctttggtggctcagtttggggggttgttcaggtggggagagagtggtcttctcttgacccttgc
ccaatcccacaagtaatctcttccattgtggttcacagcttcagagtgtcagggctagacgacctcggcctcccaaacctcggcccccctcacaggccaaggccaaggccactgccatggttcccaccaagg
ttcccgggaaaacggtga

Intron retention in 1st exon

Intron-retaining mediated premature termination codon + nonsense-mediated mRNA decay (NMD, >55bp from exon 3-end)

Aepyceros melampus

METQRASLSLXW.SLWLLLLGLVLPSASAQALSYREAVLRALDRINDGSSEANLYRLLEPSPPSLGVEDRGARKPVSFRVKETVCPRTSQQPAEQCDFKENGLVKQCLGTVSLDRPDDHFDITCDELQSVR
AVRVRPPKPRPPHRQRPRPRPWFPPRFPGKR

GenBank: SJXQ01009637.1

atggagacccagagggccagcctctccctg_gatggtgatcgctgtggctcctgctgctgggactagtgctgccctcggccagcgcccaggccctcagctacagggaggctgtgcttcgcgctctggatcg
catcaatgatgggtcctcagaagctaatctctaccgcctcctggagccatctcccccagccttggccacactgtcgccccttcgctctggctggtcctcctgtcaggaaggcacttttccctctaagcgg
gctcccacctcttccaggagaccttcccagacctgagaccactcccagcaccaggcttcctgccttagcatctctgctctgggaacaggcgccctgcacacctggctcaggctccctagacttctgggagc
tcccgggatggagggggcacaggctctgtgaggtgactttctgctaatgtcccctgcacctcggtgtctctctgccaggagggcctctgtcagcctggaggctccagtgacaagggctctccctgcaggc
ggccctgacctccctcagcctctgaggggaggcgctgccagcagcgctgctgtgagggccgctcctgctctctgtgtgcccgtgaggccgggcacgggctctgtgccctccctgtgctcccagcagcaagc
ccagggccggacacacaggggggctggagaggctgctgtccaggtggggtcagggagacagatcagagaaggaaagatgagccgagcccagtctccccactttgattgttgaccaggtggaggaccggga
gctcgaaagcctgtgagcttcagggtgaaggagaccgtgtgccccaggacgagccagcagcccgcggagcagtgtgacttcaaggagaatggggtgagcatgggggcggggagtgagagctgggatcagtg
cttctcagcgcgagctgaacagggaacttcagggacgatctccagccccttggggtgaggtgggctgagcgtgggaggttatggcccagggcctccagtgtgacctggaaagtccccttacagctggtga
agcagtgtttggggacagtcagcctggaccggcccgatgaccactttgacataacctgtgatgaggtgagtggcccctctgtgatggtatatgctaacaaggagggttgagaaacatcctttggaccaatg
ccccgctgctccgtctagggcagggaaaggccctcctacccgggcccaccctccccagtccccaggtctccagccctggaaaagcctgtgagcttcagggtgaaggagaccgtgtgccccaggacgagcc
agcagcccgcggagcagtgtgacttcaaggagaatggggtgagcatgggggcggggagtgagagctgggatcagtgcttctcagcgcgagctgaacagggaacttcagggacgatctccagccccttgggg
gtgaggtgggctgagcgtgggaggttatggcccagggcatccagtgtgacctggaaagtccccttacagctggtgaagcagtgtttggggacagtcagcctggaccggcccgatgaccactttgacataac
ctgtgatgaggtgagtggcccctctgtgatggtatatgctaacaaggagggttgagaaacatcctttggaccaatgccccgctgctccgtctagggcagggaaaggccctcctacccgggcccaccctcc
ccagtccccaggtctccagccctggctctgtatcctttagagaagtggctgtcttctggggtccccacccaggaactgacagtaaggcagcttctcagccccactgagacctcctgaatcagactttgggg
tgggacccaggcatttgtattttcacaaggcctccaggggattctgacagtgctaaagttgtgacaccctgactcgagttatggtcttgaggccatgctccagtccatctttgcccagatgggcttgtgac
ccttggaagccccttgtcatctctgggatcagtttccccgtatgttgtaggtttagaaattcagccacatgccccaaagatcactgccagaggatgatttggggccaaatttcctttggtggctcagtttg
ggggttcaggtgggagagagagagagtggtcttctcttgaccctggtccaatcccacaagtaatctcttccattgtggttcacagcttcagagtgtcagggctgtacgagttcggcctcccaaacctcgg
cctcctcacaggcaaaggccaaggccacggccatggttcccgccaaggttccctggaaaacggtga

Frame shift mediated (in 1st exon) premature termination codon + NMD

Intron retention in 1st exon

Sylvicapra grimmia

METQRASLSLGHCSLWLLLLGLALPSASAQVLSYREAVLRAVDQFNEKSSEANLYRLLELDPPPEQDVEDRGARSL.ASG.RRPCAPGRAGSPPEQCDFKENGLVKQCLGTVSLDRSKDQFDINCNELQSV
RAIRPRPPKPRPPQRPRPRPRPWFPPRFPGKR

GenBank: SJYH01005526.1

atggagacccagagggccagcctctccctggggcactgttcgctgtggctcctgctgctgggactagcgctgccctcggccagtgcccaggtcctcagctacagggaggccgtgcttcgtgctgtggatca
gttcaatgagaagtcctcagaagctaatctctaccgcctcctggagctagacccgcctcccgagcaggatgtgagttggggaggggctgggaaggggatctgtctcctgacatcctcggccacactgttg
ccccttcgctcaggctggtcctcctgtcaggaaggcacttttccctctaggcgggctcccacctcttccaggagaccttcccagacctggggcccctcccggcaccaagcttcctgccttagcatctctgc
tgtgggatcaggtgccctgcacacctggctcaggctcccgggacttctgggagctccaggggtggaggggtcacaggctctgtgaggtgacttccctgctaatgtcccctgcacctcggtgtctccctgc
caggagggcctctgtccgcctggaggctccagtgaccagggctctccctgcaggcggccctgaccgccctcggcccctctgcggggaggcgctgccggcagcgctgctgtgagcgccgctcctggtctctg
tgtgcccgcgagtccgggcgcgggctctgtgccccgccccgccccgggctcccagcacccagcccagggccggacacacaggggctggagaggctgctgtccgggtgggggcagggagacaggtcagaga
aggaaacgtgagccgagcccagtctccccaccttgatcgttgaccaggtagaggaccggggagctcgaagcctgtgagcttcagggtgaaggagaccgtgtgccccaggccgagccggcagcccccggag
cagtgtgacttcaaggagaatggggtgagcctgggggcggggactgagggctgggatgaatgcttctcagcgcaagctgaacagggaacttcaggatgatttccagcccctgggggtgaggtgggctga
gcctgggaggttatggcccagggtttccagtttgacctggaaactccccttccagctggtgaaacagtgcttggggacagtcagcctggaccggtccaaagaccagtttgacataaactgtaatgagatga
gtggccccttctgtgttgggcatatgctaacaaggtgggttgagaaacatcctttggaccaatgacccgctgctctgtctagggcagagaaaaggccctcctacccaggcccaccctccccagtccccagg
tctccagccatggctctgcatcctttagagaagtggctaatggggtccccacccaggaactgacagtaaggcagattctcagccccactgagacctcctgaatcagactttggggtgggacccaggcattt
gtattttcataaggcctccaggggattctgacagtgccgaagttgtgacaccctgactcgagtcacggtcttgaggccatgctccagtccatctttgcccagatgggcttgtgacccttagaagcccttg
tcatctctgggatcagtttccccatatgttgtgggtttaggaattcaaccacatgccccaaagatcactgccagaggatgatctggggccaaagttcctttggtggcccagtttggggttgttcaggtgg
ggagagagtggtcttctcttgacccttgcccaatcccacaagtaacctcttccattgtggttcacagcttcagagtgtcagggctatacgacctcggcctcccaaacctcggcctcctcagaggccaaggc
caaggccacggccatggttcccaccaaggttccccgggaaacggtga

Premature termination codons in 2nd exon + NMD

Intron retention in 3rd exon

Bos taurus

MKT.RASLSLGRWSLWLLLLGLALPSASAQALSYREAVLRAVDRINDGSTEAHLYRLLELDPPPKDVEDWGARKAVSFRVKETVCPRPSLQPPEQCDFKENGLVKQCLGTVSLDRSDDQFDINCNELQSVR
AN.PQPPKPRPPHRPRPRPRPWFPPRFPGKR

GenBank: NKLS02000022.1

atgaagacttagagggccagcctctccctgggacggtggtcactgtggctactgctgctgggactagcgctgccctcggccagcgcccaggccctcagctacagggaggctgtgcttcgtgctgtggatcg
catcaatgatgggtccacagaagctcatctctaccgcctcctggagctagacccgcctcccaaggatctccctgggagccatctcccccccccagctttggccacactgtcgccccttcgctcaggctggt
cctcctgtcaggagggcactttccctctgggtgggttcccatctcttccaggaaaccttcccagacctggggccctcccagcaccaggcttcctgccttagcatctctgctgtgggaacaggcgccctgc
acacctggctcaggctccctggacttctgggagctccagggatggggggtcacaggctctgtgaagtgacttctctgctaatgtcctctctgcacctcggtgtctctctgccagggaggcctctgtcagc
ctggaggctccagtgacaagggctctccctgcaggtggccctgacctccctcagcccctgtgaggggaggcgctgccatcagcgctgctgtgagggccgctcctgctttctgtgtgcccgtgaggccgggc
acgggctctgtcccctcccctgtgctcccagcaccaagcccagagcctgacacacaggggctagagaggctgccgtccgggttgggggcagggagacagatcagagaaggaaacatgagcctgagcccag
tctccccactttgatcctcgaccaggtggaggactggggagctcgaaaggctgtgagcttcagggtgaaggagactgtgtgccccaggccgagcctgcagcccccagagcagtgtgacttcaaggagaatg
ggctgaccttgggggctgagactgagggctgggatcaatgcttctcagcgcgagctgaacagggaacttcagggaatgtttccagcccctggcaggtgaggtaagctgagcctgggagattatggcccggg
gtttccagtttgaccttgagctccccttccagctggtgaaacagtgtttggggacagtcagcctggaccggtccgatgaccagtttgacataaactgtaatgaggcgagtggccccttctgtgttgggcat
atgctaacagggtgggttgagaaacatcctttggaccaatgacctgctgctccatctagggtagagaaaaggccctcctatctgggcccaccctccccaatccctaggtctccagccctggctctgcatcc
tttagagaagtggctgtctaacggggtccccacccaggaactcacaggaaggcagattctcagccccaccgagactcctgaatcagactttggggtgggccccggcatttgcgctttcacaaggcctccag
ggggttctgacagtgctgaaattgtggcagcctgacctgggccgtggtctcgaggccatgctccagtctacctttgcccggatgggcttgtgacccttggaagccccttgtcatctctgggatcagtttcc
ccatatgttgtgggtttagggattcaatcacatgctccaaagatcactgccagaggatgatctggggccaaagttcctttggtggctcagtttggggttgttcatgtggggagagagtggtcttctcttg
acccttgcccagtctcacaagtaatctcttccattgtggttcacagcttcagagtgtcagggctaactgacctcagcctcccaaacctcggcctcctcacaggccaaggccaaggccacggccatggttcc
caccaagattccctggaaaacggtga

Premature termination codons in 1st and 4th exon + NMD

Intron retention in 1st, 2nd, and 3rd exon

Bos gaurus

MKT.RASLSLGRWSLWLLLLGLALPSASAQALSYREAVLRAVDRINDGSTEAHLYRLLELDPPPKDVEDWRARKAVSFRVKETVCPRPSLQPPEQCDFKENGLVKQCLGTVSLDRSDDQFDINCNELQSVR
AN.PQPPKPRPPHRPRPRPRPWFPPRFPGKW

GenBank:JACAOC010000022.1

atgaagacttagagggccagcctctccctggacggtggtcactgtggctactgctgctgggactagcgctgccctcggccagcgcccaggccctcagctacagggaggctgtgcttcgtgctgtggatcg
aatcaatgatgggtccacagaagctcatctctaccgcctcctggagctagacccgcctcccaaggatctccctgggatccatctccccccagctttggccacactgtcgccccttcgctcaggctggtcct
cctgtcaggagggcactttccctctgggtgggttcccatctcttccaggaaaccttcccagacctgggggccctcccagcaccaggcttcctgccttagcatctctgctgtgggaacaggcgccctgcaca
cctggctcaggctccctggacttctgggagctccagggatggggggtcacaggctctgtgaagtgacttctctgccaatgtcccctctgcaccttggtgtctctctgccagggaggcctctgtcagcctg
gaggctccagtgacaagggctctccctgcaggcggccctgacctccctcagcccctctgaggggaggcgctgccatcagcgctgctgtgagggccgctcctgctttctgtgtgcccgtgaggccgggcacg
ggctctgtcccctcccctgtgctcccagcaccaagcccagagcctgacacacaggggctagagaggctgccgtccgggttgggggcagggagacagatcagagaaggaaacatgagcccgagcccagtct
ccccactttgatcctcgaccaggtggaggactggcgagctcgaaaggctgtgagcttcagggtgaaggagaccgtgtgccccaggccgagcctgcagcccccggagcagtgtgacttcaaggagaatgggg
tgaccttgggggctgagactgagggctgggatcaatgcttctcagcgcgagctgaacagggaacttcaggaatgtttccagcccctggcaggtgaggtaagctgagcctgggagattatggcccggggtt
tccagtttgaccttgagctccccttccagctggtgaaacagtgtttggggacagtcagcctggaccggtccgatgaccagtttgacataaactgtaatgaggcgagtggccccttctgtgttgggcatatg
ctaacagggtgggttgagaaacatcctttggaccaatgacccgctgctccgtctagggtagagaaaaggccctcctatctgggcccaccctccccaatccctaggtctccagccctggctctgcatcctttt
agagaagtggctgtctaacggggtccccacccaggaactcacaggaaggcagattctcagccccaccgagactcctgaatcagactttggggtgggccccggcatttgcgctttcacaaggcctccagggg
gttctgacagtgctgaaattgtggcagcctgacctgggccgtggtctcgaggccatgctccagtctacctttgcccggatgggcttgtgacccttggaagccccttgtcatctctgggatcagtttcccca
tatgttgtgggtttagggattcaatcacatgctccaaagatcactgccagaggatgatctggggccaaagttcctttggtggctcagtttgggggttgttcatgtggggagagagtggtcttctcttgacc
cttgcccagtctcacaagtaatctcttccattgtggttcacagcttcagagtgtcagggctaactgacctcagcctcccaaacctcggcctcctcacaggccaaggccaaggccacggccatggttcccac
caagattccctggaaaatggtga

Premature termination codons in 1st and 4th exon + NMD

Intron retention in 1st and 3rd exon

Bubalus bubalis

MKT.RASLSLGRWSLWLLLLGLVLPSASAQALSYREAVLRAVDQLNDGSTEANLYCLLELDLPPKDVEDRGARKPVSFRVK.TVCPRPSLQPPEQCDFKENGLVKQCLGTVSLDRSDDQFDINCNELQSVR
AK.PQPPKPRPPHRPRPRPRPWFPPRFPGKR

GenBank: PZYV01000014.1

atgaagacttagagggccagcctctccctggggcgctggtcactgtggctactgctgctgggactggtgctgccctcggccagtgcccaggccctcagctacagggaggccgtgcttcgtgctgtggatca
gctcaatgatgggtccacagaagctaatctctactgcctcctggagctagacctgcctcccaaggatgtgagttggggaggggctgagccatctccccccagctttggccacactgtcgccgcttcactaa
ggctggttctcttgtcaggaaggcacttttccctctaggtgggttcccatctcttccaggaaaccttcccagacctggggccctcccagcaccagccttcctgtcttagcatctctgctgtgggaacaggt
gccctgcacacctggctcaggctccctggacttctgggagctccagggatggggggtcacaggctctgtgaggtgacttccctgctactatcccacctgaaccttggtgtctctctgccaggggagctc
cgtcagcctggaggctccagtgacaagggccctccctgcaggcggccctgacctccctcagcccctctgaggggaggcgctgccatcagcgctgctgtgagggccgcttctgctctctgtgtgcccgtgag
gccgggcacgggctctgtcccctcccctgtgctcccagcaccaagcccagggcctgacacacaggggctggagaggctgcggtctgggtgggggcagggagacagatcagagaaggaaacatgagcccg
agcccagtctccccactttgatccttgaccaggtggaggaccggggagctcgaaagcctgtgagcttcagggtgaagtagactgtgtgccccaggccgagcctgcagcccccggagcagtgtgacttcaag
gagaatggggtgagcctggggactgagactgagggctgggatcaatgcttctcagcgtgagctgaacagggaacttcagggaatgtttccagcccctggcaggtgaggtgggctgagcctgggagattatg
gcctggggtttccagtttgaccttgagctccccttccagctggtgaaacagtgtttggggacagtcagcctggaccggtccgatgaccagtttgacataaactgtaatgaggtgagtggccccttctgtgt
tgggcatatgctaacagggtgggttgagaaacatcctttggaccaatgacccgctgctccgtctagggcagagaaaaggccctcctatctgggcccaccctccccaatccccaggtctccagccctggctc
tacatcctttagagaagtggctgctaatggggtccccacccaggaactcacagtaaggcagattctcagccccactgagacctcctgaatcagactttggggtggggccccggcatttgcattttcacaag
gcctccaggggattctgacggtgctgaaattgtgacaccctgacttgagtcatgttcttgaggccatgctccagtccatctttgcctggatgggcttgtgatccttggaagccccttgtcatctctgggat
cagtttccccatatgttgtgggtttagggattcaaccacatgctccaaagatcactgccagaggatgatctggggccaaagttcctttggtggctcagtttggggggttgttcatgtggggagagagtggtc
ttctcttgacccttgctcagtcccacaagtaatttcttccattgtggttcacagcttcagagtgtcagggctaagtgacctcagcctcccaaacctcggcctcctcacaggccaaggccaaggccacggcc
atggttcccgccaaggttccccggaaaacggtga

Premature termination codons in 1st, 2nd, and 4th exon + NMD

Bubalus depressicornis

MKT.RASLSLGRWSLWLLLLGLVLPSASAQALSYREAVLRAVDQLNDGSTEANLYCLLELDLPPKDVEDRGARKSVSFRVKETVCPRPSLQPPEQCDFKEYGLVKQCLGTVSLDRSDDQFDINCNELQSVR
AK.PQPPKPRPPHRPRPRPRPWFPPRFPGKR

GenBank: JAMXBS010052031.1

atgaagacttagagggccagcctctccctggggcgctggtcactgtggctactgctgctgggactggtgctgccctcggccagcgcccaggccctcagctacagggaggccgtgcttcgtgctgtggatca
gctcaatgatgggtccacagaagctaatctctactgcctcctggagctagacctgcctcccaaggatgtgagttggggaggggctgagccatctcccccagctttggccacactgtcgccgcttcactaa
ggctggttctcttgtcaggaaggcacttttccctctaggtgggttcccatctcttccaggaaaccttcccagacctgggccctcccagcaccaggcttcctgtcttagcatctctgctgtgggaacaggt
gccctgcacacctggctcaggctccctggacttctgggagctccagggatgggagggtcacaggctctgtgaggtgacttccctgctactatcccacctgaaccttggtgtctctctgccaggggagctc
cgtcagcctggaggctccagtgacaagggccatccctgcaggcggccctgacctccctcagcccctgtgaggggaggcgctgccatcagcgctgctgtgagggccactcctgctctctgtgtgcccgtgag
gctgggcacgggctctgtcccctcccctgtgctcccagcaccaagcccagggccggacacacaggggggctggagaggctgcgggctgggtgggggcagggagacagatcagagaaggaaacatgagcccg
agcccagtctccccactttgatccttgaccaggtggaggaccggggagctcgaaagtctgtgagcttcagggtgaaggagactgtgtgccccaggccgagcctgcagcccccggagcagtgtgacttcaag
gagtatggggtgagcctggggactgagactgagggctgggatcaatgcttctcagcgtgagctgaacagggaacttcagggaatgtttccagcccctggcaggtgaggtgggctgagcctgggagattatg
gcccggggtttccagtttgaccttgagctccccttccagctggtgaaacagtgtttgggcacagtcagcctggaccggtccgatgaccagtttgacataaactgtaatgaggtgagtggccccttctgtgt
tgggcatatgctaacagggtgggttgagaaacatcctttggaccaatgacccgctgctccgtctagggcagagaaaaggccctcctatctaggcccaccctccccaatccccaggtctccagccctggctc
tacatcctttagagaagtggctgctaatggggtccccacccaggaactcacagtaaggcagattctcagccccactgagacctcctgaatcagactttggggtggggccccggcatttgcattttcacaag
gcctccaggggattctgacggtgctgaaattgtgacaccctgacttgagtcatggtcttgaggccatgctccagtccatctttgcccggatgggcttgtgacccttggaagctccttgtcatctctgggat
cagtttccccatatgttgtgggtttagggattcaaccacatgctccaaagatcactgccagaggatgatctggggccaaagttcctttggtggctcagtttgggggttgttcatgtgggagagagtgatc
ttctcttgacccttgcccagtcccacaagtaatctcttccattgtggttcacagcttcagagtgtcagggctaagtgacctcagcctcccaaacctcggcctcctcacaggccaaggccaaggccacggcc
atggttcccgccaaggttccccggaaaacggtga

Premature termination codons in 1st, and 4th exon + NMD

Bison bison

MKT.RASLSLGRWSLWLLLLGLALPSASAQALSYREAVLRAVDRINDGSTEAHLYRLLELDPPPKDVEDWGARKAVSFRVKETVCPRPSLQPPEQCDFKENGLVKQCLGTVSLDRSDDQFDINCNELQSVR
ANRPQPPKPRPPHRPRPRPRPWFPPRFPGKR

GenBank: JPYT01263870.1

atgaagacttagagggccagcctctccctggacggtggtcactgtggctactgctgctgggactagcgctgccctcggccagcgcccaggccctcagctacagggaggctgtgcttcgtgctgtggatcg
catcaatgatgggtccacagaagctcatctctaccgcctcctggagctagacccgcctcccaaggatctccctgggatcgatctccccccagctttggccacactgtcgcccctttgctcaggctggtcct
cctgtcaggagggcactttccctctgggtgggttcccatctcttccaggaaaccttcccagacctggggccctcccagcaccaggcttcctgccttagcatctctgctgtgggaacaggcgccctgcaca
cctggctcaggctccctggacttctgggagctccagggatgggggtcacaggctctgtgaagtgacttctctgccaatgtcccctctgcacctcggtgtctctctgccagggaggcctctgtcagcctg
gaggctccagtgacaaaggctcttcctgcaggtggccctgacctccctcagcccctgtgaggggaggtgctgccatcagcgctgctgtgagggccgctcctgctttctgtgtgcccgtgaggccgggcacg
ggctctgtcccctcccctgtgctcccagcaccaagcccagagcctgacacacaggggctagagaggctgcagtccgggttgggggcagggagacagatcagagaaggaaacatgagcctgagcccagtct
ccccactttgatcctcgaccaggtggaggactggggagctcgaaaggctgtgagcttcagggtgaaggagactgtgtgccccaggccgagcctgcagcccccagagcagtgtgacttcaaggagaatgggg
tgaccttggggctgagactgagggctgggatcaatgcttctcagcgcgagctgaacagggaacttcagggaatgtttccagcccctggcaggtgaggtaggctgagcctgggagattatggcccggggtt
tccagtttgaccttgagctccccttccagctggtgaaacagtgtttggggacagtcagcctggaccggtccgatgaccagtttgacataaactgtaatgaggcgagtggccccttctgtgttgggcatatg
ctaacagggtgggttgagaaacatcctttggaccaatgacccgctgctccgtctagggtagagaaaaggccctcctatctgggcccaccctccccaatccctaggtctccagccctggctctgcatccttt
agagaagtggctgtctaacggggtccccacccaggaactcacagtaaggcagattctcagccccaccgagactcctgaatcagactttgggtgggccccggcatgtgcactttcacaaggcctccagggg
gttctgacagtgctgaaattgtggcagcctgacctgggccatggtctcgaggccatgctccagtccacctttgcccggatgggcttgtgacccttggaagccccttgtcatctctgggatcagtttcccca
tatgttgtgggtttagggattcaatcacatgctccaaagatcactgccagaggatgatctggggccaaagttcctttggtggctcagtttggggggttgttcatgtggggagagagtggtcttctcttgacc
cttgcccagtctcacaagtaatctcttccattgtggttcacagcttcagagtgtcagggctaaccgacctcagcctcccaaacctcggcctcctcacaggccaaggccaaggccacggccatggttcccgc
caagattccccggaaaacggtga

Premature termination codon in 1st exon + NMD

Intron retention in 1st and 3rd exon

Bos mutus

METQRASLSLGRWSLWLLLLGLVLPSASAQAPSYREAVLRAVDHIN.RSSEANLYRLLELDPPPKDVEDWGARKPVSFRVKETVCLRPSLQPPEQCDFKENGLVKQCLGTVSLDWSDDQFDINCNELQSVR
ANRPQPPKPRPPHRPRPRPRPWFPPRFPGKR

GenBank: VBQZ03000140.1

atggagacccagagggccagcctctccctggggcggtggtcactgtggctactgctgctgggactagtgctgccctcggccagcgcccaggcccccagctacagggaggccgtgcttcgtgctgtggatca
catcaattagcggtcctcagaagctaatctctaccgcctcctggagctagacccgcctcccaaggatctctctgggatccatctcccagggagatggagccatctccccccagctttggccacactgtcgc
cccttcgctcaggctggtcctcctgtcaggagagcactttccctctgggtgggttcccacctcttccaggaaaccctcccagacctgggccctcccagcaccaggcttcctgccttagcatctctgctg
tgggaacaggtgccctgcacacctggctcaggctccctggacttctgggagctccagggatggggggtcacaggctctgtgaagtgacttctctgctaatgtcccctctgcacctcggtgtctctctgcc
agggaggcctctgtcagcctggaggctccagtgacaagggctctccctgcaggcggccctgacctccctcagcccctgtgaggggaggcgctgccatcagcgctgctgtgagggccgctcctgctttctgt
gtgcccgtgaggctggacacgggctctgtcccctcccctgtgctcccagcaccaagcccagagcctgacacacaggggctagagaggctgccgtccgggttggggcagggagacagatcagagaaggaa
acatgagcctgagcccagtctccccactttgatcctcgaccaggtggaggactggggagctcgaaagcctgtgagcttcagggtgaaggaaactgtgtgcctcaggccgagcctgcagcccccggagcagt
gtgacttcaaggagaatggggtgaccttggggggttgagactgagggctgggatcaatgcttctcagcgcgaactgaacagggaacttcagggaatgtttccagcccctggcaggtgaggtgggctgagcct
gggagattatggcccggggtttccagtttgaccttgagctccccttccagctggtgaaacagtgtttggggacagtcagcctggactggtccgatgaccagtttgacataaactgtaatgaggcgagtggc
cccttctgtgttgggcatatgctaacagggtgggttgagaaacatcctttggaccaatgacccgctgctccgtctagggtagagaaaaggccctcctatctgggcccaccctccccaatccctaggtctcc
agccctggctctgcatcctttagagaagtggctgtctaacggggtccccacccaggaactcacagtaaggcagattctcagccccaccgagactcctgaatcagactttggggtgggccccggcatttgca
ctttcacaaggcctccaggggttctgacagtgctgaaattgtggcagcctgacctgggccgtggtctcgaggccatgctccactctatctttgcccggatgggcttgtgacccttggaagccccttgtcat
ctctgggatcagtttccccatatgttgtgggtttagggattcaatcacatgctccaaagatcactgccagaggatgatctggggccaaagttcctttggtggctcagtttggggggttgttcatgtggggag
agagtggtcttctcttgacccttgcccagtctcacaggtaatctcttccattgtggttcacagcttcagagtgtcagggctaaccgacctcagcctcccaaacctcggcctcctcacagaccaaggccaag
gccacggccatggttcccaccaagattccccggaaaacggtga

Premature termination codon in 1st exon + NMD

Intron retention in 1st and 3rd exon

Bos indicus

MKT.RASLSLGRWSLWLLLLGLALPSASAQALSYREAVLRAVDRINDGSTEAHLYRLLELDPPPKDVEDWGARKAVSFRVKETVCPRPSLQPPEQCDFKENGLVKQCLGTVSLDRSDDQFDINCNELQSVR
ANRPQPPKPRPPHRPRPRPRPWFPPRFPGKR

GenBank: PRDE01000015.1

atgaagacttagagggccagcctctccctgggacggtggtcactgtggctactgctgctgggactagcgctgccctcggccagcgcccaggccctcagctacagggaggctgtgcttcgtgctgtggatcg
catcaatgatgggtccacagaagctcatctctaccgcctcctggagctagacccgcctcccaaggatctccctgggagccatctccccccccccagctttggccacactgtcgccccttcgctcaggctgg
tcctcctgtcaggagggcacttttccctctgggtgggttcccatctcttccaggaaaccttcccagacctggggccctcccagcaccaggcttcctgccttagcatctctgctgtgggaacaggcgccctg
cacacctggctcaggctccctggacttctgggagctccagggatggggggtcacaggctctgtgaagtgacttctctgccaatgtcccctctgcacctcagtgtctctctgccagggaggcctctgtcag
cctggagggtccagtgacaagggctctccctgcaggcggccctgacctccctcagcccctgtgaggggaggcgctgccatcagcgctgctgtgagggccgctcctgctttctgtgtgcccgtgaggccggg
cacgggctctgtcccctcccctgtgctcccagcaccaagcccagagcctgacacacaggggctagagaggctgccgtccgggttgggggcagggagacagatcagagaaggaaacatgagcctgagccca
gtctccccactttgatcctcgaccaggtggaggactggggagctcgaaaggctgtgagcttcagggtgaaggagactgtgtgccccaggccgagcctgcagcccccagagcagtgtgacttcaaggagaat
gggctgaccttgggggctgagactgagggctgggatcaatgcttctcagcgcgagctgaacagggaacttcagggaatgtttccagcccctggcaggtgaggtaagctgagcctgggagattatggcccgg
ggtttccagtttgaccttgagctccccttccagctggtgaaacagtgtttggggacagtcagcctggaccggtccgatgaccagtttgacataaactgtaatgaggcgagtggccccttctgtgttgggca
tatgctaacagggtgggttgagaaacatcctttggaccaatgacctgctgctccgtctagggtagagaaaaggccctcctatctgggcccaccctccccaatccctaggtctccagccctggctctgcatc
ctttagagaagtggctgtctaacggggtccccacccaggaactcacaggaaggcagattctcagccccaccgagactcctgaatcagactttggggtgggcccccggcatttgcgctttcacaaggcctcca
ggggttctgacagtgctgaaattgtggcagcctgacctgggccgtggtctcgaggccatgctccagtctacctttgcccggatgggcttgtgacccttggaagccccttgtcatctctgggatcagtttc
cccatatgttgtgggtttagggattcaatcacatgctccaaagatcactgccagaggatgatctggggccaaagttcctttggtggctcagtttggggttgttcatgtggggagagagtggtcttctctt
gacccttgcccagtctcacaagtaatctcttccattgtggttcacagcttcagagtgtcagggctaaccgacctcagcctcccaaacctcggcctcctcacaggccaaggccaaggccacggccatggttc
ccgccaagattccccggaaaacggtga

Premature termination codon in 1st exon + NMD

Intron retention in 1st, 2nd, and 3rd exon

Bos frontalis

MKT.RASLSLGRWSLWLLLLGLALPSASAQALSYREAVLRAVDRINDGSTEAHLYRLLELDPPPKDVEDWGARKAVSFRVKETVCPRPSLQPPEQCDFKENGLVKQCLGTVSLDRSDDQFDINCNELQRVR
ANRPQPPKPRPPHRPRPRPRPWFPPRFPGKR

GenBank: RBVW01001332.1

atgaagacttagagggccagcctctccctggacggtggtcactgtggctactgctgctgggactagcgctgccctcggccagcgcccaggccctcagctacagggaggctgtgcttcgtgctgtggatcg
catcaatgatgggtccacagaagctcatctctaccgcctcctggagctagacccgcctcccaaggatctccctgggatccatctcccccccccagctttggccacactgtcgcccttcgctcaggctgg
tcctcctgtcaggagggcactttccctctgggtgggttcccatctcttccaggaaaccttcccagacctggggccctcccagcaccaggcttcctgccttagcatctctgctgtgggaacaggcgccctg
cacacctggctcaggctccctggacttctgggagctccagggatgggggggtcacaggctctgtgaagtgacttctctgccaatgtcccctctgcacctcggtgtctctctgccagggaggcctctgtcag
cctggaggctccagtgacaagggctcttcctgcaggtggccctgatctccctcagcccctgtgaggggaggcgctgccatcagcgctgctgtgagggccgctcctgctttctgtgtgcccgtgaggccggg
cacgggctctgtcccctcccctgtgctcccagcaccaagcccagagcctgacacacaggggctagagaggctgccgtccgggttggggcagggagacagatcagagaaggaaacatgagcctgagccca
gtctccccagtttgatccttgaccaggtggaggactggggagctcgaaaggctgtgagcttcagggtgaaggagactgtgtgccccaggccgagcctgcagcccccagagcagtgtgacttcaaggagaat
gggctgaccttgggggctgagactgagggctgggatcaatgcttctcagcgcgagctgaacagggaacttcagggaatgtttccagcccctggcaggtgaggtaagctgagcctgggagattatggcccgg
ggtttccagtttgaccttgagctccccttccagctggtgaaacagtgtttggggacagtcagcctggaccggtccgatgaccagtttgacataaactgtaatgaggcgagtggccccttctgtgttgggca
tatgctaacagggtgggttgagaaacatcctttggaccaatgacctgctgctccatctagggtagagaaaaggccctcctatctgggcccaccctccccaatccctaggtctccagccctggctctgcatc
ctttagagaagtggctgtctaacggggtccccacccaggaactcacaggaaggcagattctcagccccaccgagactcctgaatcagactttggggtgggccccggcatttgcgctttcacaaggcctcca
ggggttctgacagtgctgaaattgtggcagcctgacctgggccatggtctcaaggccatgctccagtcagggggttctgacagtgctgaaattgtggcagcctgacctgggccatggtctcgaggccatgc
tccagtctacctttgcccggatgggcttgtgacccttggaagccccttgtcatctctgggatcagtttccccatatgttgtgggtttagggattcaatcacatgctccaaagatcactgccagaggatgat
ctggggccaaagttcctttggtggctcagtttggggttgttcatgtggggagagagtggtcttctcttgacccttgcccagtctcacaagtaatctcttccattgtggttcacagcttcagagggtcagg
gctaaccgacctcagcctcccaaacctcggcctcctcacaggccaaggccaaggccacggccatggttcccgccaagattccccggaaaacggtga

Premature termination codon in 1st exon + NMD

Intron retention in 1st, 2nd, and 3rd exon

Bos grunniens

METQRASLSLGRWSLWLLLLGLVLPSASAQAPSYREAVLRAVDHIN.RSSEANLYRLLELDPPPKDVEDWGARKPVSFRVKETVCLRPSLQPPEQCDFKENGLVKQCLGTVSLDWSDDQFDINCNELQSVR
ANRPQPPKPRPPHRPRPRPRPWFPPRFPGKR

GenBank: VBZB01000023.1

atggagacccagagggccagcctctccctggggcggtggtcactgtggctactgctgctgggactagtgctgccctcggccagcgcccaggcccccagctacagggaggccgtgcttcgtgctgtggatca
catcaattagcggtcctcagaagctaatctctaccgcctcctggagctagacccgcctcccaaggatctctctgggatccatctcccagggagatggagccatctccccccagctttggccacactgtcgc
cccttcgctcaggctggtcctcctgtcaggagagcacttttccctctgggtgggttcccacctcttccaggaaaccctcccagacctggggccctcccagcaccaggcttcctgccttagcatctctgctg
tgggaacaggtgccctgcacacctggctcaggctccctggacttctgggagctccagggatgggggggtcacaggctctgtgaagtgacttctctgctaatgtcccctctgcacctcggtgtctctctgcc
agggaggcctctgtcagcctggaggctccagtgacaagggctctccctgcaggcggccctgacctccctcagcccctgtgaggggaggcgctgccatcagcgctgctgtgaggccgctcctgctttctgt
gtgcccgtgaggctggacacgggctctgtcccctcccctgtgctcccagcaccaagcccagagcctgacacacagggggctagagaggctgccgtccgggttgggggcagggagacagatcagagaaggaa
acatgagcctgagcccagtctccccactttgatcctcgaccaggtggaggactggggagctcgaaagcctgtgagcttcagggtgaaggaaactgtgtgcctcaggccgagcctgcagcccccggagcagt
gtgacttcaaggagaatggggtgaccttgggggttgagactgagggctgggatcaatgcttctcagcgcgaactgaacagggaacttcagggaatgtttccagcccctggcaggtgaggtgggctgagcct
gggagattatggcccggggtttccagtttgaccttgagctccccttccagctggtgaaacagtgtttggggacagtcagcctggactggtccgatgaccagtttgacataaactgtaatgaggcgagtggc
cccttctgtgttgggcatatgctaacagggtgggttgagaaacatcctttggaccaatgacccgctgctccgtctagggtagagaaaaggccctcctatctgggcccaccctccccaatccctaggtctcc
agccctggctctgcatcctttagagaagtggctgtctaacggggtccccacccaggaactcacagtaaggcagattctcagccccaccgagactcctgaatcagactttggggtgggcccggcatttgca
ctttcacaaggcctccaggggttctgacagtgctgaaattgtggcagcctgacctgggccgtggtctcgaggccatgctccactctatctttgcccggatgggcttgtgacccttggaagccccttgtcat
ctctgggatcagtttccccatatgttgtgggtttagggattcaatcacatgctccaaagatcactgccagaggatgatctggggccaaagttcctttggtggctcagtttggggttgttcatgtggggag
agagtggtcttctcttgacccttgcccagtctcacaggtaatctcttccattgtggttcacagcttcagagtgtcagggctaaccgacctcagcctcccaaacctcggcctcctcacagaccaaggccaag
gccacggccatggttcccaccaagattccccggaaaacggtga

Premature termination codon in 1st exon + NMD

Intron retention in 1st and 3rd exon

Tragelaphus imberbis

VEDKGARKPVSFRVKETVCPRPSLQTPEQYDFKENGLVKQCLGTVSLDRSDDQFDINCNELQSVRAKRPRPPKPRPPHRPRPRPRPWFPPRFPGKP

GenBank: SJYM01257328.1

contig_start_agggctctccctgcaggcggccctgacctccctcagcccctctgaggggaggcgctgccatcagcgctgctgtgagggctgctcctgctctctgtgtgcatgtgaggccgggcaggag
ctctgtcccctcccctgtgctcctggcaccaagcccagggccggacacacaggggctggagaggctgccgtccgggtgggggcagggagacagatcagagaaggaaacatgagcccgagcccagtctcc
ccactttgatcctccaccaggtggaggacaagggagctcgaaagcctgtgagcttcagggtgaaggaaactgtgtgccccaggccaagcctgcagaccccagagcagtatgacttcaaggagaatggggtg
agcctgggcgctgagactgagggctgggatcaatgcttctcagtgtgagctgaacagggaactcagggaatgtttccagcccctggcaggtgaggtgggctgagcctgggagattatggcccagggtttcc
agtttgaccctgagctccccatccagctggtgaaacagtgtttggggacagtcagcctggaccggtccgatgaccagtttgacataaactgtaatgaggtgagtggccccttctgtgttgggcatatgcta
acagggtgggttgagaaacgtcctttggaccaatgacccgctgctccgtcgagggcagagaaaggccctcctacctgggcccaccctccccaatccccaggtctccagctctagctctgcatcctttaga
gacgtggctgtctaatggggtccccacactggaactcacagtaaggcagattctcagccccactgagacctcctgaatcagaatttggggtggggcccaggcattgtattttcacaaggcctccagggat
tctgacagtgctgaaattgtgacaccctgactcgagtcatggtcttgaggccatgctccagtccatcttttcccagatgggcttgtgacccttggaagccccttgtcatctgtgggatcagtttccccata
tgttgtgggtttagggattcaaccacatgctccaaagatcaccgccagaggatgatctggggccaaagttcctttggtggctcagtttggggttgttcaggtggggagagagtggttttctcttgaccct
tgcgcagtcccacaagtaatctcttccattgtggttcacagcttcagagtgtcagggctaaacgacctcggcctcccaaacctcggcctcctcacaggccaaggccaaggccacggccatggttcccgcca
aggttccccggaaaaccgtga

conservative Cys mutation in 2nd exon

Tragelaphus strepsiceros

MKT.RASLSLGRCSLWLLLLGLVLPSASAQALSYREAVLRAVDRINDGSTEANLYCLLELDPRPKDVEDRGARKPVSFRVKETVCPRPSLQPPEQYDFKENGLVKQCLGTVSLDRSDDQFDINCNELQSVR
AKRPRPPKPRPPHRPRPRPRPWFLPGFPRKR

GenBank: SJYU01005288.1

atgaagacttagagggccagcctctccctgggacggtgttcactgtggctactgctgctaggactagtgctgccctcggccagcgcccaggccctcagctacagggaggccgtgcttcgtgctgtggatcg
catcaatgatgggtccacagaagctaatctctactgcctcctggagctagacccacgtcccaaggatgtgaattggggaggagctgagccatctccccccagccttggccacactgtcgcccctttgctca
ggctggttctcttgtcaggaaggcacttttccctctaggtgggctcccacctcttccaggaaaccttcccagacctggggccctcccagcaccaggcttcctgtcttagcatctctgctgtgggaacaggc
gccctgcacacctggctcagtctccctagacttctgggagctccagggatgggggtcacaggctctgtgacgtgacttccctgctaatgttcccccctgcacctcggtgtctccctaccagggggagctcc
gtcagcctggaggctctggtgacaagggctctccctgcaggcggccttgacctccctcagcccctctgagggggaggcgctgccatcagcgctgctgtgaggctgctcctgctctctgtgtgcacgtgagg
ccgggcaggagctctgtcccctcccctgtgctcccggcaccaagctcagggccggacacacaggggggctggagaggctgccgtctgggttcggggcagggagacagatcagagaaggaaacatgagcccaa
gccagtctccccatttgatcctccaccaggtggaggaccggggagctcgaaagcctgtgagcttcagggtgaaggaaactgtgtgccccaggccgagcctgcagcccccagagcagtatgacttcaagga
gaatggggtgagcctaggggctgagactgagggctgggatcaatgcttctcagtgtgagctgaacagggaactcagggaatgtttccagccgctggcaggtgaggtgggctgagcctgggagattatggcc
cagggtttccagtttgaccctgagctccccatccagctggtgaaacagtgtttggggacagtcagcctggaccggtccgatgaccagtttgacataaactgtaatgaggtgagtggccccttctgtgttgg
gcatatgctaacagggtgggttgagaaacgtcctttggaccaatgacccgctgctccgtcgagggcagagaaaggccctcctacctgggcccaccctccccaatccccaggtctccagctctagctctgc
atcctttagagacgtggctgtctaatgaggtccccacactggaactcacagtaaggcagattctcagccccactgagacctcctgaatcagaatttggggtggggcccaggcattgtattttcacaaggcc
tcgagaggattctgacagtgctgaaattgtgacaccctgactcgagtcatggtcttgaggccatgctccagtccatcttttcccagatgggcttgtgacccttggaagccccttgtcatctgtgggatcag
tttccccatatgttgtgggtttagggattcaaccacatgctccaaagatcaccgccagaggatgatctggggccaaagttcctttggtggctcagtttgggggttgttcaggtggggagagagtggttttc
tcttgacccttgcgcagtcccacaagtaatctcttccattgtggttcacagcttcagagtgtcagggctaaacgacctcggcctcccaaacctcggcctcctcacaggccaaggccaaggccacggccatg
gttcctgccagggttccccagaaaacgatga

Premature termination codon in 1st exon + NMD

conservative Cys mutation in 2nd exon

Tragelaphus buxtoni

VEDRGARKPVSFRVKETVCPRPSLQPPEQYDFKENGLVKQCLGTVSLDRSADQFDINCNELQSVRAKRPWPPKSQPPHRPRPRPRPWFPPRFPGKP

GenBank: SJYJ012900726.1

contig_start_cccctgtgctcccggcaccaagcccagggccggacacacaggggctggagaggctgccgtccgggtgggggcagggaaacagatcagagaaggaaacatgagcccgagcccagtct
ccccactttgatcctcgaccaggtggaggacaggggagctcgaaagcctgtgagcttcagggtgaaggaaactgtgtgccccaggccgagcctgcagcccccggagcagtatgacttcaaggagaatgggg
tgagcctagggggctgagactgagggctgggatcaatgcttctcagtgtgggctgaacagggaactcagggaatgtttccagcccttggcaggtgaggtgggctgagcctgggagattatggcccagggttt
ccagtttgaccctgagctccccatccagctggtgaaacagtgtttggggacagtcagcctggaccggtccgctgaccagtttgacataaactgtaatgaggtgagtggccccttctgtgttgggcatatgc
taacagggtgggttgagaaacatcctttggaccaatgacccgctgctccgtcgagggcagagaaaggccctcctacctgggcccaccctccccaatccccaggtctccagctctagctctgcatccttta
gagacgtggctgtctaatgaggtccccacactggaactcacagtaaggcagattctcagccccactgagacctcctgaatcagaatttggggtggggcccaggcattgtattttcacaaggcctccagggg
attctgactgtgctgaaattgtgacaccctgactcgagtcatggtcttgaggccatgctccagtccatcttttcccagatgggcttgtgacccttggaagccccttgtcatctgtgggatcagtttcccca
tatgttgtgggtttagggattcaaccacatgctccaaagatcaccgccagaggatgatctggggccaaagttcctttggtggctcagtttggggggttgttcaggtggggagagagtggttttctcttgacc
cttgcgcagtcccacaagtaatctcttccattgtggttcacagcttcagagtgtcagggctaaacgaccttggcctcccaaatctcagcctcctcacaggccaaggccaaggccacggccatggttcccgc
caaggttccccggaaaaccgtga

conservative Cys mutation in 2nd exon

Tragelaphus eurycerus

MKT.RASLSLGRCSLWLLLLGLVLPSASAQALSYREAVLRAVDPINDGSTEANLYCLLELDPPPKDVDRGARKPVSFRVKETVCPRPSLQPPEQYDFKENGLVKQCLGTVSLDRYDDQFDINCNELQSVRA
KRPWPPKPRPPHRPRPRPRPWFPPRFPGKP

GenBank: SJYI010177910.1

atgaagacttagagggctagcctctccctgggacggtgttcactgtggctactgctgctgggactagtgctgccctcggccagtgcccaggccctcagctacagggaggccgtgcttcgtgctgtggatcc
catcaatgatgggtccacagaagctaatctctactgcctcctggagctagacccacctcccaaggatgtgaattggggaggagctgagccatctct**_gap_**gggggctggagaggctgccgtccgggttgg
gggcagggagacagatcagagaaggaaacatgagcccgagcccagtctccccactttgatcctcgaccaggtggacaggggagctcgaaagcctgtgagcttcagggtgaaggaaactgtgtgccccaggc
cgagcctgcagcccccggagcagtatgacttcaaggagaatggggtgagcctaggggctgagactgagggctgggatcaatgcttctcagtgtgagctgagcagggaactcagggaatgtttccagcccct
ggcaggtgaggtgggctgagcctgggagattatggcccggggtttccagtttgaccctgagctccccatccagctggtgaaacagtgtttggggacagtcagcctggaccggtacgatgaccagtttgaca
taaactgtaatgaggtgagtggccccttctgtgttgggcatatgctaacagggtgggttgagaaacatcttttggaccaatgacccgctgctccgtcgagggcagagaaaaggccctcctacctgggctca
ccctccccaatccccaggtctccagctctggctctgcatcctttagagacgtggctgtctaatggggtccacacactggaactcacagtaaggcagattctcagccccactgagacctcctgaatcagaat
ttggggtggggcccaggcattgtattttcacaaggcctccaggggattctgacagtgctgaaattgtgacaccctgactcgagtcatggtcttgaggccatgctccagtccatcttttcccagatgggctt
gtgacccttggaagccccttgtcatctgtgggatcagtttccccatatgttgtgggtttagggattcaaccacatgctccaaagatcaccgccagaggatgatctggggccaaagttcctttggtggctca
gtttggggggttgttcaggtggggagagagtggttttctcttgacccttgcgcagtcccacaagtaatctcttccattgtggttcacagcttcagagtgtcagggctaaacgaccttggcctcccaaacctc
ggcctcctcacaggccaaggccaaggccacggccatggttcccgccaaggttccccggaaaaccgtga

Premature termination codon in 1st exon + NMD

conservative Cys mutation in 2nd exon

Tragelaphus spekii

MKT.RASLSLGRCSLWLLLLGLVLPSASAQALSYREAVLRAVDRINDGSTEANLYCLLELDPPPKDVDRGARKPVSFRVKETVCPRPSLQPPEQYDFKENGLVKQCLGTVSLDRSDDQFDINCNELQSVRA
KRPWPPKPRPPHRPRPRPRPWFPPRFPGKP

GenBank: SJYL010036573.1

atgaagacttagagggctagcctctccctgggacggtgttcactgtggctactgctgctgggactagtgctgccctcggccagtgcccaggccctcagctacagggaggccgtgcttcgtgctgtggatcg
catcaatgatgggtccacagaagctaatctctactgcctcctggagctagacccacctcccaaggatgtgaattggggaggagctgagccatctctccccagccttggccacactgttgcccctttgctca
ggctggttctcctgtcaggaaggcacttttccctctaggtgggctcccacctcttccaggagaccttcccagacctggggccctcccagcatcaggcttcctgccttagcatctctgctgtgggaacaggc
gccctgcacacctagctcaggctccctggacttctgggagctccaggatgggggggtcacaggctctgtgatgtgactttcctgctaatgtcccctctgcacctcggtgtctccctaccaggggagctt
ggtcagcctggaggctccggtgacaagggctctccctgcaggcggccctgacctccctcagcccctctgaggggaggcgctgccatcagcgctgctgtgagggctgctcctgctctctgtgtgcccgtgag
gccgggcacgggctctgtcccctcccctgtgctcccagcaccaagcccagggccggacacacaggggggctggagaggctgccgtccgggttgggggcagggagacagatcagagaaggaaacatgagcccg
agcccagtctccccactttgatcctcgaccaggtggacaggggagctcgaaagcctgtgagcttcagggtgaaggaaactgtgtgccccaggccgagcctgcagcccccggagcagtatgacttcaaggag
aatggggtgagcctaggggctgagactgagggctgggatcaatgcttctcagtgtgagctgaacagggaactcagggaatgtttccagcccctggcaggtgaggtgggctgagcctgggagattatggccc
ggggtttccagtttgaccctgagctccccatccagctggtgaaacagtgtttggggacagtcagcctggaccggtccgatgaccagtttgacataaactgtaatgaggtgagtggccccttctgtgttggg
catatgctaacagggtgggttgagaaacatcttttggaccaatgacctgctgctccgtcgagggcagagaaaggccctcctacctgggctcaccctccccaatccccaggtctccagctctggctctgca
tcctttagagacgtggctgtctaatggggtccacacactggaactcacagtaaggcagattctcagccccactgagacctcctgaatcagaatttgggggtgggcccaggcattgtattttcacaaggcct
ccaggggattctgacagtgctgaaattgtgacaccctgactcgagtcatggttttgaggccatgctccagtccatcttttcccagatgggcttgtgacccttggaagccccttggcatctgtgggatcagt
ttccccatatgttgtgggtttagggattcaaccacatgctccaaagatcaccgccagaggatgatctggggccaaagttcctttggtggctcagtttggggggttgttcaggtggggagagagtggttttct
cttgacccttgcgcagtcccacaagtaatctcttccattgtggttcacagcttcagagtgtcagggctaaacgaccttggcctcccaaacctcggcctcctcacaggccaaggccaaggccacggccatgg
ttcccgccaaggttccccggaaaaccgtga

Premature termination codon in 1st exon + NMD

conservative Cys mutation in 2nd exon

Tragelaphus oryx

MKT.RASLSLGRCSLWLLLLGLVLPLASAQALSYREAVLRAVDHINDGSTEANLYCLLELDPRPKDVEDRGA.KPVSFRVKETVCPRPSLQPPEQYDFKENGLVKQCLGAVGLDRSDDQFDINCNELQSVR
AKRPWPPKPRPPHRPRPRPRPWFPPRFPRKP

GenBank: SJYK013413680.1

atgaagacttagagggccagcctctccctgggacggtgttcactgtggctactgctgctgggactagtgctgcccttggccagcgcccaggccctcagctacagggaggccgtgcttcgtgctgtggatca
catcaatgatgggtccacagaagctaatctctactgccttctggagctagacccacgtcccaaggatgtgaattgggcaggagctgagccatctcccccagccttggccacactgtcgcccctttgctca
ggctggttctcttgtcaggaaggcacttttccctctaggtgggctcccacctcttccaggaaaccttcccagacctcgggccctcccggcaccaggcttcctgtcttagcatctctgctgtgggaacaggc
gccctgcacacctggctcagtctccctggacttctaggagctccagggatgggggg_contig_end

GenBank: SJYK013804480.1

contig_start_gcccctctgaggggaggcgctgccatcagcgctgctgtgaggctgctcctgctctctgtgtgcacgtgaggccgggcaggagctctgtcccctcccctgtgctcccggcaccaagcc
cagggccggacacacaggggctggagaggctgccgtctgggttgggggcagggagacagatcagagaaggaaacatgagcccaagcccagtcacccactttgatcctccaccaggtggaggacaggga
gcttgaaagcctgtgagcttcagggtgaaggaaactgtgtgccccaggccgagcctgcagcccccagagcagtatgacttcaaggagaatggggtgagcctaggggctgagactgagggctgggatcaatg
cttctcagtgtgagctgaacagggaactcagggaatgtttccagcccctggcaggttaggtgggctgagcctgggagattatggcccagggtttccagtttgaccctgagctccccatccagctggtgaaa
cagtgtttgggggcagtcggcctggaccggtccgatgaccagtttgacataaactgtaatgaggtgagtggccccttctgtgttgggcatatgctaacagggtgggttgagaaacgtcctttggaccaatg
acccgctgctccgtcgagggcagagaaaaggccctcctacctgggcccaccctccccaatccccaggtctccagctctagctctgcatcctttagagacgtggctgtctaatgaggtccccacactggaac
tcacagtaaggcagattctcagccccactgagacctcctgaatcagaatttggggtggggcccaggcattgtattttcacaaggcctccaggggattctgacagtgctgaaattgtgacaccctgactcga
gtcatggtcttgaggccatgctccagtccatcttttcccagatgggcttgtgacccttggaagccccttgtcatctgtgggatcagtttccccatatgttgtgggtttagggattcaaccacatgctccaa
agatcaccaccagaggatgatctggggccaaagttcctttggtggctcagtttgggggttgttcaggtggggagagagtggttttctcttgacccttgcgcagtcccacaagtaatctcttccattgtggt
tcacagcttcagagtgtcagggctaaacgaccttggcctcccaaacctcggcctcctcacaggccaaggccaaggccacggccatggttcccgccaaggttccccagaaaccgtga

Premature termination codons in 1st and 2nd exon + NMD

conservative Cys mutation in 2nd exon

Tragelaphus scriptus

MKT.RASLSLGRCSLWLLLLGLVLPSASAQALSYREAVLRAVDRIND/?/NCNELQSVRAKRPWPPKPWPPHRPRPRPRPWFPPRFPGKP

GenBank: SJXY01004217.1

atgaagacttagagggccagcctctccctggacggtgttcactgtggctactgctgctgggactagtgctgccctcggccagcgcccaggccctcagctacagggaggccgtgcttcgtgctgtggatcg
catcaatgat/gap/aaactgtaatgaggtgagtggccccttctgtgttgggcatatgctaacagggtgggttgagaaacgtcctttggaccaatgacccactgctctgtcgagggcagagaaaggccct
cctacctgcgcccaccctccccaatccccaggtctccagctctagctctgcatcctttagagacgtggctgtctaatgaggtccccacactggaactcacagtaaggcagattctcagccccactgagacc
tcctgaatcagaatttggggtggggcccaggcattgtattttcacaaggcctccaggggattctgacagtgctgaaattgtgacaccctgactcgagtcatggtcttgaggccatgctccagtccatcttt
tcccagatgggcttgtgacgcttggaagccccttgtcatctgtgggatcagtttccccatatgttgtgggtttagggattcaaccacatgctccaaagatcaccgccagaggatgatctggggccaaagtt
cctttggtggctcagtttggggggttgttcaggtggggagagagtgattttctcttgacccttgcgcagtcccacaagtaatctcttccattgtggttcatagcttcagagtgtcagggctaaacgaccttg
gcctcccaaaccttggcctcctcacaggccaaggccaaggccacggccatggttcccgccaaggttccccggaaaaccgtga

Premature termination codons in 1st exon + NMD

Capreolus capreolus

METQRASLSLGRWSLWLLLLGLVLPSVSAQVYSYWRAVLRAVDQFNVQSSEANLYRLLELDPPPEQDVEDQGA.KPVSVRVKETVCPKTSQQPPEQCDFRENGLVKQCVKTASLDWSDSQFDINCNELQSV
RAK.PRPPKPQPPHRPRPRPRPWFLPRFPGKW

GenBank: CATOUS010000013.1

atggagacccagagggccagcctctccctgggacggtggtcactgtggctactgctgctgggactagtgctgccctcggtcagcgcccaggtctacagctactggagagctgtgcttcgcgctgtggatca
gttcaatgtgcagtcctcagaagctaatctctaccgcctcctggagctagacccgcctccagagcaggacgtgagttggggaggggctgggaaggggatctgtctcctgacatccttggccacactgtcg
ccccttcactcaggctgttcctcctgtcaggaaagacagttctccctcttggtgggctcccacctcttccaggaaaccttcccagacctgggtcccctgccagccccaggcttcctgccttagcatctct
gctgtgggaacaggcgccctgcacacctggctcccaggacttccgggagctccagggatggaggggtcacaggctctgtgaggggacgtccctgctgaagccccctctgcaccgcggtgtctccttccca
gggaggcctctgtcagcctggaggttccaggtacaagggctctccctgcaggcggccctgacctccctcagcccctctgcgaggaggcgttgccctcagcgctgccgtgtggtccggtcctgctctctggg
cgcccgtgaggccggggacgggctctgtccactcccctgggctcccagcaccaagcccagggcttgggtggggcaaggagacagatcagagaaggaaacatgagcccgagcccagtctccccactttgat
ctttgaccaggtggaagaccagggagcttgaaagcctgtgagcgtcagggtgaaggagaccgtgtgccccaagacgagccagcagcccccggagcagtgtgacttcagggagaatggggtgagcttgggg
ctggaggctgagggctgggatagatgcttctcagcgcaagctgagcaggggccttcaggatgatttccagcccctgaggggtgaagttggctgagcctgggaggttatggccccggggggttccagtttga
cctggaaactccccttccagctggtgaaacagtgtgtgaagacagccagcctggactggtccgattcccaatttgacataaactgtaatgaggtgagtggccccttctgtgctgtgcggatgctaacaggg
taggttgtggaacatcctttggaccaatgacctgatgccccatccagggcagagaaaggccctcctacctgggcccaccctccccaatccccaggtctccagccctggctctgcatcctttagagaagtg
gctgtctaatggggtcgccacctaggaactgacagtaaggcagattctcagccccactgaagcctgctgaatcagactttggggtgggcccaggcatttgtatttttacaaggcctctggggggattctga
cagtgctgaagttgtgacaccctgactcgagtcatggtcttgaggccatgctccagtccatctttgcccggatgggcttgtgacacttggaagccccttgtcatctctgggatcagtttccccatatgttg
tgggtttaaggattcaaccacatgctccaaagatcaccgccagaggatgatctggggccaaagttcctttggtggcccagttcggggggttgttcaggtggggagagagtggtcttctcttgacccttgccc
agtcccacaagtgatctcttccattgtggttcacagcttcagagtgtcagggctaaatgacctcggcctcccaaacctcagcctcctcacagaccaaggccaaggccacggccatggtttctgccaaggtt
ccccggaaaatggtga

Premature termination codons in 2nd and 4th exons + NMD

Oryx gazelle

METQRASLSLERCSLWLLLLGLALPSASAQAPSYREAVLHAVDRINDGSTEANLYRLLELDPPPKDVEDREA.KPVSFKVKETVCPRLSQQPLEQCDFKENGLVKQCLGTVSLDPSDDQFDINCNELQSVR
ARRPRPPKPRPPHRPRPRPLPWFLPRFLGKR

GenBank: SJYN01009761.1, RAWW01009131.1

atggagacccagagggccagcctctccctggaacggtgttcgctgtggctactgctgctgggactagcgctgccctcagccagcgcccaggcccccagctacagggaggctgtgcttcacgctgtggatcg
catcaatgatgggtccacagaagctaatctctaccgcctcctggagcttgacccgcctcccaaggacgtgagctggggagggtgctgagccatcttcccccagccttggccacactgtcgccccttcgctc
aggctggtcctcctgtcaggaaggcactttctctctaggtaggttaaccttcccagacctgggtcatctcccggcaccaggcttcctgccttagcgtctctgctgtgggatcaggcgccccgcacacctgg
ttccggctccctggacttctgggagctccagggatggagggccacaggctctgtgaggtgacttccctgctaatgtccccccctgctccttggtatctccctgccaggaggcctctgtcagcctggaggct
ccagtgacgagggctcaccctgctggcggccctgacctccctgggcccctctgaggggaggcgctgccagcagcgctgctgtgagggccgcttctgctctctgtgtgcccatgaggccgggcacgggctct
gtgccctcccctgtgctcctagcaccaagcccagggccggacacacaggggggctggagaggctgctgtccgggtaggggacagggagacagatgagagaaggaaacatgagcccgagcccagtctccccac
tttgatcgttgaccaggtggaggaccgggaagctttgaaagcctgtgagcttcaaggtgaaggagactgtgtgccccaggctgagccagcagcccctggagcagtgtgacttcaaggagaatggggtgagcc
tgggggcagggagtgagggctgggatcaatgcttctcagtgctagctgaacagggaacttcagggacgatttccagcccctggcgggtgaggtgggctgagcctgggaggttatggcccagggtttccagt
ttgacctggaaactccccttccagttggtgaaacagtgtttggggacagtcagcctggacccgtccgatgaccagtttgacataaactgtaatgaggtgagtggccccttctgtgttgggcatatgctaac
aatgtgggttgagaaacatcctttggaccaatgacccgctgctccatgtagggcagagaaaaggccctcctacccgggcccaccctccccagtccccaggcctccagccctggctctgcatccctaaagag
aagtggctgtctaacagggtccccacccgggaactgacagtaaggcagattctcagccccactgagacctcctgaatcagactttggggtgggacccaggcatttgtattttctcaaggcctccaggggat
tctgacagtgctgaagttgtgacaccctgactcgagtcatggtcttgaggccatgctccagtccatctttgcccagatgggcttgtgacccttggaagccccttgtcatctctgggatcagtttccccata
tgttgtgggtttaggaattcaaccacatgccccaaagatcaccgccagaagatgatctggagccaaagttcctttggtggctcagtttgggagttgttcaggtggggagagagtggtcttctcttgtctct
tgcccaatcccacaagtaatctcttccattgtggttcacagcttcagagtgtcagggctagacgacctcggcctcccaaacctcggcctcctcacaggccaaggccaaggccactgccatggttcctgcca
aggttcctgggaaaacggtga

Premature termination codon in 2nd exon + NMD

Oryx dammah

VEDREA.KPVSFKVKETVCPRLSQQPLEQCDFKENGLVKQCLGTVSLDPSDDQFDINCNELQSVRARRPRPPKPRPPHRPRPRPLPWFPPRFLGKR

GenBank: JABAEV010001482.1

contig_start_ctgccagcagcgctgctgtgagggccgcttctgctctctgtgtgcccatgaggccgggcacgggctctgtgccctcccctgtgctcctagcaccaagcccagggccggacacacaggg
ggctggagaggctgctgtccgggtaggggacagggagacagatgagagaaggaaacatgagcccgagccaagtctccccactttgatcgttgaccaggtggaggaccgggaagctttgaaagcctgtgagct
tcaaggtgaaggagactgtgtgccccaggctgagccagcagcccctggagcagtgtgacttcaaggagaatggggtgagcctgggggcagggagtgagggctgggatcaatgcttctcagtactagctgaa
cagggaacttcagggacgatttccagcccctggcgggtgaggtgggctgagcctgggaggttatggcccagggtttccagtttgacctggaaactccccttccagttggtgaaacagtgtttggggacagt
cagcctggacccgtccgatgaccagtttgacataaactgtaatgaggtgagtggccccttctgtgttgggcatatgctaacaaggtgggttgagaaacatcctttggaccaatgacccgctgctccatgta
gggcagagaaaaggccctcctacccgggcccaccctccccagtccccaggcctccagccctggctctgcatccctaaagagaagtggctgtctaacagggtccccacccgggaactgacagtaaggcagat
tctcagccccactgagacctcctgaatcagactttggggtgggacccaggcatttgtatttctcaaggcctccaggggattctgacagtgctgaagttgtgacaccctgactcgagtcatggtcttgagg
ccatgctccagtccatctttgcccagatgggcttgtgacccttggaagccccttgtcatctctgggatcagtttccccatatgttgtgggtttcggaattcaaccacatgccccaaagatcaccgccagaa
gttgatctggagccaaagttcctttggtggctcagtttgggagttgttcaggtggggagagagtggtcttctcttgtctcttgcccaatcccacaagtaatctcttccattgtggttcacagcttcagagt
gtcagggctagacgacctcggcctcccaaacctcggcctcctcacaggccaaggccaaggccactgccatggttcccgccaaggttcctgggaaaacggtga

Premature termination codon in 2nd exon + NMD

Addax nasomaculatus

VEDREA.KPVSFRVKETVCPRLSQQPLEQCDFKENGLVKQCLGTVSLDPSDDQFDINCNELQSVRARRPRPPKPRPPHRPRPRPLPWFPPRFLGKR

GenBank: JAIEZW010078559.1

contig_start_gccgctcctgctctctctgtgcccgtgaggccgggcacgggctctgtgccctcccctgtgctcgcagcaccaagcccagggccggacacacaggggctggagaggctgctgtccggg
taggggcagggagacagatgagagaaggaaacatgagcccgagcccagtctccccactttgatcgttgaccaggtggaggaccgggaagcttgaaagcctgtgagcttcagggtgaaggagactgtgtgc
cccaggctgagccagcagcccctggagcagtgtgacttcaaggagaatggggtgagcctgggggcagggagtgagggctgggatcaatgcttctcagtgctagctgaacagggaacttcagggacgatttc
cagcccctggcgggtgaggtgggctgagcctgggaggttatggcccagggtttccagtttgacctggaaactccccttccagttggtgaaacagtgtttggggacagtcagcctggacccgtccgatgacc
agtttgacataaactgtaatgaggtgagtggccccttctgtgttgggcatatgctaacaggtgggttgagaaacatcctttggaccaatgacccgctgctccatgtagggcagagaaaggccctcctac
ccgggcccaccctccccagtccccaggcctccagccctggctctgcatccctaagagaagtggctgtctaacagggtccccacccgggaactgacagtaaggcagattctcagccccactgagacctcctg
aatcagactttgggtgggacccaggcatttgtattttctcaaggcctccaggggattctgacagtgctgaagttgtgacaccctgactcgagtcatggtcttgaggccatgctccagtccatctttgccc
agatgggcttgtgacccttggaagccccttgtcatctctggatcagtttccccatatgttgtgggtttaggaattcaaccacatgccccaaagatcaccgccagaagatgatctggagccaaagttcctt
tggtggctcagtttgggagttgttcaggtggggagagagtggtcttctcttgactcttgcccaatcccacaagtaatctcttccattgtggttcacagcttcagagtgtcagggctagacgacctcggcct
cccaaacctcggcctcctcacaggccaaggccaaggccactgccatggttcccgccaaggttcctgggaaaacggtga

Premature termination codon in 2nd exon + NMD

Odocoileus hemionus

VKTQRASLSLGRWSLWLLRLRLVVPSACAQALSYREAVLRAVDRISDGSTEANLDCLLELDSPPKDVEDHSARKPVSFRVKETVCPRTSQQPPEQCDFKENGLVK.CVGTASLDLSDDQFDLNCNELQSVR
AKRPQLPKAQPPHRPRPRPLSWFLSRFPGKW

GenBank: JAJLRB010001885.1

gtgaagacccagagggccagcctttccctgggacggtggtcactgtggctactgcggctgagactagtggtgccctcggcctgtgcccaggccctcagctacagggaggctgtgcttcgcgctgtggatcg
catcagtgatggctccacagaagctaatctcgactgcctcctggagctagactcacctcccaaggatgtgagttggggaggggctgagccatctcccccagccttggtcacattatccctcccttcgct
caggctgtacctcctgtcaggaaggcactttgccctctaggtggctcccacatcttccaggaaccttcccagacctgggtcccctgccagccccaggcttcctgccttagcatctctgctgtgggaacag
gcgccctgcacacctggctcccaggacttccgggagctccagggatggaggggtcacaggctctgtgaggggacgtccctgctgaagcccctctgcaccgcggtgtctccctgccagggaggcctctgtc
agcctggaggttccaggtacaagcgctctccctgcaggcggccctgacctcccccagcccctctgtggggaggcgctgccctcagcgctgctgtgcggtccgctcctgctctctgtgagcccgtgaggccg
gggacgggctctgtcccctcccctgggctcccagcaccaagcccgggccgaacacgcaggggctggagaggctgccgtctgggtgtgggcagggagacagatcagagaaggaaacttgagcccgagccca
gtctccccactttgatctttgaccaggtggaggaccacagtgctcgaaagcctgtgagcttcagggtgaaggagaccgtgtgccccaggacgagccagcagcccccggagcagtgtgacttcaaggagaat
ggggtgagcctgggtgctgggataaatgcttctcagcacaagctaagcaggggccttcagggaagatttccagcccctgaggggtgaggttggctgaggctggaagtttatggccccaggggttccagttt
gacctggaaactccccttacagctggtgaaatagtgtgtggggacagccagcctggacctgtctgatgaccagtttgacctaaactgtaatgagatgagtggccccttctgtgttgtgcagatgctaacag
ggtaagttgtggaacatcctttggaccaatgacctgatgccccatccagggcagagaaaggccctcctacccagacccaccctccccaatccccaggtctccagccccggctctgtatcctttagagaag
tggctgtctaaaggggtcctcacccaggaactgacagtaaagcagattctcagccctactgagatctcgtgaatcagactttggggtggggcccaggcatttgtattttcacaggcctccaggggattct
gacagtgctgaagttgtgacaccctgactcgagtcatggtcttgaggccatgctccagtccatctttgcccagatgggcttgtgacatttggaagctccttgtcatctctgggatcagtttccccatatgt
tgtgggtttaaggattcaaccacatgctccaaagatcaccgccagaggatgagctggggccaaagttcctttggtggcccagtttgggagttgttcaggtggggagagagtggccttctcttgacccttgc
ccagtcccacaagtaatctcttccactgtggttcacagcttcagagtgtcagggctaaaagacctcagcttcccaaagctcagcctcctcacaggccaaggccaaggccattgtcatggttcctgtcaagg
ttccctggaaaatggtga

Start Met codon mutation

Premature termination codon in 3rd exon + NMD

Intron retention in 3rd exon

Odocoileus virginianus

METQRASLSLGRWSLWLLRLRLVVPSACAQALSYREAVLRAVDRISDGSTEANLYCLLELDSPPKDVEDHSARKPVSFRVKETVCPRSSQQPPEQCDFKENGLVK.CVGTASLDRSDDQFDLN..DLQSVR
AKRPQLPKPQPPHRPRPRPLSWFLSRFPGKW

GenBank: MLBE01000123.1

atggagacccagagggccagcctctccctgggacggtggtcactgtggctactgcggctgagactagtggtgccctccgcctgtgcccaggccctcagctacagggaggctgtgcttcgcgctgtggatcg
catcagtgatgggtccacagaagctaatctctactgcctcctggagctagactcacctcccaaggatgtgagttggggaggggctgagccatctcccccagccttggtcatattatccctcccttcgct
caggctgtacctccggtcaggaaagcacttctccctctaggtggctcccacatcttccaggaaaccttcccagacctgggtcccctgccagccccaggcttcctgccttagcatctctgtagtccagtctc
cccactttgatctttgaccaggtggaggaccacagtgctcgaaagcctgtgagcttcagggtgaaggagaccgtgtgccccaggtcgagccagcagcccccggagcagtgtgacttcaaggagaatggggt
gagcttggggggctggaggctgagggctgggataaatgcttctcagcacaagctaagcaggggccttcagggaagatttccagcccctgaggggtgaggttggctgaggctggaaggttatggccccgggggg
ttccagtttgacctggaaactccccttacagctggtgaaatagtgtgtggggacagccagcctggaccggtctgatgaccagtttgacctaaactaatgagatgagtggcccccttctgtgttgtgcagatg
ctaacagggtaagttgtggaacatgctttggaccaatgacctgatgccccatctagggcagagaaaaggccctcctacccagacccaccctccccaatccccaggtctccagccccggctctgtatcctttt
agagaagtggctgtctaaaggggtcctcacccaggaactgacagtaaagcagattctcagccctactgagatctcgtgaatcagactttggggtggggcccaggcatttgtattttcacaaggcctccagg
ggattctgacagtgctgaagttgtgacaccctgactcgagtcatggtcttgaggccatgcaccagtccatctttgcccagatgggcttgtgacatttggaagctccttgtcatctctgggatcagtttccc
catatgttgtgggtttaaggattcaaccacatgctccaaagatcaccgccagaggatgagctggggccaaagttcctttggtggcccagtttggggggttgttcaggtggggagagagtggtcttctcttga
cccttgcccagtcccacaagtaatctcttccactgtggttcacagcttcagagtgtcagggctaaaagacctcagcttcccaaacctcagcctcctcacaggccaaggccaaggccattgtcatggttctt
gtcaaggttccccggaaaatggtga

Premature termination codons in 3rd exon + NMD

Intron retention in 3rd exon

Antidorcas marsupialis

METQRASLSLGRCSLWLLLLGLVVPSASAQAPSYREAVLRAVDRINDGSTEANLYRLLELDPLPKDVEDRGARKPVSFRVKETVCPRPSLQPVEQCDFKENGLVKQCVGTVSLDPANDQFDLNCNELQSVR
SRRWPSKPRRPHRPRRRPRPWFPPRFPGKW

GenBank: SJYB01007930.1

atggagactcagagggccagcctctcgctgggacggtgttcgctgtggctcctgctgctgggactagtggtgccctcggccagcgcccaggcccccagctacagggaggccgtgcttcgtgctgtggatcg
catcaatgatgggtccacagaagctaatctctaccgcctcctggagcttgacccgcttcccaaggactgagttggagagggagctgagccatctccccctagccttggccacactgtcgcccttccctca
ggctggtcctcctgtcaggaaggctctttccctctaggcgggctcccacctcttccaggaaaccttcccagacctgggtcatctcccagcaccaggcttcctgccttagcatctctgctgtgggatcagg
cgccctgcacacctggctcaggctcccgggacttctgggagctccagggatggagggtcacaggctctgtgaggtgacttccctgctaatgccccctgcccctccgtgtctccctgccaggagggcctc
tgccagcctggaggctccagtgacaagggctctccctgcaggcggccctgacctcccggggcccctctgaggggaggcgctgccagcagcgctgctgtgagggccgcccctgctctctgtgtgcccgtgag
gccgggcacgggctctgggccctcccccgtgctcccagcaccaagcccagggccggacacacagggggctggagaggctgccgtctgggtgggggcagggagacagatcagagaagggaacatgagccga
gcccagtctccccactttgatcgttgaccaggtggaggaccggggagctcgaaagcctgtgagcttcagggtgaaggagactgtgtgccccaggccgagcctgcagcccgtggagcagtgtgacttcaagg
agaatggggtgagcctggggacggggagtgaggcctgggatcagtgcttctcagggagctgaacagggaacttcagggacgatttccagcccctgggggtgaggtgggctgagcctggaaagttatggcc
tggggtttccagtttgaccttgagctccccttgcagctggtgaaacagtgtgtggggacagtcagcctggacccggccaatgaccagtttgacctaaactgtaatgaggtgagtggccccttctgtgttgg
gcagctgctaaaaagggtgggttgagaagcatcctttggaccaatgacccgctgctccgtctagggcagagagaaggccctcctgcctgggcccaccctccccagtccccaggcctccagccctggccctg
catcccttacagaagtgtttgtctactggggtccccacccaggaactgacagtaaggcagattctcagccccgctgagacctcctgaatcagactttggggtgggacccgggaatttgtattttcacaaga
cctccagcggattctgacagtgttgaaattgtgacaccctgactcgagtcatggtcttgaggccatgctccagtccatctttgcccacatgggcttgtgacccttggaagccccttgtcatctctgggatc
agtttccccatatgttgtgggtttaggaattcaaccacatgccccaaagatcactgccagaggatgacctggggccaaagttcctttggtggctcagtttggggggttgttcaggtggggagagagtggtct
tctcttgacccttgcccagtcccacaagtaatctcttccactgtggttcacagcttcagagtgtcaggtctagacgatggccttccaaacctcggcgtcctcacaggccacgccgaaggccacggccatgg
ttcccaccaaggttccccggaaaatggtga

Intron retention in 1st exon

Intron-retaining mediated premature termination codon + NMD

Nanger granti

METQRASLSLGRCLLRLLLPGLVVPSASAQAPSYREAVLRAVDRINDGSTEANLYCLLELDLPPKDV.DRDARKPASFTVKETVCPRPSLQPSEQCDFKENGLVKQCVGTVSLDPADDQFDLNCNELQSVR
SRRQPPKPRGPHRPRPRPRPWFPPRFPGKR

GenBank: SJYD01055729.1

atggagacccagagggccagcctctccctgggacggtgtttgctgcgcctcctgctgccgggactagtggtgccctcggccagcgcccaggcccccagctacagggaggccgtgcttcgtgctgtggatcg
catcaatgatgggtccacagaagctaatctctattgcctcctggagctagacttgcctcccaaggacgtgagttggggaggggctgagccatctccccccagccttggccacactgtcgtcccttcgctc
aggctggtccttctgtcaggaaggctcttttccctctaggcgggctcccacctcttccaggaaaccttcccagacctgggtcatctcccagcaccaggcttcctgcctcagcatctctgctgtgggaacag
gcgccctgcacacctggctcaggctcccgggacttctgggagctccagggatggaggggtcacaggctctgtgaggtgacttccctgctaatgccccctgcacctcggtgtctccctgccaggagggtctc
tgtcagcctgcaggctccagtgacaagggctctccctgcaggcggccctgacctccctgggcccctctgaggggaggtgctgccagcagcgctgctgtgagggccgctcctgctctctgtgtgcccgtgag
gccgggcacgggctctgtgccctcccctgtgctcccagcaccaagcccagggccagacacacaggggggctggagaggctgccgtctgggttgggggcagggagacagatcagagaagggaacatgagccga
gcccagtctccccactttgatcgctgaccaggtttaggaccgggacgctcgaaagcctgcaagcttcacggtgaaggagactgtgtgccccaggccgagcctgcagccctcggagcagtgtgacttcaagg
agaatggggtgagcctgggggcggggagtgagggccgggatcagtgcttctcagtgcaagctgaacagggaagttcagggaagatttccagcccctgggggtgaggtgggctgagcctgggaggttatgg
ccctgggtttccattttgaccttgagctccccttgcagctggtgaaacagtgtgtggggacagtcagcctggacccagccgatgaccagtttgacctaaactgtaatgaggtgagtggccccttctgtgtt
ggggagatgctaaagaaggtgggttgagaaacatcctttggaccaatgacccgctgctctgtctagggcagagagaaggccctcctacctgggcccaccctcccccgtccccaggtctccagccctggctc
tgcatcccttacagaagtgtctgtctaatggggtccccacccaggaactgacagtaaggcagattctcagccccactgagacctcttgaatcagactttggggtgggacccaggcatttgtattttcacaa
gacctccagcgaaagtgttgaagttgtgacaccctgactcgagtcatggtcttgaggccatgctccagtccatctttgcccacatgggcttgtgacccttggaagccccttgtcatctctgggatcagttc
ccccatatgttgtgggtttaggaattcaaccacatgtcccaaagatcactgccagaggatgacctggggccaaagttcctttggtggctcagtttgggagttgttcaggtgaggagagagtggtcttctct
tgacccttgcccagtcccacaagtaatctcttccattgtggttcacagcttcagagtgtcaggtctagacgacagcctcccaaacctcggggtcctcacaggccacgcccaaggccacggccatggttccc
accaaggttccccggaaaacggtga

Premature termination codon in 2nd exon + NMD

Nanger dama

METQRASLSLGRCLLWLLLPGLVVPSASTQALSYREAVLRAVDRINDGSTEANLYCLLELDSPPKDV.DRDARKPASFRVKETVCPRLSLQPSEQCDFKENGLVKQCVGTVSLDPADDQFDLNCNELQSVK
SRQQPPKPRGPHRPRPRPQPWFPPRFPGKR

GenBank: CAKJTW010000016.1

atggagacccagagggccagcctctccctgggacggtgtttgctgtggctcctgctgccgggactagtggtgccctcggccagcacccaggccctcagctacagggaggccgtgcttcgtgctgtggatcg
catcaatgatgggtccacagaagctaatctctactgcctcctggagctagactcgcctcccaaggacgtgagttggggaggggctgagccatctcccccagccttggccacactgtcgtccctttgctca
ggctggtcctcctgtcaggaaggctcttttccctctaggcgggctcccacctcttccaggaaaccttcccagacctgggtcatctcccagcaccaggcttcctgccttagcatctctgctgtgggaacagg
cgccccgcacacctggctcaggctcccgggacttctgggagccccagggatggaggggtcacaggctctgtgaggtgacttcctgctaatgccccctgcacctcagtgtctccctgccgggagggcctctg
tcaacctggaggctccagtgacaagggctctccctgcaggtggccctgacctccctgggcccctctgaggggaggtgctgccagcagcgctgctgtgagggccgctcctgctctctgtgtgcccgtgaggc
cgggcacgggctctgtgccctcccctgtgctcccagcaccaagcccagggccagacacacaggggctggagaggctgccgtctgggttgggggcagggagacagatcagagaagggaacatgagcccgag
cccagtctccccccactttgatcgctgaccaggtttaggaccgggacgctcgaaagcctgcaagcttcagggtgaaggagactgtgtgccccaggctgagcctgcagccctcggagcagtgtgacttcaagg
agaatggggtgagcctggggtggggagtgagggccgggatcagtgcttctcagtgcaagctgaacagggaagttcagggaagatttccagcccctggggggtgaggtgggctgagcctgggaggttatggc
cctgggtttccatttgaccttgagctccccttgcagctggtgaaacagtgtgtggggacagtcagcctggacccagccgatgaccagtttgacctaaactgtaatgaggtgagtggccccttctgtgttg
ggcagatgctaaagaaggtgggttgagaaacatcctttggaccaatgacccgctgctctgtctagggcagagagaaggccctcctacctgggcccaccctcccccgtccccaggtctccagccctggctc
tgcatcccttacagaagtgtctgtctaatggggtccccacccaggaactgacagtaaggcagattctcagccccactgagacctcctgaatcagactttggggtgggacccaggcatttgtattttcacaa
gacctccagcgaaagtgttgaagttgtgacaccctgactcaagtcatggtcttgagggcatgctccagtccatctttgcccacatgggcttgtgacccttggaagccccttgtcatctctgggatcagttc
cccatatgttgtgggtttaggaattcaaccacatgccccaaagatcactgccagaggatgacctggggccaaagttcctttggtggctcagtttaggggttgttcaggtgaggagagagtggtcttctctt
gacccttgcccagtcccacaagtaatctcttccattgtggttcacagcttcagagtgtcaagtctagacaacagcctcccaaacctcggggtcctcacaggccacgcccaaggccacagccatggttccca
ccaaggttccctggaaaacggtga

Premature termination codon in 2nd exon + NMD

Eudorcas thomsonii

METQRASLSLGRCLLWLLLLGLVLPSASAQALSYREAVLRAVDRINDGSTEANLYCLLELDSSPKDV.DRDTRKPASFTVKETVXPRPSLQPVEQCDFKENGLVKQCVGTVSLDPADDQFDLNCNELQSVR
SRRRPPKPRGPHRPRPRPWPWFPPRFPGKQ

GenBank: SJYE01122334.1

atggagacccagagggccagcctctccctgggacggtgtttgctgtggctcctgctgctgggactagtgctgccctcggccagcgcccaggccctcagctacagggaggctgtgcttcgtgctgtggatcg
catcaatgatgggtccacagaagctaatctctactgcctcctggagctagactcgtctcccaaggacgtgagttggggagggggctgagccatctcccccagccttggccacagtgtcgtcccttggctc
aggctggtcctcctgtcaggaaggctcttttccctctaggcggctcccacctcttccaggaaaccttcccagtcctgggtcatctcctagcaccaggctcctgcctcagcatctctgctgtgggatcagg
cgccccgcacacctggctcaggctcccgggacttctgggagccccagggatggaggggtcacaggctctgtgaggtgacttccctgctaatgccccctgcacctcggtgtctccctgccgggagggcctct
gtcagcctggagggctctccctgcaggcggccctgacctccccgggcccctctgaggggaggcgctgccagcagcgctgctgtgagggccgctcctgctctctgtgtgcccgtgaggccgggcacgggctc
tgcgccctcccctgtgctccaagcaccaagcccagggccggacacacaggggggctggaggctgctgtccgggtgggggcagggagacagatcagagaaggaacatgagcccgagcccagtctcccca
ctttgatcgctgaccaggtttaggaccgggacactcgaaagcctgcaagcttcacggtgaaggagactgtgxxccccaggccgagcctgcagcccgtggagcagtgtgacttcaaggagaatggggtgagc
ctgggggcggggagtgagggccgggatcagtgcttctcagtgcaagctgaacagggaagttcagggaagatttccagcccctggggggtgaggtggactgagcctgggaggttatggccctgggtttccat
tttgaccttgagctccccttgcagctggtgaaacagtgtgtggggacagtcagtctggacccagccgatgaccagtttgacctaaactgtaatgaggtgagtggccccttctgtgctgggcagatgctaaa
gaaggtgggttgagaaacatcctttggaccaatgacctgctgctctgtctagggcagagagaaggccctcctacctgggcccaccctccccagtccccaggtctccagccctggctctgcatcccttacag
aagtgtctgtctaatggggtccccacccaagaactgacagtaaggcagattctcagccccactgagacctcctgaatcagactttggggtgggacccatgcatttgtattttcacaagacctccagcgaaa
gtgttgaagttgtgacaccctgactcgagtcatggtcttgaggccatgctccagtccatctttgcccacatgggcttgtgacccttggaagccccttgtcatctctgggatcagttcccccatatgttgtg
ggtttaggaattcaaccacatgccccaaagatcactgccagaggatgacctggggccaaagttcctttggtggctcagtttggggggttgttcaggtgaggagagagtggtcttctcttgacccttgcccag
tcccacaagtaatctcttccattgtggttcacagcttcagagtgtcaggtctagacgacggcctcccaaacctcggggtcctcacaggccacgcccaaggccatggccatggttcccaccaaggttccccg
gaaaacagtga

Indel (2 bp deletion in 1st exon)

Premature termination codon in 2nd exon + NMD

Ourebia ourebi

EQCDFKENGLVKQCVGTVSLDLSDAQFDLNCNELQSVRSRRRPSNLRYPHRPRQRPRPWFPPRFPGKR

GenBank: SJXZ010154060.1, SJXZ012697268.1

contig_start_ggagcagtgtgacttcaaggagaatggggtgagtctgggggcggggagtgagggctgggatcagtgcttctcagtgcaagctgaacagggagcttcagggacgatttccagcccctgg
ggggtgaggtgggctgagcctgggaggttatggccctgggtttccagtttgaccttgagccccccttgcagctggtgaagcagtgtgtggggacagtcagcctggacctgtccgatgcccagtttgacctg
aactgtaatgaggtgagtggccccttctgtgttgggccgatgctaaaagaggtgggttgagaagcatcccttggaccaatgacccgctgctccgtctagggcagagagaaggccctcctacctgggcccac
cctccccagtccccaggtctccagccctggctctgcgtcccttacagaagtgtctgtctaatggggtccccacccaggaactgacagtaaggcagattctcagccccactgagacctcctgaatcagactt
tggggtgggacccaggcatttgtattttcacaggacctccaggggactctgacagtgctgaagttgtgacaccctgactcgagtcgtggtcttgaggccatgctccagtccatctttgcccacatgggctt
gtgacccttggaagccccttgtcatctctgggatcagtttccccatatgttgtgggtttaggaattcaaccacatgccccaaagatcactgccagaggaagacgtggggccaaagttcctttggtggctca
gtttggggggttgttcaggtcgggagagaatggtcttctcttgacccttgcccagtcccacaagtaatctcttccattgtggttcacagcttcagagtgtcaggtctagacgacggccttccaatcttcggt
atcctcacaggccacggcaaaggccacggccatggttcccgccaaggttccccggaaaacggtga

Axis porcinus

VKTQRASFSLGRWSLWLLRLRLVLPLASAQALSYREAVLHTGDCINDGSTEANLYRLLELDPPPEEDVEDHSARKPVSFRVKETICPRTSQQPLEQCDFRENGLVKQCVGTASLDRSDDQFDLNCNELQSV
RAKRPQPPKPRPPHRPRPRPRSWFLPRFPGKR

GenBank: QQTR01119330.1

gtgaagacccagagggccagcttctccctgggacggtggtcactgtggctactgcggctgagactagtgctgcccttggcctctgcccaggccctcagctacagggaggccgtgcttcacactggggattg
cattaatgacgggtccacagaagctaatctctaccgcctcctggagctagacccgcctcctgaggaggatgtgagttggggagggggctgagccatctccccccagccttggtcacattatccctcccttc
gctcaggctgtacctcctgtcaggaaggcactttgccctctaggtgggcttccacctcttccagaaaaccttcccagacctcccagccccaggcttcctgccttagcatctctgctgcgggatcaggcgcc
ctgcacacctggctcccaggacttccgggagctccagggatggaggggggtcacaggctctgtgaggtgatgtccctgctgaagccccctctgcgctgtggtgtctccctgccagggaggcctctgtcagcc
tggaggttccaggtacaagggctctccctgcaggcggccctgacctccctcagcccctctgcggggaggcgctgccctcagcgctgccatgcggtccgctcgtgctctctgggtgcccatgaggccgggga
tgggctctgcccctcccctgggctcccagcaccaagcccagggccggacacacaggggggctggagaggctgctgtctggatgggggcggggagacagatcagagaaggaaacatgagcccgagcccagtcc
cgccactttgatctttgaccaggtggaggaccacagtgctcgaaagcctgtgagcttcagggtgaaggagacgatatgccccaggacgagccagcagcccctggagcagtgtgacttcagggagaatgggg
tgagcctgggggctggaggctgagggctgggataaatgcttctcagcacaagctgagcaggggacttcagggaagatttccagcccctgaagggtgaggttggctgagcctggaaggttatggccccgggg
gttccagtttgacctggaaactccccttccagctggtgaaacagtgtgtggggacagccagcctggaccggtctgatgaccagtttgacctaaactgtaatgaggtgagtggccccttctgtgttgtgcag
atgctaacagggtagattgtggaacatcctttggaccaatgacccgatgccccatccaggtcagagaaaaggccctcctacccaggcccaccctccccaatccccagctctccagccctggctctgtatcc
tttagagaagtggctgtctaaaggggtcctcacccaggaactgacagtaaggcagattctcagccctactgagatctcccgaaccagactttggggtggggcccaggcatttgtattttcacaaggcctcc
aggggattctgacagtgctgaagttgtgacaccctgactcgagtcatggtcttgaggacatgctccagtccatctttgcccggatgggcttctgacacttggaagctccttgtcatctctgggatcagttt
ccccatatgttgtgggtttaaggattcaaccacatgctccaaagatcaccgccagaggatgagttggggccaaagttcctctggtggcccagtttggggggttgttcaggtggggagagagtggtcttctct
tgacccttgcccagtcccacaagtaatcgcttccattgtggttcacagcttcagagtgtcagggctaaacgacctcagcctcccaaaccccggcctcctcacaggccaaggccaaggccacggtcatggtt
cctgccaaggttccccggaaaacggtga

Start Met codon mutation

Cervus elaphus (hippelaphus)

KQRASLSLGRWSLWLLQLRLVLPSASAQALSYREAVLHTGDCINDGSTEANLYRLLELDPPPEQDMEDRGARKPLSFTVKETVCPRTSQQPPEQCDFKENGLVKQCVGTASLDRSDDQFDLNCNELQSVRA
KGPQPPKPRPPHRPRPRPRSWFLPRFPGKQ

GenBank: MKHE01000024.1

aagagtcagagggccagcctctccctgggacggtggtcactgtggctactgcagctgagactagtgctgccctcggcctctgcccaggccctcagctacagggaggccgtgcttcacacggggactgcat
caatgacgggtccacagaagctaatctctaccgcctcctggagctagacccgcctccagagcaggacgtgagttggggagggggctgggaaggggatctgtctcctgacacccttggccacactgtcgccc
tcttcactcaggctgtacctcctgtcaggaaggcactttgccctctaggtggctcccacctcttccagaaaccttcccagacctgggtcccctgccagccccaggcttcctgccttagcatctctgctgt
gggatcaggcgccctgcacacctggctcccaggacttccgggagctccagggatggaggggtcacaggctctgtgaggtgatgtccctgctgaagcccctctgcactgcggtgtctccctgccagggagg
cctctgtcagcctggagattccaggtacaagggctctctctgcaggcggccctgacctccctcagcccctctgcggggaggctctgccctcagcgctgccgtgcggtctgctcctgctctctgtgagccca
tgaggcgggggacgggctctgtccctcccctgggctcccagcaccaagcccagggccggacacacaggggggctggagaggctgccgtccgggtggggacagggagacagatcagagaaggaaacatgagcc
tgagcccagtccccccactttgatctttgaccagatggaggaccggggagctcgaaagcctctgagcttcacggtgaaggagaccgtgtgtcccaggacgagccagcagcccccagagcagtgtgacttca
aggagaatggggtgagcctgggggctggaggctgagggctgggataaatgcttctcagcacaagctgagcaggggacttcagggaagatttccagcccctgaagggtgaggttggctgagcctggaaggtt
atggccccgggggttccagtttgacctggaaactccccttccagctggtgaaacagtgtgtggggacagccagcttggaccggtctgatgaccagtttgacctaaactgtaatgaggtgagtggcccttc
tgtgttgtgcagatgctaacagggtaggttgtggaacatcctttggaccaatgacccgatgccccatccagggcagagaaaaggccctcctacccaggcccaccctccccaatccccaggtctccagccct
ggctctgtatcctttagagaagtggctgtctaaaggggtcctcacccaggaactgacagtaagggagattctcagccctactgagatctcctgaatcagactttggggtggggcccaggcatttgtatttt
cacaaggcctccaggggatcctgacagtgctgaagttgtgacaccctgactcgagtcatggtcttgaggccatgctccagtccatctttgcccggatgggcttgtgacatttagaagctccttgtcatctc
tgggatcagtttccccatatgttgtgggtttaaggattcaagcacatgctccaaagatcactgtcagaggatgagctggggccaaagttcctttggtggcccagtttggggggttgttcaggtggggagaga
gtggtcttctcttgacccttgtccagtcccacaagtaatctcttccattgtggttcacagcttcagagtgtcagggctaaaggacctcagcctcccaaacctcggcctcctcacaggccaaggccaaggcc
acggtcatggttcctgccaaggttccccggaaaacagtga

Start Met codon mutation

Cervus nippon

KSQRASLSLGRWSLWLLQLRLVLPSASAQSLSYREAVLHTGDCINDGSTEANLYRLLELDPPPEQDMEDRGARKPLSFTVKETVCPRTSQQPPEQCDFKENGLVKQCVGTASLDRSDDQFDLNCNELQSVR
AKGPQPPKPRPPHRPRPRPRSWFLPRFPGKQ

GenBank: JAOBNA010000016.1

aagagtcagagggccagcctctccctgggacggtggtcactgtggctactgcagctgagactagtgctgccctcggcctctgcccagtccctcagctacagggaggccgtgcttcacacggggactgcat
caatgacgggtccacagaagctaatctctaccgcctcctggagctagacccgcctccagagcaggatgtgagttggggaggggctgggaaggggatctgtctcctgacacccttggccacactgtcgccc
ccttcactcaggctgtacctcctgtcaggaaggcactttgccctctaggtggctcccacctcttccagaaaaccttcccagacctgggtcccctgccagccccaggcttcctgccttagcatctctgctgt
gggatcaggcgccctgcacacctggctcccaggacttccgggagctccagggatggaggggtcacaggctctgtgaggtgatgtccctgctgaagcccctctgcactgcggtgtctccctgccagggagg
cctctgtcagcctggaggttccaggtacaagggctctccctgcaggcggccctgacctccctcagcccctctgcggggaggcgctgccctcagcgctgccgtgcggtccgctcctgctctctgtgagcccg
tgaggcgggggacgggctctgtccctcccctgggctcccagcaccaagcccagggccagacacgcaggggctggagaggctgccgtctgggtggggcagggagacagatcagagaaggaaacatgagcc
cttgcccagtccccccactttgatctttgaccagatggaggaccggggagctcgaaagcctctgagcttcacggtgaaggagaccgtgtgtcccaggacgagccagcagcccccagagcagtgtgacttca
aggagaatggggtgagcctggggctggaggctgagggctgggataaatgcttctcagcacaagctgagcaggggacttcagggaagatttccagcccctgaagggtgaggttggctgagcctggaaggtt
atggccccggggggttccagtttgacctggaaagtccccttccagctggtgaaacagtgtgtggggacagccagcttggaccggtctgatgaccagtttgacctaaactgtaatgaggtgagtggcccctt c
tgtgttgtgcagatgctaacagggtaggttgtggaacatcctttggaccaatgacccgatgccccatccagggcagagaaaaggccctcctacccaggcccaccctccccaatccccaggtctccagccct
ggctctgtatcctttagagaagtggctgtctaaaggggtcctcacccaggaactgacagtaagggagattctcaggcctactgagatctcctgaatcagactttggggtggggcccaggcatttgtacttt
cacaaggcctccaggggatcctgacagtgctgaagttgtgacaccctgactcgagtcatggtcttgaggccatgctccagtccatctttgcccggatgggcttgtgacatttggaagctccttgtcatctc
tgggatcagtttccccatatgctgtgggtttaaggattcaaccacatgctccaaagatcactgtcagaggatgagctggggccaaagttcctttggtggcccagtttggggggttgttcaggtggggagaga
gtggtcttctcttgacccttgtccagtcccacaagtaatctcttccattgtggttcacagcttcagagtgtcagggctaaaggacctcagcctcccaaacctcggcctcctcacaggccaaggccaaggcc
acggtcatggttcctgccaaggttccccggaaaacagtga

Start Met codon mutation

Elaphurus davidianus

VKTQRASLSLGRWSLWLLLLGLVLPLASAQALSYREAVLHTGDCINDGSTEANLYCLLELDSPPKDVEDHSARKPVSFRVKETVCPRTSQQPPEQCDFKENGLVKQCVGTASLDRSDDQFDLNCNELQSVR
AKRPQPPKPRPPHRPRPRPWSWFLPRFPGKW

GenBank: JRFZ01039796.1

gtgaagacccagagggccagcctctccctgggacggtggtcactgtggctactgctgctgggactagtgctgcccttggcctctgcccaggccctcagctacagggaggccgtgcttcacactggggattg
catcaatgatgggtccacagaagctaatctctactgcctcctggagctagactcacctcccaaggatgtgagttggagaggggctgagccatctcccccaagccttggtcacattatccctcccttcgct
caggctgtacctcctgtcaggaaggcactttgccctctaggtgggctcccacctcttccagaaaccttcccagacctgggtaccctgccagccccaggcttcctgccttagcatctctgctgcgggaaca
ggcgccctgcacacctggctcccaggacttccgggagctccagggatggaggggtcacaggctctgtgaggtgatgtccctgctgaagcccctctgcactgcggtgtctccctgccagggaggcctctgt
cagcctggaggttccaggtacaagggctctccctgcagcccctctgcggggaggctctgccctcagcgctgccgtgcggtccactcctgctctctgtgagcccgtgaggccggggatgggctctgcccctc
ccctgggctcccagcaccaagcccagggccggacacgcaggggctgcagaggctgccatctgggtggggcagggagacagatcagagaaggaaacatgagcccgagtccagtccccccactttgatctt
tgacagagtggaggaccacagtgctcgaaagcctgtgagcttcagggtgaaggagaccgtgtgtcccaggacgagccagcagcccccggagcagtgtgacttcaaggagaatggggtgagcctggggggctg
gaggctgagggctgggataaatgcttctcagcacaagctgagcaggggacttcagggaagatttccagcccctgaagggtgaggttggctgagcctggaaggttatggccccggggggttccagtttgacct
gaaaactccccttccagctggtgaaacagtgtgtggggacagccagcctggaccggtctgatgaccagtttgacctaaactgtaatgaggtgagtggccccttctgtgttgtgcagatgctaacagggtag
gttgtgaaacatcctttggaccaatgacccgatgccccatccagggcagagaaaaggccctcctacccaggcccaccctccccaatccccaggtctccagccctggctctgtatcctttagagaagtggct
gtctaaaggggtcctcacccaggaactgacagtaagggagattctcagccctactgagatctcctgaatcagactttggggtggggcccaggcatttgtattttcataaggcctccaggggatcctgacag
tgctgaagttgtgacaccctgactcgagtcatggtcttgaggacatgctccagtccatctttgcccggatggcttctgacacttggaagctccttgtcatctctgggatcagtttccccatatgttttgg
gtttaaggattcaactacatgctccaaagatcaccgccagaggatgagctggggccaaagttcctttggtggcccagtttggggggttgttcaggtggggagagagtggtcttctcttgacccttgcccagt
cccacaagtaatctcttccattgtggttcacagcttcagagtgtcagggctaaacgacctcagcctcccaaacctcggcctcctcacaggccaaggccaaggccatggtcatggttcctgccaaggttccc
cggaaaatggtga

Start Met codon mutation

Dama dama

VKTQRASLSLGRWSLWLLQLRLVLPFASAQALSYREAVLHTGDCINDGSTEANLYCLLELDSPPKDVEDHSARKPVSFRVKETVCPRTSQQPLEQCDFKENGLVKQCVGTASLDQSDDQ
FDLNCNELQSVRAKRPQPPKPRPPHRPRPRPRSWFLPRFPGKR

GenBank: JASJUW010000017.1

gtgaagacccagagggccagcctctccctgggacggtggtcactgtggctactgcagctgagactagtgctgcccttcgcctctgcccaggccctcagctacagggaggccgtgcttcacactggggattg
catcaatgatgggtccacagaagctaatctctactgcctcctggagctagactcacctcccaaggatgtgagttggagaggggctgagccatctccccccagccttggtcacattatccctcccttcgct
caggctgtacctcctgtcaggaaggcactttgccctctaggtgggctcccacctcttccaggaaaccttcccagacctgggtcccctgccagccccaggcttcctgccttagcatctctgctgtgggatca
ggcgccctgcacacctggctcccaggacttccgggagctccagggatggaggggtcacaggctctgtgaggtgacgtccctgctgaagcccctctgcactgcggtgtctccctcccagggaggcctctgt
cagcctggaggttccaggtacaagggctctccctgcaggcggccctgacctccctcacccctctgtggggaggcgctgccctcagcgctgctgtgcggtccgctcctgctctctgtgagcccgtgaggccg
gggatgggctctgcccctcccctgggctcccagcaccaagcccagggccggacacacaggggggctggagaggctgccatctgggtggggcggggagacagatcagagaaggaaacatgagcccgagtcca
gtcccccccactttgatctttgaccaggtggaggaccacagtgctcgaaagcctgtgagcttcagggtgaaggagaccgtgtgccccaggacgagccagcagcccctggagcagtgtgacttcaaggagaat
ggggtgagcctgggggctggaggctgagggctgggataaatgcttctcagcacaagctgagcaggggacttcagggaagatttccagcccctgaagggtgaggttggctgagcctggaaggttatggcccc
aggggttccagtttgacctgaaaactccccttccagctggtgaaacagtgtgtggggacagccagcctggaccagtctgatgaccagtttgacctaaactgtaatgaggtgagtggccccttctgtgttgt
gcagatgctaacagggtaggttgtggaacatcctttggaccaatgacccgatgccccatccagggcagagaaaggccctcctacccaggcccaccctccccaatccccaggtctccagccctggctctgt
atcctttaagaagtggctgtctaaaggggtcctcacccaggaactgacagtaagggagattctcagccctactgagatctcctgaatcagactttggggtggggcccaggcatttgtattttcacaaggcc
tccaggggatcctgacagtgctgaagttgtgacaccctgactcgagtcatggtcttgaggacatgctccagtccatctttgcccggatggacttctgacacttggaagctccttgtcatctctgggatcag
tttccccatatgttttgggtttaaggattcaaccacatgctccaaagatcaccgccagaggatgagctggggccaaagttcctttggtggcccagtttggggggttgttcaggtggggagagagtggtcttc
tcttgacccttgcccagtcccacaagtaatctcttccattgtggttcacagcttcagagtgtcagggctaaacgacctcagcctcccaaacctcggcctcctcacaggccaaggccaaggccacggtcatg
gttcctgccaaggttccccggaaaacggtga

Start Met codon mutation

Cervus hanglu

KSQRASLSLGRWSLWLLQLRLVLPSASAQALSYREAVLHTGDCINDGSTEANLYRLLELDPPPEQDVEDRGARKPLSFTVKETVCPRTSQQPPEQCDFKENGLVKQCVGTASLDRSDDQFDLNCNELQSVR
AKGPQPPKPRPHHRPRPRPRSWFLPRFPGKQ

GenBank: CM021215.1

aagagtcagagggccagcctctccctgggacggtggtcactgtggctactgcagctgagactagtgctgccctcggcctctgcccaggccctcagctacagggaggccgtgcttcacacggggactgcat
caatgacgggtccacggaagctaatctctaccgcctcctggagctagacccgcctccagagcaggacgtgagttggggaggggctgggaaggggatctgtctcctgacacccttggccacactgtcgccc
ccttcactcaggctgtacctcctgtcaggaaggcactttgccctctaggtggctcccacctcttccagaaaccttcccagacctgggtcccctgccagccccaggcttcctgccttagcatctctgctgt
gggatcaggcgccctgcacacctggctcccaggacttccgggagctccagggatggaggggtcacaggctctgtgaggtgacgtccctgctgaagcccctctgcactgcggtgtctccctcccagggag
gcctctgtcagcctggaggttccaggtacaagggctctccctgcaggcggccctgacctccttcagcccctctgcggggaggcgctgccctcagcgctgccgtgcggtccgctcctgctctctgtgagccc
gtgaggccgggacgggctctgtccctcccctgggctcccagcaccaagcccagggccagacacacaggggctggagaggctgccgtccgggtggggcggggagacagatcagagaaggaaacatgagcc
cgggtccagtctccccactttgatctttgaccaggtggaggaccggggagctcgaaagcctctgagcttcacggtgaaggagaccgtgtgtcccaggacgagccagcagcccccagagcagtgtgacttca
aggagaatggggtgagcctggggctggaggctgagggctgggataaatgcttctcagcacaagctgagcaggggacttcagggaagatttccagcccctgaagggtgaggttggctgagcctggaaggtt
atggccccggggttccagtttgacctggaaactccccttccagctggtgaaacagtgtgtggggacagccagcttggaccggtctgatgaccagtttgacctaaactgtaatgaggtgagtggccccttc
tgtgttgtgcagatgctaacagggtaggttgtggaacatcctttggaccaatgacccgatgccccatccagggcagagaaaggccctcctacccaggcccaccctccccaatccccaggtctccagccct
ggctctgtatcctttagagaagtggctgtctaaaggggtcctcacccaggaactgacagtaagggagattctcagccctactgagatctcctgaatcagactttggggtggggcccaggcatttgtacttt
cacaaggcctccaggggatcctgacagtgctgaagctgtgacaccctgactcgagtcatggtcttgaggccatgctccagtccatctttgcccggatgggcttgtgacatttggaagctccttgtcatctc
tgggatcagtttccccatatgttgtgggtttaaggattcaaccacatgctccaaagatcactgtcagaggatgagctggggccaaagttcctttggtggcccagtttgggggttgttcaggtggggagaga
gtggtcttctcttgacccttgtccagtcccacaagtaatctcttccattgtggttcacagcttcagagtggagtgtcagggctaaaggacctcagcctcccaaacctcggcctcatcacaggccaaggcca
aggccacggtcatggttcctgccaaggttccccggaaaacagtga

Start Met codon mutation

5 bp insertion in 4th exon

Cervus canadensis

KSQRASLSLGRWSLWLLQLRLVLPSASAQALSYREAVLHTGDCINDGSTEANLYRLLELDPPPEQDVEDRGARKPLSFTVKETVCPRTSQQPPEQCDFKENGLVKQCVGTASLDRSDDQFDLNCNELQSVR
AKGPQPPKPRPHHRPRPRPRSWFLPRFPGKQ

GenBank: JACTMD010000173.1

aagagtcagagggccagcctctccctgggacggtggtcactgtggctactgcagctgagactagtgctgccctcggcctctgcccaggccctcagctacagggaggccgtgcttcacacggggactgcat
caatgacgggtccacggaagctaatctctaccgcctcctggagctagacccgcctccagagcaggacgtgagttggggaggggctgggaaggggatctgtctcctgacacccttggccacactgtcgccc
ccttcactcaggctgtacctcctgtcaggaaggcactttgccctctaggtggctcccacctcttccagaaaccttcccagacctgggtcccctgccagccccaggcttcctgccttagcatctctgctgt
gggatcaggcgccctgcacacctggctcccaggacttccgggagctccagggatggaggggtcacaggctctgtgaggtgacgtccctgctgaagcccctctgcactgcggtgtctccctcccagggag
gcctctgtcagcctggaggttccaggtacaagggctctccctgcaggcggccctgacctccttcagcccctctgcggggaggcgctgccctcagcgctgccgtgcggtccgctcctgctctctgtgagccc
gtgaggccgggacgggctctgtccctcccctgggctcccagcaccaagcccagggccagacacacaggggggctggagaggctgccgtccgggtggggcggggagacagatcagagaaggaaacatgagcc
cgggtccagtctccccactttgatctttgaccaggtggaggaccggggagctcgaaagcctctgagcttcacggtgaaggagaccgtgtgtcccaggacgagccagcagcccccagagcagtgtgacttca
aggagaatggggtgagcctggggggctggaggctgagggctgggataaatgcttctcagcacaagctgagcaggggacttcagggaagatttccagcccctgaagggtgaggttggctgagcctggaaggtt
atggccccggggggttccagtttgacctggaaactccccttccagctggtgaaacagtgtgtggggacagccagcttggaccggtctgatgaccagtttgacctaaactgtaatgaggtgagtggcccccttc
tgtgttgtgcagatgctaacagggtaggttgtggaacatcctttggaccaatgacccgatgccccatccagggcagagaaaaggccctcctacccaggcccaccctccccaatccccaggtctccagccct
ggctctgtatcctttagagaagtggctgtctaaaggggtcctcacccaggaactgacagtaagggagattctcagccctactgagatctcctgaatcagactttggggtggggcccaggcatttgtacttt
cacaaggcctccaggggatcctgacagtgctgaagctgtgacaccctgactcgagtcatggtcttgaggccatgctccagtccatctttgcccggatgggcttgtgacatttggaagctccttgtcatctc
tgggatcagcttccccatatgttgtgggtttaaggattcaaccacatgctccaaagatcactgtcagaggatgagctggggccaaagttcctttggtggcccagtttggggggttgttcaggtggggagaga
gtggtcttctcttgacccttgtccagtcccacaagtaatctcttccattgtggttcacagcttcagagtggagtgtcagggctaaaggacctcagcctcccaaacctcggcctcatcacaggccaaggcca
aggccacggtcatggttcctgccaaggttccccggaaaacagtga

Start Met codon mutation

5 bp insertion in 4th exon

Cervus albirostris

KSQRASLSLGRWSLWLLQLRLVLPSASAQALSYREAVLHTGDCINDGSTEANLYRLLELDPPPEQDMEDRGARKPLSFTVKETVCPRTSQQPPEQCDFKENGLVKQCVGTASLDRSDDQFDLNCNELQSVR
AKGPQPPKPRPPHRPRPRPRSWFLPRFPGKQ

GenBank: SJXR01001537.1

```
aagagtcagagggccagcctctccctgggacggtggtcactgtggctactgcagctgagactagtgctgccctcggcctctgcccaggccctcagctacagggaggccgtgcttcacacggggactgcat
caatgacgggtccacggaagctaatctctaccgcctcctggagctagacccgcctccagagcaggacgtgagttggggaggggctgggaaggggatctgtctcctgacacccttggccacactgtcgccc
ccttcactcaggctgtacctcctgtcaggaaggcactttgccctctaggtggctcccacctcttccagaaaccttcccagacctgggtcccctgccagccccaggcttcctgccttagcatctctgctgc
gggaacaggcgccctgcacacctggctcccaggacttccgggagctccagggatggaggggtcacaggctctgtgaggtgacgtccctgctgaagccccctctgcactgcggtgtctccctgccagggag
gcctctgtcagcctggaggttccaggtacaagggctctccctgcaggcggccctgacctccctcagcccctctgcggggaggcgctgccctcagcgctgccgtgcggtccgctcctgctctctgtgagccc
gtgaggcgggggacgggctctgtccctcccctgggctcccagcaccaagcccagggccagacacgcaggggctggagaggctgccgtctgggtgggggcagggagacagatcagagaaggaaacatgagc
ccttgcccagtccccccactttgatctttgaccagatggaggaccggggagctcgaaagcctctgagcttcacggtgaaggagaccgtgtgtcccaggacgagccagcagcccccagagcagtgtgacttc
aaggagaatggggtgagcctggggctggaggctgagggctgggataaatgcttctcagcacaagctgagcaggggacttcagggaagatttccagcccctgaagggtgaggttggctgagcctggaaggt
tatggccccggggggttccagtttgacctggaaactccccttccagctggtgaaacagtgtgtggggacagccagcttggaccggtctgatgaccagtttgacctaaactgtaatgaggtgagtggcccctt
ctgtgttgcgcagatgctaacagggtaggttgtggaacatcctttggaccaatgacccgatgccccatccagggcagagaaaaggccctcctacccaggcccaccctccccaatccccaggtctccagccc
tggctctgtatcctttagagaagtggctgtctaaaggggtcctcacccaggaactgacagtaagggagattctcagccctactgagatctgaatcagactttggggtggggcccaggcatttgtactttca
caaggcctccaggggatcctgacagtgctgaagctgtgacaccctgactcgagtcatggtcttgaggccatgctccagtccatctttgcccggatgggcttgtgacatttggaagctccttgtcatctctg
ggatcagtttccccatatgctgtgggtttaaggattcaaccacatgctccaaagatcactgtcagaggatgagctggggccaaagttcctttggtggcccagtttggggggttgttcaggtggggagagagt
ggtcttctcttgacccttgtccagtcccacaagtaatctcttccattgtggttcacagcttcagagtggagtgtcagggctaaaggacctcagcctcccaaacctcggcctcctcacaggccaaggccaag
gccacggtcatggttcctgccaaggttccccggaaaacagtga
```

Start Met codon mutation

5 bp insertion in 4th exon

Przewalskium albirostris

KSQRASLSLGRWSLWLLQLRLVLPSASAQALSYREAVLHTGDCINDGSTEANLYRLLELDPPPEQDMEDRGARKPLSFTVKETVCPRTSQQPPEQCDFKENGLVKQCVGTASLDRSDDQFDLNCNELQSVR
AKGPQPPKPRPPHRPRPRPRSWFLPRFPGKQ

GenBank: SJXR01001537.1

aagagtcagagggccagcctctccctgggacggtggtcactgtggctactgcagctgagactagtgctgccctcggcctctgcccaggccctcagctacagggaggccgtgcttcacacggggactgcat
caatgacgggtccacggaagctaatctctaccgcctcctggagctagacccgcctccagagcaggacgtgagttggggaggggctgggaaggggatctgtctcctgacaccccttggccacactgtcgccc
ccttcactcaggctgtacctcctgtcaggaaggcactttgccctctaggtggctcccacctcttccagaaaaccttcccagacctgggtcccctgccagccccaggcttcctgccttagcatctctgctgc
gggaacaggcgccctgcacacctggctcccaggacttccgggagctccagggatggaggggtcacaggctctgtgaggtgacgtccctgctgaagcccctctgcactgcggtgtctccctgccagggag
gcctctgtcagcctggaggttccaggtacaagggctctccctgcaggcggccctgacctccctcagcccctctgcggggaggcgctgccctcagcgctgccgtgcggtccgctcctgctctctgtgagccc
gtgaggcgggggacgggctctgtccctcccctgggctcccagcaccaagcccagggccagacacgcaggggctggagaggctgccgtctgggtgggggcagggagacagatcagagaaggaaacatgagc
ccttgcccagtccccccactttgatctttgaccagatggaggaccggggagctcgaaagcctctgagcttcacggtgaaggagaccgtgtgtcccaggacgagccagcagcccccagagcagtgtgacttc
aaggagaatggggtgagcctggggctggaggctgagggctgggataaatgcttctcagcacaagctgagcaggggacttcagggaagatttccagcccctgaagggtgaggttggctgagcctggaaggt
tatggccccggggggttccagtttgacctggaaactccccttccagctggtgaaacagtgtgtggggacagccagcttggaccggtctgatgaccagtttgacctaaactgtaatgaggtgagtggcccctt
ctgtgttgcgcagatgctaacaggtaggttgtggaacatcctttggaccaatgacccgatgccccatccagggcagagaaaaggccctcctacccaggcccaccctccccaatccccaggtctccagccc
tggctctgtatcctttagagaagtggctgtctaaaggggtcctcacccaggaactgacagtaagggagattctcagccctactgagatctgaatcagactttggggtggggcccaggcatttgtactttca
caaggcctccaggggatcctgacagtgctgaagctgtgacaccctgactcgagtcatggtcttgaggccatgctccagtccatctttgcccggatgggcttgtgacatttggaagctccttgtcatctctg
ggatcagtttccccatatgctgtgggtttaaggattcaaccacatgctccaaagatcactgtcagaggatgagctggggccaaagttcctttggtggcccagtttgggggttgttcaggtggggagagagt
ggtcttctcttgacccttgtccagtcccacaagtaatctcttccattgtggttcacagcttcagagtggagtgtcagggctaaaggacctcagcctcccaaacctcggcctcctcacaggccaaggccaag
gccacggtcatggttcctgccaaggttccccggaaaacagtga

Start Met codon mutation

5 bp insertion in 4th exon

Muntiacus muntjak

R.TQRSSFSLGRWSLWLLWLGLVLPSASAQALSYREAVLHTGDCINDRSTEANLYCLLELDSPPKDVEDHSAQKPVSFRVKETVCPKMSQQLPEQCDFKENGLVKQCVGTASLDRSDDQFDLNCNELQSVR
AK.PQPPKPRPPHRPRPRPRSWFLPRFPGKR

GenBank: SJXU01045938.1

cggtgaacccagaggtccagcttctccctgggacggtggtcactatggctactgtggctgggactagtgctgccctcggcctctgcccaggccctcagctacagggaggccgtgcttcacactggggattg
catcaatgacaggtccacagaagctaatctctactgcctcttggagctagactcacctcccaaggatgtgagttggagaggggctgagccatctcccctcagccttggtcacattatccctcccttcgct
catgctgttcctcctgtcaggaaagcacttctccctctaggtgggctctcacctcttcaggaaaccttcccagaactgggaccctcccagccccaggcttcctgccttagcatctctgctgtgggaacagg
tgccctgcacacctggctcccaggacttccgggagctccagggatggaggggtcacaggctctgtgaggtgacgtccctgctgaagcccctctgcactgcggtgtctccctgccagggaggcctctgtca
gctctaggttccaggtacaagggctctccctgcaggcggccccgaactccctcagcccctctgcggggaggcgctgccctcagcgctgctgtgcggtccgctcctgctctctgtgagcccgtgaggccagg
gacggcctctgtccctcccctgggctcccagcaccaagcccagggccggacacgcagggggctggagaggctgccgtctggttgggggcggggagacagatcagagaaggaaagatgagcccgagtccagt
ctccccactttgatctgtgaccaggtggaggaccacagtgctcaaaagcctgtgagcttcagggtgaaggagaccgtgtgccccaagatgagccagcagctcccagagcagtgtgacttcaaggagaatgg
ggtgagcctgggggctggaggctgagggctgggataaatgcttctcagcgcaagctgagcagggggccttcagggaagatttccagcccctgaggggtgaggttgactgaacctgggaggttatggccccgg
gggttccagtttgacctggaaactccccttccagctggtgaaacagtgtgtggggacagccagcctggaccggtccgatgaccagtttgacctaaactgtaatgaggtgagtggccccttctgtgttgtgc
agatgctaacaggataggttgtggaacatcctttggaccaatgacccgctgccccatccagggcagagaaaaggccctcctacctaggcccaccctccccagtctgcaggtctccagccctggctctgtat
cctttagagaagtggctgtctaaaggggtcctcacccaggaactgacattaaggcagattctcagccctgctgagatctcctgaatcagactttggggtggggcccaggcatttgtattttcacaaggcct
ccaggggattctgacagtgctgaagttgtgacaccctgactcgagtcatggtcttgaggccatgctccagtccatctttgcccggatggctgtgacatttggaagctccttgtcatctctgggatcagtt
tccccacatgaggtgggtttaaggattcaaccacatgctccaaagatcaccgccagaagatgagctggggccaaagttcctttggtggcccagtttggggggttattcaggtggggagagagtggtcttctc
ttgacccttgcccagtcccacaagtaatctcttccattgtggttcacagcttcagagtgtcagggctaaatgacctcagcctcccaagcctcggcctcctcacaggccaaggccaaggccacggtcatggt
tcctgccaaggttccccggaaagcggtga

Start Met codon mutation

Premature termination codons in 1st and 4th exon + NMD

Muntiacus crinifrons

R.TQRSSFSLGRWSLWLLRLGLVLPSASAQALSCREAVLHTGDCINDRSTEANLYCLLELDSPPKDVEDHSAPKPVSFRVKETICPRTSQQPPEQCDFKENGLVKQCVGTVSLDQSEDQFDLNCNELQSVR
AKRPQPPKPRPPHRPRPRPRSCFLSRFPGKW

GenBank: SJXS01011712.1

cggtgaacccagaggtccagcttctccctggacggtggtcactatggctactgcggctgggactagtgctgccctcggcctctgcccaggccctcagctgcagggaggccgtgcttcacactggggattg
catcaatgacaggtccacagaagctaatctctactgcctcttggagctagactcacctcccaaggatgtgagttggagaggggctgagccatctcccctcagccttggtcacattatccctcccttcgct
caggctgttcctcctgtcaggaaagcacttctccctctaggtgggctctcacctcttcaggaaccttcccagaactgggaccctcccagccccaggcttcctgccttagcatctctgctgtgggaacagg
tgccctgcacacctggctcccaggacttccgggagctccagggatggaggggtcacaggctctgtgaggtgatgtccctgctgaagcccctctgcactgcggtgtctccctgccagagaggcctctgtca
gcctggaggttccaggtacaagggctctccctgcaggcggccccgacctccctcagcccctctgcggggaggcgctgccctcagcgctgctgtgtgatccgctcctgccctctgtgagcccttgaggccgg
ggacgggctctgtcccctccactgggctcccagcaccaagcctggggccggacacacaggggctggagaggctgccgtccgggtggggcggggagacagatcagagaaggaaagatgagcccgagtcca
gtctccccactttgatctttgaccaggtggaggaccacagtgctccaaagcctgtgagcttcagggtgaaggagaccatatgccccaggacgagccagcagcccccagagcagtgtgacttcaaggagaac
ggggtgagcctggggctggaggctgagggctgggataaatgcttctcagcgcaagttgagcaggggccttcagggaagatttccagcccctgaggggtgaggttggctgagcctgggaggttatggcctg
ggggttccagtttgacctggaaattccccttccagctggtgaaacagtgtgtgggaacagtcagcctggaccagtccgaagaccagtttgacctaaactgtaatgaggtgagtggccccttctgtgttgt
ggagatgctaacagggtgggttgtggaacatcctttggaccaatgacccgctgctccatccagggcagagaaaggccctcctacccaggcccaccctccccaatctgcaggtctccagccctggctctgt
atcctttagagaagtggctgtctaaaggggtcctcacccaggaactgacagtaaggcagattctcagccctgctgagatctcctgaatcagactttggggtggggccgaggcatttgtattttcacaaggc
ctccaggggattctgacaaagctgaagttgtgacaccctgatgcgagtcatggtcttgaggccatgctccagtccatctttgcccagatgggcttgtgacatttggaagctccttgtcatctctgggatca
gtttccccatatgttgtgggtttaaggattcaaccacatgctccaaagatcaccgccagaggatgagctggggccaaagttcctttggtggcccagtttggggggttgttcaggtggggagagagtggtctt
ctcttgacccttgcccagtcccacaagtaatctcttccattgtggttcacagcttcagagtgtcagggctaaacgacctcagcctcccaagcctcggcctcctcacaggccaaggccaaggccacggtcat
gcttcctgtcaaggttccctggaaagtggtga

Start Met codon mutation

Premature termination codons in 1st exon + NMD

Muntiacus gongshanensis

R.TQRSSFSLGRWSLWLLRLGLVLPSASAQALSYREAVLHTVDCINDRSTEANLYCLLELDSPPKDVEDHSAPKPVSFRVKETICPRTSQQPPEQCDFKENGLVKQCVGTVSLDQSEDQFDLNCNELQSVR
AKRPQPPKPRPPHRPRPRPRSWFLSRFPGKW

GenBank: JAGTXU010000095.1

ctgctggtgacactccccgtggggtccaggagaggccaaggagttgtcagggagatggggaaaacctttcttcaccttgctcaggggcctccctgcccactgcccagaaatcccatgaagcaacagcaggg
gcagaggcaagtcaatctcacaccctggaaggagggcaggggtggggtgggtcaggaagactcctggttgagcctttgcatcaggacccaggctgagcataaaaggagggtccctcgggctgggaggaggc
aggctgggggacggtgaacccagaggtccagcttctccctgggacggtggtcactatggctactgcggctgggactagtgctgccctcggcctctgcccaggccctcagctacagggaggccgtgcttcaca
ctgtggattgcatcaatgacaggtccacagaagctaatctctactgcctcttggagctagactcacctcccaaggatgtgagttggagaggggctgagccatctcccctcagccttggtcacattatccc
tcccttcgctcaggctgttcctcctgtcaggaaagcacttctccctctaggtgggctctcacctcttcaggaaaccttcccagaactgggaccctcccagccccaggcttcctgccttagcatctctgctg
tgggaacgggtgccctgcacacctggctcccaggacttccgggagctccagggatggaggggtcacaggctctgtgaggtgacgtccctgctgaagcccctctgcactgcggtgtctccctgccagggag
gcctctgtcagctctaggttccaggtacaagggctctccctgcaggcggccccgacctccctcagcccctctgcggggaggcgctgccctcagtgctgctgtgcggtccgctcctgccctctgtgagccct
tgaggccggggacgggctctgtcccctcccctgggctcccagcaccaagcccggggccggacacacaggggggctggagaggctgccgtccgggtggggcggggagacagatcagagaaggaaagatgagc
ccgagtccagtctccccactttgatctttgaccaggtggaggaccacagtgctccaaagcctgtgagcttcagggtgaaggagaccatatgccccaggacgagccagcagcccccagagcagtgtgacttc
aaggagaacggggtgagcctggggctggaggctgagggctgggataaatgcttctcagcgcaagttgagcaggggccttcagggaagatttccagcccctgaggggtgaggttggctgagcctgggaggt
tatggcctgggggggttccagtttgacctggaaactccccttccagctggtgaaacagtgtgtgggaacagtcagcctggaccagtccgaagaccagtttgacctaaactgtaatgaggtgagtggcccctt
ctgtgttgtggagatgctaacagggtgggttgtggaacatcctttggaccaatgacccgctgctccatccagggcagagaaaggccctcctacccaggcccaccctccccaatctgcaggtctccagccc
tggctctgtatcctttagagaagtggctgtctaaaggggtcctcacccaggaactgacagtaaggcagatctcagccctgctgagatctcctgaatcagactttggggtggggcccaggcatttgtatttt
cacaaggcctccaggggattctgacaaagctgaagttgtgacaccctgatgtgagtcatggtcttgaggccatgctccagtccatctttgcctggatgggcttgtgacatttggaagctccttgtcatctc
tgggatcagtttccccatatgttgtgggtttaaggattcaaccacatgctccaaagatcaccgccagaggatgagctggggccaaagttcctttggtggcccagtttggggggttgttcaggtggggagaga
gtggtcttctcttgacccttgcccagtcccacaagtaatctcttccattgtggttcacagcttcagagtgtcagggctaaacgacctcagcctcccaagcctcggcctcctcacaggccaaggccaaggcc
acggtcatggttcctgtcaaggttccctggaaagtggtgaagaactggctatcacacctattaatggcttttggtgcatcctgagcctggggaagtgttttaaagatctgatttgttctgtctcagacttc
tggatggtgaaaaataaattcttgtgaaatcagcttcctccagccttcaatttcaattatttccctttctcccacttactgagtccttgactctgagaggcctcctctctgtttatgtg

Start Met codon mutation

Premature termination codons in 1st exon + NMD

Muntiacus reevesi

R.TQRSSFSLGRWSLWLLRLGLVLPSTSAQALSYREAVLHTGDCINDRSTEANLYCLLELDSPPKDVEDHSAPKPVSFRVKETICPRTSQQPPEQCNFKENGLVKQCVGTVSLDQSEDQFDLNCNELQSVR
AKRPQPPKPQPPHRPRPRPRSLFLPRFPGKR

GenBank: SJXT01015098.1

cggtgaacccagaggtccagcttctccctggacggtggtcactatggctactgcggctgggactagtgctgccctcgacctctgcccaggccctcagctacagggaggccgtgcttcacactggggattg
catcaatgacaggtccacagaagctaatctctactgcctcttggagctagactcacctcccaaggatgtgagttggagaggggctgagccatctcccctcagccttggtcacattatccctcccttcgct
caggctgttccgcctgtcaggaaagcacttctccctctaggtgggctctcacctcttcaggacaccttcccagaactgggaccctcccagccccaggcttcctgccttagcatctctgctgtgggaacagg
tgccctgcacacctggctcccaggacttccgggagctccagggatggaggggtcacaggctctgtgaggtgacgtccctgctgaagcccctctgcactgcggtgtctccctgccagggaggcctctgtca
gctctaggttccaggtacaagggctctccctgcaggcggccccgacctccctcagcccctctgcggggaggcgctgccctcagcgctgctgtgcggtccgctcctgccctctgtgagcccttgaggccggg
gacgggctctgtcccctcccctgggctctcagcaccaagcctggggctggacacacaggggggctggagaggctgccgtccgggtgggggcggggagacagatcggagaaggaaagatgagcccgagtccag
tctccccactttgatctttgaccaggtggaggaccacagtgctccaaagcctgtgagcttcagggtgaaggagaccatatgccccaggacgagccagcaacccccagagcagtgtaacttcaaggagaatg
gggtgagcctggggggctggaggctgagggctgggataaatgcttctcagcgcaagctgagcaggggccttcagggaagatttccagcccctgagggtgaggttggctgagcctgggaggttatggcctgg
ggggttccagtttgacctggaaactccccttccagctagtgaaacagtgtgtggggacagtcagcctggaccagtccgaagaccagtttgacctaaactgtaatgaggtgagtggccccttctgtgttgtg
gagatgctaacagggtgggttgtggaacatcctttggaccaatgacccgctgctccatccagggcagagaaaggccctcctacccaggcccattctccccaatctgcaggtctccagccctggctctgta
tcctttagagaagtggctgtctaaaggggtcctcacccaggaactgacagtaaggcagattctcagccctgctgagatctcctgaatcagactttggggtggggcccaggcatttgtattttcacaaggcc
tccaggggattctgacaatgctgaagttgtgacaccctgatgcaagtcatggtcttgaggccatgctccagtccatctttgcccggatgggcttgtgacatttggaagctccttgtcatctctgggatcag
tttccccatatgttgtgggtttaaggattcaaccacatgctccaaagatcaccgccagaggatgagctggggccaaagttcctttggtggcccagtttggggggttgttcaggtggggagagagtggtcttc
tcttgacccttgcccagtcccacaagtaatctcttccattgtggttcacagcttcagagtgtcagggctaaacgacctcagcctcccaagcctcagcctcctcacaggccaaggccaaggccacggtcatt
gttcctgccaaggttccccggaaagcggtga

Start Met codon mutation

Premature termination codons in 1st exon + NMD

Hydropotes inermis

VKTQRASLSLGRWSLWLLLLGLVLPSACAQAPSYREAVLRAVDRINDRSSEANLYRLLELDPPPKDVEDQGARKPVSFRVKETVCPKTSQQPPEQCDFKENGLVKQCVKTASLDWSDDQFDINCNELQSVR
AK.PRPPKPQPPHRPRPRPRPPWFLPRFPGKW

GenBank: SPDZ01001416.1

gtgaagacccagagggccagcctctccctgggacggtggtcactgtggctactgctgctgggactggtgctgccctcggcctgtgcccaggcccccagctacagggaggcagtgcttcgcgctgtggatcg
catcaatgaccggtcctcagaagctaatctctaccgcctcctggagctagacccgcctcccaaggatgtgagctggggagggggctgagccatctccccccagcctcggtcacattatccctccctttgct
caggctgtacctcctgtcaggaaggcacttctccctctaggtgggctcccacctcttccagaaaccttcccagacctgggtcccctgccagccccaggcttcctgccttagcctctctgctttgggaaga
ggcgccctgcacacctggctcccagcacttctgggagctccggggatggaggggtcacaggctctgtgaggggaagtccctgctgaagcccctctgcaccgcggtgtctccctgccagggaggcctctgt
cagcctggaggttccaggtacaagggctctccctgcaggcggccctgagctccctcagcccctctgcggggaggcgctgccctcagagctgccgtgaggccggggacgggctctgtcccacccctgggctc
ccagcaccaagcccaggcttgggtggggcagggagacagatcagagaaggaaacatgagcctgagcccagtctccccactttgatctttgaccaggtggaagaccagggagctcgaaagcctgtgagct
tcagggtgaaggagaccgtgtgccccaagacgagccagcagcccccagagcagtgtgacttcaaggagaatggggtgagcctggggctggaggctgagggctgggataaatgcttctcagcgcaagctga
gcaggggccttcagggatgatttccagcccatgaggggtgaggttggctgagcctggggggttatggccccggggggttccagtttgacctggaaactccccttccagctggttaaacagtgtgtgaagaca
gccagcctggactggtccgatgaccaatttgacataaactgtaatgaggtgagtggccccttctgtgttgtgcagatgctaacagggtaggttgtggaacatcctttggaccaatgacctgatgctccatc
cagggcagagaaaggccctcctacctgggcccaccctccccaatccccaggtctccagccctggctctgcttcctttagagaagtggctgtctaatggggtcgccacccaggaaccgacagtaaggcaga
ttctcagccccactgaagcctgctgaatcagactttggggtggggcccaggcatttgtatttttacaaggcctctggggattctgacagtgctgaagttgtgacaccctgactcgagtcatggtcttgag
gccatgctccagtccatctttgcccggatgggcctgtgacacttggaagccccttgtcatctctgggatcagtttccccatatgttgtgggtttaaggattcaaccacatgctccaaagatcacggccaga
ggatgatctggggccaaagttcctttggtggcccagtttggggggctgttcaggtggggagagagtggtcttctcttgacccttgcccagtcccacaagtgatctcttccattgtggttcacagcttcagag
tgtcagggctaaatgacctcggcctcccaaacctcagcctcctcacaggccaaggccaaggccaaggccaccttggttcctgccaaggttccccggaaaatggtga

Start Met codon mutation

Premature termination codons in 4th exon + NMD

Rucervus eldii

RTQRASLSLGWWSLWLLRLRLVLPLASAQALSYREAVLHTGDCINDGSTEANLYRLLELDPPPEEDVEDHSARKPVSFRVKETICPRTSQQPPEQCDFKENGLVKQCVGTASLDQSDDQFDLNCNELQSVR
AK.PQPPKPRPPHRPRPRPRSWFLPRFPGKR

GenBank: JACCHN010000245.1

agggacccagagggccagcctctccctgggatggtggtcactgtggctactgcggctgagactagtgctgcccttggcctctgcccaggccctcagctacagggaggccgtgcttcacactggggattgcat
caatgacgggtccacagaagctaatctctaccgcctcctggagctagacccgcctcctgaggaggatgtgagttggggaggggctgagccatctcccccagccttggtcacattatccctcccttcgct
caggctacctcctgtcaggaaggcactttgccctctaggtgggcttccacctcttccagaaaccttcccagacctgggtcccctgccagccccaggcttcctgccttagcatctctgctgtgggatcagg
cgccctgcacacctggctcccaggacttccgggagctccagggatggaggggtcacaggctctgtgaggtgacgtccctgctgaagcccctctgcactgcggtgtctccctgccagggaggcctctgtca
gcctggacgttccaggtacaagggctctccctgcaggcggccccaacctccctcagcccctctgcggggaggcgctgccctcagcgttgccgtgcggtccgctcctgctctctgggtgctcgtgaggccgg
ggacggcctctgtcccctcccctgggctcccagcaccaagcccagggctggacacacaggggctggagaggctgccgtctgggttggggcggggagacagatcagagaaggaaacatgagcccgggtcca
gtcctgccactttgatctttgaccaggtggaggaccacagtgctcgaaagcctgtgagcttcagggtgaaggagaccatatgccccaggacgagccagcagcccccggagcagtgtgacttcaaggagaat
ggggtgagcctggggctggaggctgagggctgggataaatgcttctcagcacaagctgagcaggggacttcagggaagatttccagcccctgaagggtgaggttggctgagcctggaaggttatggcccc
ggggttccagtttgacctagaaactccccttccagctggtgaaacagtgtgtggggacagccagcctggaccagtctgatgaccagtttgacctaaactgtaatgaggtgagtggccccttctgtgttgt
gcagatgctaacagggtagattgtggaacatcgtttggaccaatgacccgatgccccatccagggcagagaaaggccctcctacccaggcccaccctccccaatccccagctctccagccctggctctgt
atcctttagagaagtggctgtctaaaggggtcctcacccaggaactgacagtaaggcagattctcagccctactgagatctcctgaatcagactttggggtggggcccaggcatttgtattttcacaaggc
ctccaggggattctgacagtgctgaagttgtgacaccctgactcgagtcatggccttgaggacatgctccagtccatctttgcccggatgggcttctgacacttggaagctccttgtcatctctgggatca
gttcccccatatgttgtgggtttaaggattcaaccacatgctccaaagatcaccgccagaggatgagctggggccaaagttcctttggtggcccagtttggggttgttcaggtgggagagagtggtctt
ctcttgacccttgcccagtcccacaagtaatcgcttccattgtggttcacagcttcagagtgtcagggctaaatgacctcagcctcccaaacctcggcctcctcacaggccaaggccaaggccacggtcat
ggttcctgccaaggttccctggaaaacggtga

Start Met codon mutation

Premature termination codon in 4th exon + NMD

Alces alces

METQRASLSLGRWSLWLLLLGLVLPSASAQALSYREAVLRTGDRINDGSTDANLYHLXELDSPPKDVGDRGARKPASFTEKETVCPRSSQ.PPEQCDFKENGLVKQCVGSASLDQSDDQFDINCNELQSVR
AK.PRPPKPQPPHRPRPRPWPCFLPRFPGKW

GenBank: JADEYB010002473.1

atggagacccagagggccagcctctccctgggacggtggtcgctgtggctactgctgctgggactggtgctgccctcggccagcgcccaggccctcagctacagggaggccgtgcttcgcactggggatcg
catcaatgacggatccacagacgctaatctctaccacctct__gagctagactcacctcccaaggatgtgagttggagaggggctgagccatctccccccagccttggtcacactatccctcccttcgct
caggctgtacctccggtcaggaaggcacttctccctctaggtggctcccacctcttccagaaaaccttcccagacctgggaccctgccagccccaggcttcctgccttagcatctctgctgcgggaacagg
cgccctgcacacctggctcccaggacttccgggagctcccgggatggaggggtcataggctctgtgaggggacgtccctgctgaagcccctctgcacagcggtgtctccctcccagggaggcctctgtca
gcctggaggttccaggtccaagcgctctccctgcaggcggccccgacctccctcagcccctctgcggggaggcgctgccctcagcgctgccgcgcggtctgctcctgctctctgtgagcccttgaggccgg
gacgggctctgtccctcccctgggctcccagcaccaagcccagggccggacacgcaggggggctggagaggctgccgtctgggtgggggcagggagacagatcagagacggaaacacgagcccgagcccagt
ctccccaccttgatctttgaccaggtgggggaccgggggagctcgaaaacctgcgagcttcacggagaaggagaccgtatgccccaggtcgagccagtagcccccggagcagtgtgacttcaaggagaatg
ggGTgagcctggggggctggaggctgagggctgggataaatgcttctcagcgcaagctgagcaggggtcttcagggatgatttccagctcctgagggggtgaggctggctgagcctgagaggttatggccccg
ggggttccagtttgacctggaaactccccttccagctggtgaaacagtgtgtggggtcagccagcctggaccagtccgatgaccaatttgacataaactgtaatgaggtgagtggccccttctgtgctgtg
cagatgctaacagggtaggttgtggaacatcctttggaccagtgacctgatgccccatccagggcagagaaaggccctcctacctgggcccaccctccccaatccccaggtctccagccctggctctgca
tcctttagagaagtggctgtataatggggtcgccacccaggaactgacagtaaggcagattctcagccccactgaagcctgctgaatcagactttggggtggggcccaggcatttgtatttttacaaggcc
tctgggggattctgacagtgctgaagttgtgacaccccgactcgagtcatggtcttgaggccatgctccagtccatctttgcccggatgggcttgtgacacttggaagccccttgtcatctctgggatcag
tttccccatatgttgtgggtttaaggattcaaccacatgctccaaagatcactgccagaggatgatctggggccaaagttcctttggtggcccagtttggggggttgttcaggtggggagagagtggtcttc
tcttgacccttgcccagtcccacaagtaatctcttccatggtggttcacagcttcagagtgtcagggctaaatgacctcggcctcccaaacctcagcctcctcacaggccaaggccaaggccatggccatg
tttcctgccaaggttccccggaaaatggtga

Indel (2 bp deletion in 1st exon) + (1 bp insertion in 2nd exon) – nonsense mutations

Premature termination codons in 1st,2nd and 4th exon + NMD

Rangifer tarandus

VKTQRASLSLGRWSLWLLRLRLVLPSASAQALSYREAVLRTGNRINDGSTEAYLYCLLELDSPPKDVEDRSARKPVSFRVKETVCPKMSQQPAEQCDFKENGLVKQCVGTASLDWSDDQFDINCNELQSVR
AK.PQPPKPQPPHRPRPRPRPWFLPRFPGKW

GenBank: CAUJRY010019149.1

gtgaagacccagagggccagcctctccctgggacggtggtcactgtggctactgcggctgagactagtgctgccctcggcctctgcccaggccctcagctacagggaggccgtgcttcgcactgggaatcg
catcaatgacgggtccacagaagcttatctctactgcctcctggagctagactcacctcccaaggatgtgaggtggggagggggctgagccgtctccccccagccttggtcacattatccctcccttcgct
caggctgtacctcctgtcaggaaggcactttgccctctaggtgggctcccacctcttccagaaaaccttcccagacctgggtcccctgccagcccccggcttcctgccttagcatctctgctgcgagaaca
ggcgccctgcacacctggctcccaggacttccgggagctccagggatggagggggtcacaggctctgtgagggggatgtccctgctgaagcccctctgcaccgtggtgtctccctgccagggaggcctctgt
cagcctggaggttccaggtacaagcgctctccctgcaggtggccccgacctccctcagcccctctgcggggaggcgctgccctcagcgctgctgtgcggtccgctcctgctctctgtgagcccgtgaggcc
ggggacgggctctgtcctctcccctgtgctcccagcaccaagcccagggctggacacgcaaggggctggagaggctgccgtctgggtgggggcagggagacagatcagagaaggaaacatgagcccgagcc
cagtcccccccactttgatctttgaccaggtggaggaccgcagtgctcgaaagcctgtgagcttcagggtgaaggagaccgtgtgccccaagatgagccagcagcccgcggagcagtgtgacttcaaggaga
atggggtgagcctggggctgggataaatgcttctcagcgcaagctaagcaggggccttcagggaagatttccagcccctgaggggtgaggttggctgaggctggaaggttatggccccggggggttccagt
ttggcctggaaactccccttccagctggtgaaacaatgtgtggggacagccagcctggactggtccgatgaccaatttgacataaactgtaatgaggtgagtggccccttctgtgttgtgcagatgctaac
agggtaggttgtggaacatcctttggaccaatgacctgatgccccatccagggcagagaaaaggcccttctacctgggcccaccctccccaatccccaggtctccagccctggctctgcatcctttagaga
agtggctgtctaatagggtcgccacccaggaactgacactaaggcagattctcagccccactgaagcctgctgaatcagactttggggtggggcccaggcatttgcatttttacaaggcctctggggatt
ctgacagtgctgaagttgtgacaccctgacttgagtcatggtcttgaggccatgctctagtccatctttgcccagatgggcttgtgacacttggaagccccttgttatctctgggatcagtttccccatat
gttgtgggtttaaggattcaaccacatgctccaaagatcaccaccagaggatgatctggggccaaagttcctttggtggcccagtttggggggttgttcaggtggggagagagtggtcttctcttgaccctt
gcccagtcccacaagtaatctcttccattgtggttcacagcttcagagtgtcagggctaaatgacctcagcctcccaaacctcagcctcctcacaggccaaggccaaggccacggccatggttcctgccaa
ggttccccggaaaatggtga

Start Met codon mutation

Premature termination codons in 4th exon + NMD
